# Racemic Total Synthesis of Elmonin and Pratenone A, from *Streptomyces*, Using a Common Intermediate Prepared by *peri*-Directed C–H Functionalization

**DOI:** 10.1021/acs.orglett.2c03449

**Published:** 2022-12-19

**Authors:** Michiel T. Uiterweerd, Adriaan J. Minnaard

**Affiliations:** University of Groningen, Stratingh Institute for Chemistry, Nijenborgh 7, 9747 AG Groningen, The Netherlands

## Abstract

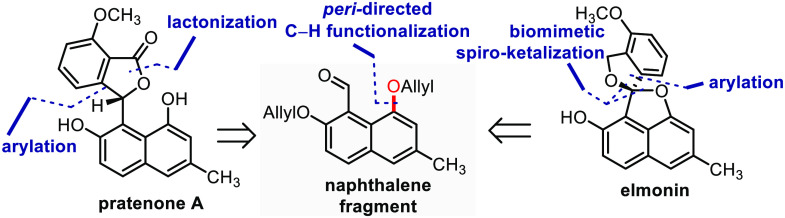

The first total synthesis of elmonin and pratenone A, two complex rearranged angucyclinones from *Streptomyces*, is reported. Using *peri*-directed C–H functionalization, the key naphthalene fragment present in both synthetic targets was efficiently prepared. Coupling to two anisole-derived fragments gave access to the natural products, in which elmonin was prepared using a biomimetic spiro-ketalization.

Elmonin^1,2^ (**1**) and pratenone A^3^ (**2**) (Figure 1) are C-ring-cleaved, rearranged angucyclinone polyketides from *Streptomyces*. **1** is a spiroketal with a spiro[isobenzofuran-1,20-naphtho[1,8*b*,*c*]furan] skeleton and has been independently isolated by two groups. In the report by the group of Ishibashi,^1^**1** (from *Streptomyces* sp. IFM11490) was named elmonin, although in an earlier report by the group of Müller^2^**1** (from *Streptomyces* sp. Lv20-195) had been called oleaceran.

**Figure 1:**
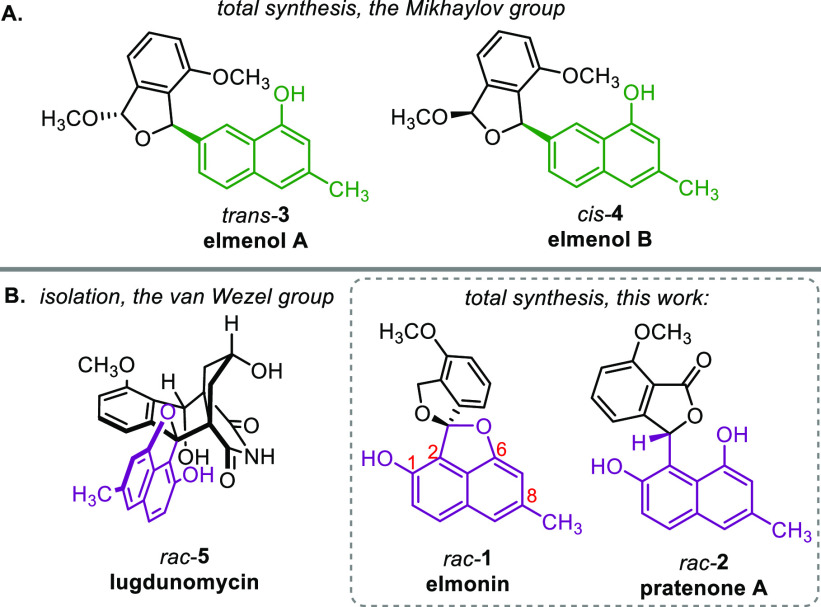
(A) Elmenol A and B. (B) Rearranged angucyclinones lugdunomycin, elmonin, and pratenone A, carrying the same 1,2,6,8-tetrasubstituted naphthalene motif (purple). Only one enantiomer is shown.

The absolute configuration of natural **1** is unknown. The groups reported opposite optical rotations, based on measurements on diluted natural isolates, which arguably can lead to erroneous conclusions due to the presence of optically active impurities. Müller et al. reported an [α]_D_ of +36 (*c* 0.01, MeOH), while Ishibashi et al. reported an [α]_D_ of −10 (*c* 0.3, MeOH) and stated that they isolated the opposite enantiomer. A putative biosynthetic pathway of **1** was proposed in 2016 by the group of Ishibashi and includes a spiro-ketalization.^4^ This process can be either spontaneous or enzymatic, although enzymatic spiro-ketalization inducing enantio-enrichment is rare.^5^ The presence of opposite enantiomers of a compound in two strains of the same genus is less likely, but not uncommon.^6^ Spontaneous spiro-ketalization, on the contrary, will obviously yield a racemate. **1** possesses moderate antifungal activity, is a weak antibiotic, and shows cyto-toxicity against HCT-116 cells.^2^

Pratenone A **2** has been identified in marine-derived *Streptomyces pratensis*. It was the first isolated angucyclinone with a 3-(naphthalen-1-yl)isobenzofuran-1(3*H*)-one skeleton and shown by chiral HPLC to be racemic.^3^ Pratenone A **2** has been tested for antimicrobial activity and displayed growth inhibition for *Staphylococcus aureus* with a minimum inhibitory concentration (MIC) of 8.0 μg/mL.^3^

The synthesis of C-ring-cleaved, rearranged angucyclinones is a largely unexplored topic in organic chemistry. In 2021, the group of Mikhaylov reported the first total synthesis of elmenol A *trans*-**3** and B *cis*-**4**, and related compounds.^7^ This is to date the sole report, and the structure of elmonin and pratenone A is considerably more complex, because of the substituted naphthalene core, and in the case of elmonin the spiroketal unit.

Our studies of the structure of rearranged angucyclinone derivatives such as lugdunomycin^8^**5** sparked our interest in embarking on a racemic total synthesis of **1** and **2**. We were in particular intrigued by the 1,2,6,8-tetrasubstituted naphthalene motif that many rearranged angucyclinones have in common.^9^ Preparation of a common intermediate comprising this naphthalene motif would allow us to access **1** and **2** and would in subsequent studies serve as a basis for preparing a large diversity of rearranged angucyclinones. In addition, we were triggered to test the proposed spiro-ketalization in the biosynthetic pathway of elmonin **1** by mimicking this reaction non-enzymatically.

Considering a retrosynthetic analysis of **1** (Scheme 1A), we anticipated preparation using the ketalization of **6**, which can be accessed by oxidation of benzhydrol **7**. As envisioned, this compound in turn can likely be prepared by means of arylation of naphthalene fragment **9** with lithiated anisole fragment **8**. In parallel, we considered that compound **2** can be prepared from **11**, which in turn can be synthesized by performing an arylation of naphthalene fragment **9** with magnesiated anisole fragment **12**, prepared using a procedure based on work by the group of Knochel.^10,11^

**Scheme 1:**
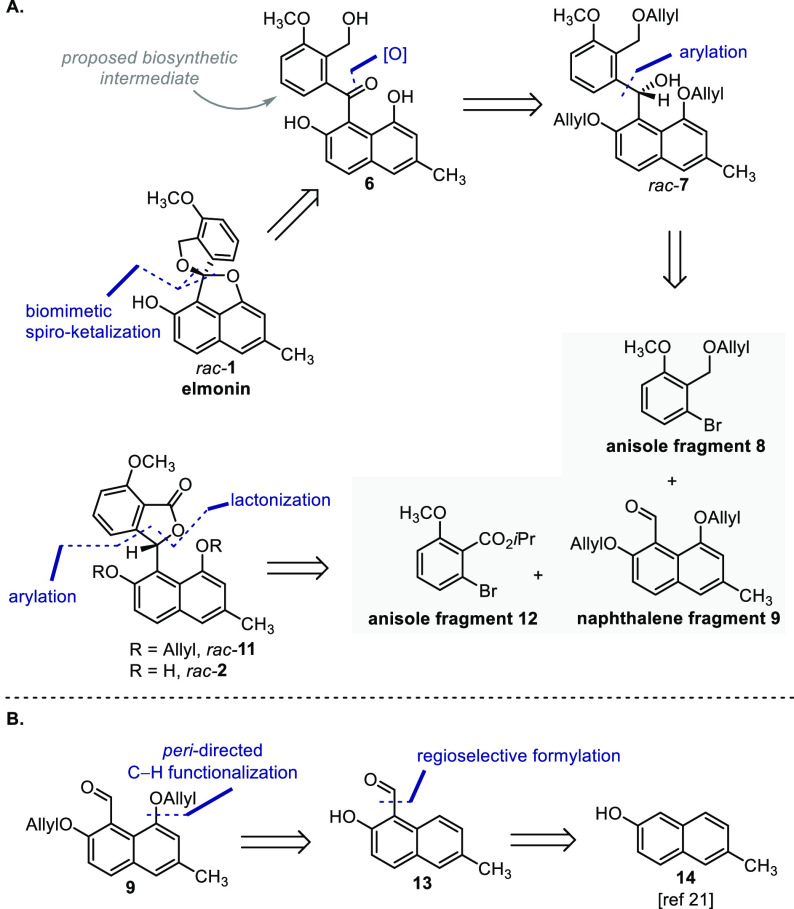
Retrosynthetic Analysis of **1** and **2**

As for naphthalene fragment **9**, *peri*-functionalization of **13**, or a suitable analogue thereof, was envisaged (Scheme 1B). *peri*-Functionalization of naphthalenes has been achieved by directed lithiation^12,13^ and transition metal-catalyzed C–H functionalization.^14−18^ As none of these methods use α-naphthaldehydes or derivatives thereof, applying these methods would lengthen our route. The group of Sanford, however, reported the use of oxime derivatives of aldehydes and ketones as substrates for *ortho*-directed C–H acetoxylation.^19,20^ Intrigued by this elegant procedure, we planned to extend the method using the oxime ether of **13**. The required aldehyde **13** in turn was expected to be prepared by regioselective electrophilic formylation of naphthol **14**, which is a known compound.^21^

Our synthesis started with the preparation of naphthalene **9** (Scheme 2). The preparation of the starting material for this fragment, 6-methylnaphthalene-2-ol **14**, has been described by Verga et al.^21^ Slight modifications of the literature procedure were necessary for scale-up, which allowed the conversion of 50 g of commercially available 6-bromonaphthalene-2-ol **15** into **14** in nearly quantitative yield by bromo-lithium exchange, followed by methylation. The crude product was used as such in a Rieche formylation, effected by addition of α,α-dichloromethyl methyl ether and TiCl_4_ in dichloromethane.^22,23^ The obtained naphthol-aldehyde was protected as the methyl ether^24^ and then converted into the corresponding oxime ether **16**,^25^ in 79% yield over four steps, after a single chromatography run. We were pleased to see that treatment of **16** with 5 mol % Pd(OAc)_2_ and stoichiometric oxone^19^ provided acetoxylated product **17** in an acceptable 47% yield as a 1:1 inseparable mixture of oxime isomers. This result is comparable with phenyl-derived aldoxime ether substrates.^19^ The isomerization of the oxime ether is likely thermally induced.^26^ In the course of our work, Jiang et al. published a general but similar method for the palladium-catalyzed *peri*-hydroxylation of α-naphthaldehydes, using substoichiometric amounts of amines as the directing group (via the imine) and PhI(O_2_CCF_3_)_2_ as the oxidant.^27^ Although this in general provides higher yields, we decided to adhere to the oxime-ether/oxone reaction because of the far lower costs of the reagents and the straightforward purification of the product on a multigram scale.

**Scheme 2:**
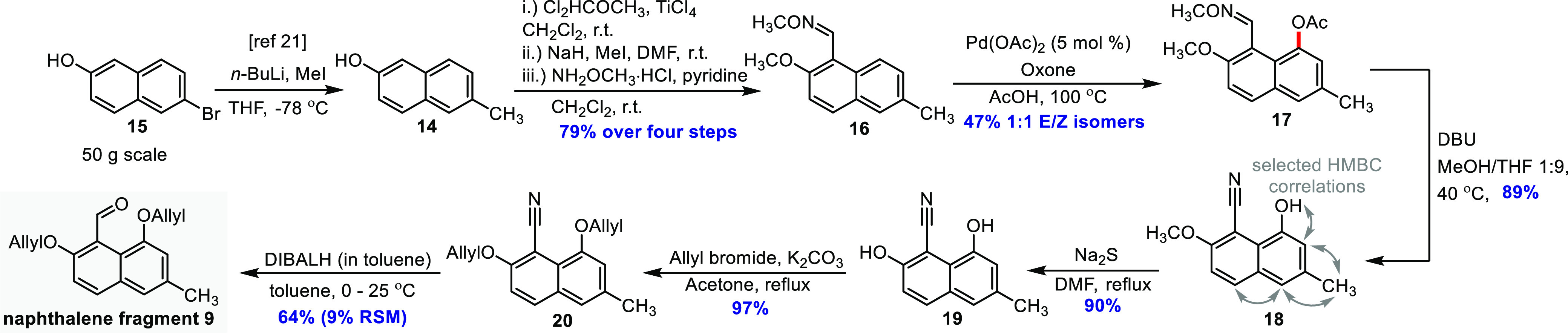
Synthesis of Naphthalene Fragment **9**

To perform subsequent functional group manipulations, **17** had to be deacetylated and converted into the corresponding aldehyde. Basic saponification with K_2_CO_3_ in MeOH,^24^ however, not only led to deacetylation but also eliminated the oxime ether to the corresponding nitrile **18**. We believe initial deacetylation gives a phenolate that induces intramolecular β-elimination. A similar reaction has been studied by Asaad et al., using *peri*-substituted dialkylamino-oxime benzyl ethers.^28^ The proximity of the reacting functional groups results in a high effective molarity, which was studied by Engberts and Kirby.^29^ We anticipated **18** could be useful for our synthesis, so we optimized the reaction. Using a MeOH/THF solvent mixture and DBU as a base led to an isolated yield of 89%.

The next step was demethylation of **18**, which proved to be a challenging exercise. Treatment of **18** with common Lewis acidic demethylation reagents^24^ like BBr_3_ and BBr_3_·S(CH_3_)_2_ did not affect the transformation, and only starting material was recovered. We anticipated that the substrate is deactivated by a combined mesomeric push–pull effect of the methyl ether and the nitrile, respectively, hindering coordination of the boron reagent. To circumvent the problem, nucleophilic conditions were considered. A known method for converting aromatic ethers into their corresponding phenols is strongly heating of the ether with neat MeMgI or MeMgBr.^24,30^ These conditions, however, are not suitable due to the presence of the nitrile. Other procedures make use of thiolate/sulfide reagents, such as EtSNa, anhydrous Na_2_S, or PhSH/Et_3_N, and these conditions seemed to be more feasible.^24^ When **18** was treated with anhydrous Na_2_S in refluxing DMF,^24,31^ demethylation was indeed observed and **19** was obtained in 90% isolated yield. After this successful demethylation, double reprotection was now required, which was effected by a reaction with allyl bromide,^24^ affording bis-allyl-protected **20** in 97% yield. Allyl ethers were chosen as protecting groups at both positions because of their stability under nucleophilic conditions, required in the later fragment assembly stage, and because their deprotection can be brought about by treatment with a wide range of Pd-based reagents, which is well established in the literature.^24^ The reduction of the nitrile, performed with DIBALH, required optimization. With DIBALH in DCM, the reaction gave poor yields, accompanied by the formation of highly polar side products. When performed in toluene at 0 °C to rt, the reduction proceeded more efficiently, and although 9% starting material was recovered, the isolated yield was 64% after chromatography. We argue that the somewhat sluggish reduction with DIBALH is largely the consequence of a mesomeric push–pull effect, caused by the nitrile and the allyl ether functionality, causing a decreased electrophilicity of the nitrile carbon.

With **9** in hand, we attempted to synthesize target **2** using lactone **11**, which was, as planned, synthesized from **9** and anisole-derived **12**. Fragment **12** was obtained by reacting commercially available 2-bromo-6-hydroxybenzoic acid **21** with isopropanol using Mitsunobu esterification conditions (Scheme 3). Mitsunobu conditions were used because the ester could not be accessed from the corresponding acid chloride, which seemed to undergo rapid decomposition upon its generation, nor could the ester be obtained via standard Fischer esterification, as only starting material was recovered. Bromide fragment **12** was converted into a magnesiated species, using chemistry reported by the group of Knochel.^10^ First, the ester was treated with a mixture of the *i*PrMgCl·LiCl complex and 15-crown-5, to accomplish bromo-magnesium exchange. The freshly prepared organomagnesium reagent was then reacted with **9**, affording the desired **11** in 71% yield after chromatography. An alternative attempt to prepare **11** by a coupling of **9** and **21** using bromo-lithium exchange gave the product in a poor yield (see the Supporting Information). A batch of lactone **11** was converted into pratenone A **2** by deallylation. **11** was reacted with catalytic Pd(PPh_3_)_4_ in the presence of stoichiometric amounts of *N,N*-dimethylbarbituric acid (“DMBA”) as a nucleophilic scavenger, in MeOH.^24,32^ The natural product was obtained in 68% yield after chromatography, and an analytically pure sample was obtained after repeated trituration with toluene. The spectral data of the synthetic compound matched the reported data.

**Scheme 3:**
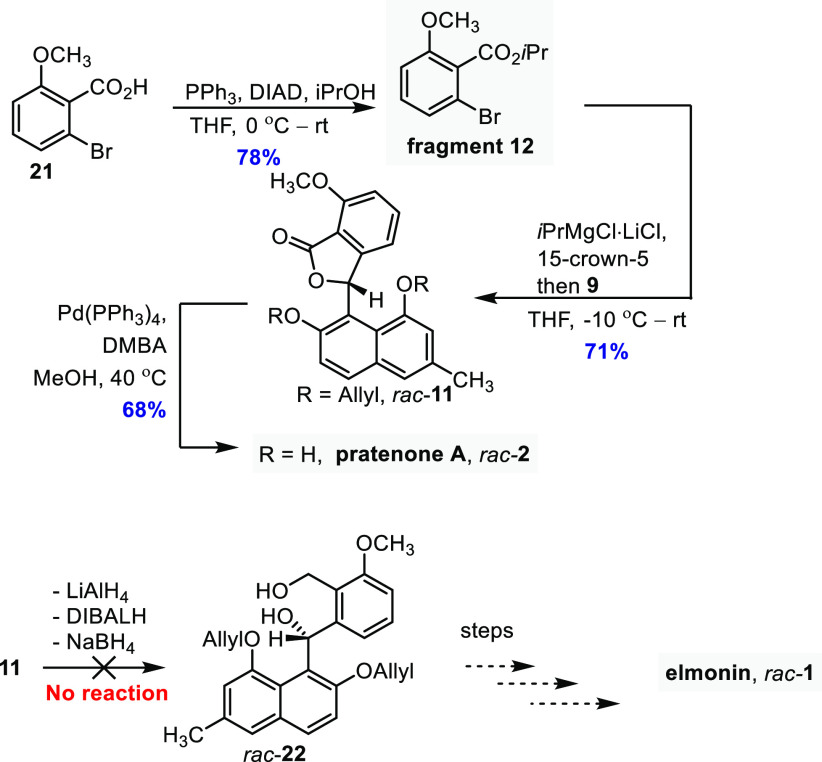
Synthesis of Pratenone A and a Failed Attempt to Synthesize Elmonin DMBA = *N,N*-dimethylbarbituric acid.

Next, we turned our attention to the synthesis of elmonin **1**. Deviating from our original plans, out of curiosity, we attempted to prepare benzophenone **22** by reduction of lactone **11**, which after several functional group manipulations would afford **1**. This, however, turned out to be not possible; to our surprise, the lactone proved to be highly resistant to reduction. Treatment with LiAlH_4_, DIBALH, and NaBH_4_ did not furnish the desired benzhydrol.

We argue that mesomeric electron donation of the methyl ether to the lactone carbonyl deactivates the substrate. Steric hindrance of the methyl ether may also contribute. We therefore continued to explore our originally planned strategy that we based on a cross-coupling of fragment **9** and anisole fragment **8**. The synthesis of **8** was effectively accomplished in 75% yield by O-methylation, reduction, and allyl protection, starting from bromo-salicylaldehyde **23** (Scheme 4). Next, a successful coupling between **8** and fragment **9** gave benzhydrol **7** in 73% yield. This was achieved by converting **8** into the corresponding aryl lithium species, using standard bromo-lithium exchange conditions with *n*-BuLi/TMEDA in THF and subsequent reaction with **9**.^33^ The benzhydrol was conveniently oxidized to benzophenone **24** in 86% yield by Dess-Martin periodinane (DMP) in DCM.^34,35^ A triple deallylation was then performed by treating **24** with catalytic Pd(PPh_3_)_4_ and stoichiometric *N,N-*dimethylbarbituric acid, which gave the proposed biosynthetic precursor of **1**, compound **6**, quantitatively. **6** appeared to be stable; however, when **6** was dissolved in aged samples of (presumably slightly acidic) CDCl_3_, cyclization to elmonin **1** was observed serendipitously. Successful reaction of **6**, giving **1**, supports the biosynthetic pathway suggested by Abdelfattah et al.^4^ and makes a non-enzymatic reaction likely. Unfortunately, and somewhat annoyingly, this process was difficult to reproduce on a preparative scale using CDCl_3_/CHCl_3_. Even after the mixture had been stirred for several days in the presence of water scavengers such as MgSO_4_, or 4 Å molecular sieves, complete cyclization could not be achieved. Also, continuous removal of water by distilling off the solvent did not push the cyclization to completion. Gratifyingly, it was found that the desired spiro-ketalization could be achieved in a synthetically useful 70% yield (Scheme 4), by treating **6** with catalytic amounts of hydrated iron(III) chloride in CH_2_Cl_2_ at room temperature. Hydrated ferric chloride has been used previously as a mild (Lewis) acid catalyst for numerous transformations.^36,37^ The spectroscopic data of the synthetic sample matched the reported data.

**Scheme 4:**
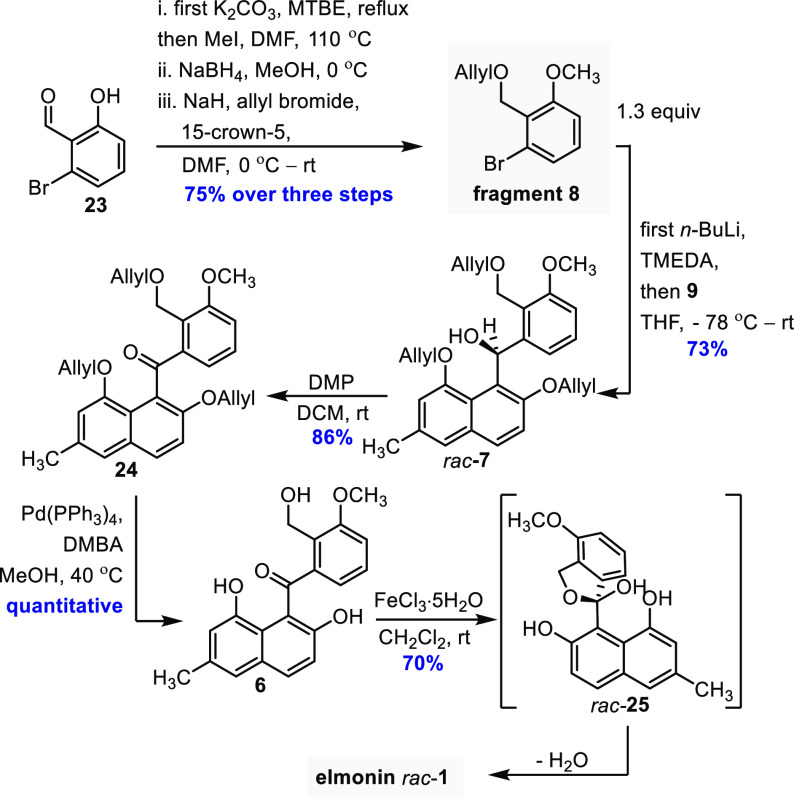
Synthesis of Elmonin DMBA = *N,N*-dimethylbarbituric acid.

In conclusion, we completed the first total synthesis of elmonin (**1**) and pratenone A (**2**), applying a convergent strategy, using naphthalene fragment **9** as a common intermediate. Fragment **9** was accessed, using a *peri*-directed C–H acetoxylation reaction as a key transformation. This is the first natural product synthesis utilizing *peri*-directed C–H functionalization, and we demonstrate that it is a powerful method for obtaining complex *peri*-substituted naphthalene units. We expect that **9** will be used to prepare a number of other rearranged angucyclinones in future studies, by varying the structure of the anisole-derived fragment. A key step in the synthesis of elmonin is the biomimetic spiro-ketalization of benzophenone **6**, which supports the biosynthetic pathway proposed in the literature and suggests that elmonin probably is formed *in vivo* as a racemate.

## Data Availability

The data underlying this study are available in the published article and its Supporting Information.

## Supporting Information Available

Experimental procedures and spectral data (PDF)

## References

[ref1] Yixizhuoma; Tsukahara K.; Toume K.; Ishikawa N.; Abdelfattah M. S.; Ishibashi M. Novel Cytotoxic Isobenzofuran Derivatives from Streptomyces Sp. IFM 11490. Tetrahedron Lett. 2015, 56 (46), 6345–6347. 10.1016/j.tetlet.2015.09.116.

[ref2] Raju R.; Gromyko O.; Fedorenko V.; Luzhetskyy A.; Müller R. Oleaceran: A Novel Spiro[Isobenzofuran-1,2′-Naptho[1,8- *Bc*]Furan] Isolated from a Terrestrial *Streptomyces* Sp. Org. Lett. 2013, 15 (14), 3487–3489. 10.1021/ol401490u. 23802119

[ref3] Zhang S.; Zhang L.; Kou L.; Yang Q.; Qu B.; Pescitelli G.; Xie Z. Isolation, Stereochemical Study, and Racemization of (±)-pratenone A, the First Naturally Occurring 3-(1-naphthyl)-2-benzofuran-1(3H)-one Polyketide from a Marine-derived Actinobacterium. Chirality 2020, 32 (3), 299–307. 10.1002/chir.23178. 31975445

[ref4] Abdelfattah M. S.; Arai M. A.; Ishibashi M. Bioactive Secondary Metabolites with Unique Aromatic and Heterocyclic Structures Obtained from Terrestrial Actinomycetes Species. Chem. Pharm. Bull. (Tokyo) 2016, 64 (7), 668–675. 10.1248/cpb.c16-00038. 26936155

[ref5] Zheng Q.; Tian Z.; Liu W. Recent Advances in Understanding the Enzymatic Reactions of [4 + 2] Cycloaddition and Spiroketalization. Curr. Opin. Chem. Biol. 2016, 31, 95–102. 10.1016/j.cbpa.2016.01.020. 26870924

[ref6] Finefield J. M.; Sherman D. H.; Kreitman M.; Williams R. M. Enantiomeric Natural Products: Occurrence and Biogenesis. Angew. Chem., Int. Ed. 2012, 51 (20), 4802–4836. 10.1002/anie.201107204. PMC3498912 22555867

[ref7] Ikonnikova V. A.; Solyev P. N.; Terekhov S. S.; Alferova V. A.; Tyurin A. P.; Korshun V. A.; Baranov M. S.; Mikhaylov A. A. Total Synthesis of Elmenols A and B and Related Rearranged Angucyclinones. ChemistrySelect 2021, 6 (42), 11775–11778. 10.1002/slct.202103755.

[ref8] Wu C.; van der Heul H. U.; Melnik A. V.; Lübben J.; Dorrestein P. C.; Minnaard A. J.; Choi Y. H.; van Wezel G. P. Lugdunomycin, an Angucycline Derived Molecule with Unprecedented Chemical Architecture. Angew. Chem., Int. Ed. 2019, 58 (9), 2809–2814. 10.1002/anie.201814581. PMC6519343 30656821

[ref9] Mikhaylov A. A.; Ikonnikova V. A.; Solyev P. N. Disclosing Biosynthetic Connections and Functions of Atypical Angucyclinones with a Fragmented C-Ring. Nat. Prod. Rep. 2021, 38 (8), 1506–1517. 10.1039/D0NP00082E. 33480893

[ref10] Krasovskiy A.; Straub B. F.; Knochel P. Highly Efficient Reagents for Br/Mg Exchange. Angew. Chem., Int. Ed. 2006, 45 (1), 159–162. 10.1002/anie.200502220. 16307460

[ref11] Krasovskiy A.; Knochel P. A LiCl-Mediated Br/Mg Exchange Reaction for the Preparation of Functionalized Aryl- and Heteroarylmagnesium Compounds from Organic Bromides. Angew. Chem., Int. Ed. 2004, 43 (25), 3333–3336. 10.1002/anie.200454084. 15213967

[ref12] Clayden J.; Frampton C. S.; McCarthy C.; Westlund N. Perilithiation and the Synthesis of 8-Substituted-l-Naphthamides. Tetrahedron 1999, 55, 14161–14184. 10.1016/S0040-4020(99)00881-9.

[ref13] Clayden J.; Westlund N.; Wilson F. X. Diastereoisomeric Atropisomers of Peri-Substituted Naphthamides: Synthesis, Stereoselectivity and Stability. Tetrahedron Lett. 1999, 40 (45), 7883–7887. 10.1016/S0040-4039(99)01644-5.

[ref14] Su B.; Hartwig J. F. Irridium Catalyzed Silyl-Directed Peri-Borylation of C-H Bonds in Fused Polycyclic Arenes. Angew. Chem., Int. Ed. 2018, 57, 10163–10167. 10.1002/anie.201805086. PMC7484944 29779224

[ref15] Sato T.; Nogi K.; Yorimitsu H. Palladium Catalyzed Peri-Selective C-H Fluoroalkylation of Aryl Sulfoxides. ChemCatChem. 2020, 12, 3467–3471. 10.1002/cctc.202000485.

[ref16] Tan E.; Konovalov A. I.; Fernández G. A.; Dorel R.; Echavarren A. M. Ruthenium-Catalyzed *Peri* - and *Ortho* -Alkynylation with Bromoalkynes via Insertion and Elimination. Org. Lett. 2017, 19 (20), 5561–5564. 10.1021/acs.orglett.7b02655. 28976200 PMC5679662

[ref17] Berrou C.; Prévost S. Palladium-Catalyzed C8-Oxygenation of Naphthalene Derivatives: Direct Access to Naphtholactone Skeleton. Adv. Synth. Catal. 2021, 363, 4091–4095. 10.1002/adsc.202100317.

[ref18] Prévost S. Regioselective C-H Functionalization of Naphthalenes: Reactivity and Mechanistic Insights. ChemPlusChem. 2020, 85 (3), 476–486. 10.1002/cplu.202000005. 32187861

[ref19] Desai L. V.; Malik H. A.; Sanford M. S. Oxone as an Inexpensive, Safe, and Environmentally Benign Oxidant for C-H Bond Oxygenation. Org. Lett. 2006, 8 (6), 1141–1144. 10.1021/ol0530272. 16524288

[ref20] Neufeldt S. R.; Sanford M. S. *O* -Acetyl Oximes as Transformable Directing Groups for Pd-Catalyzed C-H Bond Functionalization. Org. Lett. 2010, 12 (3), 532–535. 10.1021/ol902720d. 20041702 PMC2830615

[ref21] Verga D.; Percivalle C.; Doria F.; Porta A.; Freccero M. Protecting Group Free Synthesis of 6-Substituted Naphthols and Binols. J. Org. Chem. 2011, 76 (7), 2319–2323. 10.1021/jo1025892. 21384814

[ref22] Garcıa O.; Nicolás E.; Albericio F. O-Formylation of Electron-Rich Phenols with Dichloromethyl Methyl Ether and TiCl4. Tetrahedron Lett. 2003, 44 (27), 4961–4963. 10.1016/S0040-4039(03)01168-7.

[ref23] Mahajan S. S.; Scian M.; Sripathy S.; Posakony J.; Lao U.; Loe T. K.; Leko V.; Thalhofer A.; Schuler A. D.; Bedalov A.; Simon J. A. Development of Pyrazolone and Isoxazol-5-One Cambinol Analogues as Sirtuin Inhibitors. J. Med. Chem. 2014, 57 (8), 3283–3294. 10.1021/jm4018064. 24697269 PMC4002067

[ref24] Wuts P. G. M. Greene’s Protective Groups in Organic Synthesis, 5th ed.; John Wiley & Sons Inc.: Hoboken, NJ, 2014.

[ref25] Dubost E.; Fossey C.; Cailly T.; Rault S.; Fabis F. Selective *Ortho* -Bromination of Substituted Benzaldoximes Using Pd-Catalyzed C-H Activation: Application to the Synthesis of Substituted 2-Bromobenzaldehydes. J. Org. Chem. 2011, 76 (15), 6414–6420. 10.1021/jo200853j. 21688782

[ref26] Curtin D. Y.; Grubbs E. J.; McCarty C. G. Uncatalyzed Syn-Anti Isomerization of Imines, Oxime Ethers, and Haloimines ^1^. J. Am. Chem. Soc. 1966, 88 (12), 2775–2786. 10.1021/ja00964a029.

[ref27] Jiang J.; Yuan D.; Ma C.; Song W.; Lin Y.; Hu L.; Zhang Y. Palladium-Catalyzed Regiospecific *Peri-* and *Ortho-* C-H Oxygenations of Polyaromatic Rings Mediated by Tunable Directing Groups. Org. Lett. 2021, 23 (2), 279–284. 10.1021/acs.orglett.0c03701. 33352055

[ref28] Asaad N.; Davies J. E.; Hodgson D. R. W.; Kirby A. J.; van Vliet L.; Ottavi L. The Search for Efficient Intramolecular Proton Transfer from Carbon: The Kinetically Silent Intramolecular General Base-Catalysed Elimination Reaction OfO-Phenyl 8-Dimethylamino-1-Naphthaldoximes. J. Phys. Org. Chem. 2005, 18 (2), 101–109. 10.1002/poc.858.

[ref29] Graafland T.; Wagenaar A.; Kirby A. J.; Engberts J. B. F. N. Structure and Reactivity in Intramolecular Catalysis. Catalysis of Sulfonamide Hydrolysis by the Neighboring Carboxyl Group. J. Am. Chem. Soc. 1979, 101 (23), 6981–6991. 10.1021/ja00517a034.

[ref30] Späth E. Über Ätherspaltung und Ersatz Von Alkoxyl gegen Alkyl mittels Organomagnesiumhaloide. Monatsh. Chem. 1914, 35 (3), 319–332. 10.1007/BF01519356.

[ref31] Newman M. S.; Sankaran V.; Olson D. R. Phenolic and Ketonic Tautomers in Polycyclic Aromatic Hydrocarbons. J. Am. Chem. Soc. 1976, 98 (11), 3237–3242. 10.1021/ja00427a031. 1262644

[ref32] Tsukamoto H.; Suzuki T.; Kondo Y. Remarkable Solvent Effect on Pd(0)-Catalyzed Deprotection of Allyl Ethers Using Barbituric Acid Derivatives: Application to Selective and Successive Removal of Allyl, Methallyl, and Prenyl Ethers. Synlett 2007, 2007 (20), 3131–3136. 10.1055/s-2007-992352.

[ref33] Brandsma L.; Verkruijsse H. D. Preparative Polar Organometallic Chemistry; Springer-Verlag: Berlin, 1987; Vol. 1.

[ref34] Hoogeboom J. Molecular Design, Synthesis and Evaluation of Chemical Biology Tools. Ph.D. Thesis, Wageningen University and Research, 2017.

[ref35] de Saint Aulaire P.; Hoogeboom J.; Uiterweerd M. T.; Zuilhof H.; Wennekes T. Synthetic Strategy towards a Carbocyclic N-Acetylneuraminic Acid. Eur. J. Org. Chem. 2022, 2022 (27), e202200297 10.1002/ejoc.202200297.

[ref36] Dethe D. H.; Murhade G. M.; Ghosh S. FeCl _3_ -Catalyzed Intramolecular Michael Reaction of Styrenes for the Synthesis of Highly Substituted Indenes. J. Org. Chem. 2015, 80 (16), 8367–8376. 10.1021/acs.joc.5b01071. 26182950

[ref37] Adams E. W.; Adkins H. Catalysis In Acetal Formation. J. Am. Chem. Soc. 1925, 47 (5), 1358–1367. 10.1021/ja01682a022.

