# Supplementary material for: Racemic Total Synthesis of Elmonin and Pratenone A, from Streptomyces, Using a Common Intermediate Prepared by peri-Directed C–H Functionalization
Source: Org Lett. 2022 Dec 19;24(51):9361–5. doi: 10.1021/acs.orglett.2c03449 (PMC9806855; doi:10.1021/acs.orglett.2c03449)
Supplement: Supplementary file 1 — ol2c03449_si_001.pdf [file ol2c03449_si_001.pdf]

Supporting information

Racemic Total Synthesis of elmonin and pratenone A, from *Streptomyces*, Using a Common Intermediate Prepared by *peri*-Directed C–H Functionalization

Michiel T. Uiterweerd and Adriaan J. Minnaard*

Rijksuniversiteit Groningen, Faculty of Science and Engineering, Stratingh Institute for Chemistry, Nijenborgh 7, 9747 AG, Groningen, The Netherlands.

*Email: a.j.minnaard@rug.nl

Contents

S1: Detailed experimental procedures ---**2**

Synthesis of naphthalene fragment ***9*** *--2*

Synthesis of anisole fragments ***12*** *and* ***8*** *---8*

Synthesis of elmonin ***1*** *-- 10*

Synthesis of pratenone A ***2*** *-- 12*

S2: ^{1}H-NMR and ^{13}C-NMR spectra --- **16**

S3: ESI HRMS spectra--- **54**

S1: Detailed experimental procedures

General

All moisture and oxygen sensitive reactions were executed under a N_2 atmosphere. Reactions above rt were heated using an IKA stir plate provided with an Asynt drysyn aluminium heating block. All reaction solvents were purchased from commercial vendors and used without further purification unless specified otherwise. Reagents were purchased from chemical vendors and used without further treatment or purification, unless stated otherwise. Anhydrous solvents were obtained using a solvent purification system unless stated otherwise. NMR spectra were recorded on an Agilent 400 NMR spectrometer, or on a Bruker 600 MHz NMR spectrometer in the solvent as described specifically for each compound. Detected ^{1}H-nuclei at 400 MHz or 600 MHz, ^{13}C-nuclei at 101 MHz or 151 MHz. Reported chemical shifts are given in ppm, relative to the residual solvent signal. The multiplicity descriptor bs = broad singlet. IR spectroscopic analyses were done using a Perkin-Elmer Spectrum Two UATR FT-IR spectrometer. Analytical TLC plates (60/Kieselguhr F_{254}, 0.25 mm), provided with a fluorescent marker, were obtained from Merck Chemicals. Spots were visualised by means of a UV lamp or appropriate standard staining solutions; $KMnO_4$, Anisaldehyde, Seebach's stain or phosphomolybdic acid. HRMS was executed on a Thermo-Fisher Orbitrap Electron Spray Ionization (ESI) mass spectrometer at positive ionization mode.

Synthesis of naphthalene fragment 9

Oxime ether 16

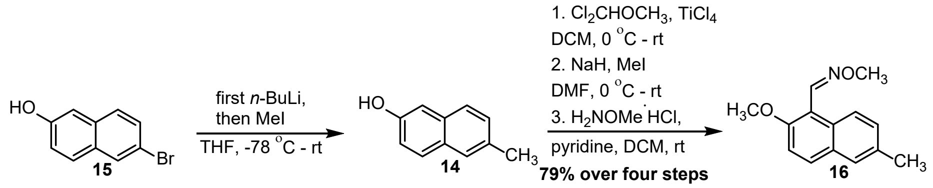

Following a modified literature procedure for scale up,[1] a dried 1 L three necked flask was provided with a two-way adapter, a mechanical overhead stirrer, a thermometer, a nitrogen inlet and a dropping funnel. The set-up was loaded with 6-bromonaphthalen-2-ol **15** (50.0 g, 224 mmol) which was dissolved in anhydrous THF (450 mL).[a] The flask was submerged into a dry-ice/acetone bath, cooling the contents to -78 °C. *n*-BuLi 2.5 M solution in hexane (200 mL, 500 mmol) was added dropwise through the dropping funnel, while maintaining the temperature between -78 °C and -65 °C.[b] A precipitate formed and the resulting viscous pale yellow suspension was stirred for two h while maintaining the temperature around -78 °C. Methyl iodide (62.0 mL, 141.4 g, 996 mmol) was added dropwise through the dropping funnel, while not allowing the temperature to exceed -50 °C. The cooling bath was removed and the mixture was allowed to heat to 0 °C. After 30 min the cold, clear solution was quenched by addition of water (100 mL), the mixture was acidified using 1 M HCl_{aq} and then extracted with ether (3 × 250 mL). The combined organic layers were washed with 10% $NaHSO_{3aq}$ (250 mL), dried over $MgSO_4$ and concentrated by rotary evaporation, providing the product **14** as a creamy white crystalline solid (35.89 g, quantitative), which was sufficiently pure to be used in the next step.[b]

^{1}H NMR (400 MHz, $CDCl_3$) δ 7.68 (d, *J* = 8.7 Hz, 1H), 7.60 (d, *J* = 8.4 Hz, 1H), 7.56 (s, 1H), 7.29 (dd, *J* = 8.4, 1.8 Hz, 1H), 7.11 (d, *J* = 2.5 Hz, 1H), 7.08 (dd, *J* = 8.7, 2.6 Hz, 1H), 5.11 (s, 1H), 2.50 (s, 3H).

^{13}C NMR (101 MHz, $CDCl_3$) δ 152.6, 133.1, 132.7, 129.2, 128.9, 126.8, 126.3, 117.7, 109.5, 21.5.

HRMS calcd for $C_{11}H_{10}O$ $\{M+H\}^+$: 159.0803, found: 159.0804.

Mp 110 – 114 °C.

Analytical data match with those reported in the literature.[1]

Notes:
[a]Anhydrous THF, free of inhibitor, was purchased from Merck Chemicals.

[b]One hour was required to add all the *n*-BuLi solution. An efficient and sufficiently large cooling bath is needed. The addition of *n*-BuLi was expedited by adding small portions of liquid nitrogen to the cooling bath from time to time.

[c]An analytically pure sample was obtained by crystallization from boiling toluene.

Following a modified literature procedure:[2] compound **14** (5.23 g, 33.1 mmol) and chloromethyl methylether (3.30 mL, 4.29 g, 37.4 mmol) were dissolved in dichloromethane (330 mL). The solution was cooled in an ice bath, then $TiCl_4$ (7.70 mL, 69.7 mmol) was added dropwise via a syringe. The ice bath was removed and the dark red solution was allowed to stir at rt overnight. The reaction mixture was quenched by the addition of 1 M HCl_{aq} (50 mL), the organic layer was separated and the aqueous layer was extracted with dichloromethane (2 × 50 mL). The combined organic layers were dried over $MgSO_4$, then filtered, a spoon of activated charcoal was added and the resulting suspension was stirred for five min, then filtered again and concentrated by means of rotary evaporation, providing the crude aldehyde (5.56 g) as a dark brown crystalline solid in sufficient purity.

^{1}H-NMR (400 MHz, $CDCl_3$) δ 10.75 (s, 1H), 8.21 (d, *J* = 8.6 Hz, 1H), 7.88 (d, *J* = 9.1 Hz, 1H), 7.55 (s, 1H), 7.43 (dd, *J* = 8.6, 1.9 Hz, 1H), 7.09 (d, *J* = 9.1 Hz, 1H), 2.49 (s, 3H).

^{13}C NMR (101 MHz, $CDCl_3$) δ 193.3, 164.3, 138.6, 134.1, 131.1, 130.9, 128.7, 128.1, 119.1, 118.5, 111.3, 21.1.

HRMS calcd for $C_{12}H_{10}O_2$ $\{M+H\}^+$: 187.0754, found: 187.0753.

Mp 91 – 92 °C.

Next, the crude aldehyde (5.56 g, 29.9 mmol) was dissolved in dry DMF (30 mL). Sodium hydride (60% suspension in mineral oil, 1.44 g, 36.1 mmol) was pre-washed with pentane (three times) and suspended in DMF (20 mL) and cooled in an ice bath. The solution of the aldehyde was added dropwise via a syringe to the sodium hydride at such a rate that the evolution of H_2 gas was under control. After 30 min the evolution of H_2 gas had ceased, and methyl iodide (2.20 mL, 5.09 g, 35.8 mmol) was added dropwise via a syringe. The mixture was stirred for five h and then poured into brine (200 mL) which was acidified to pH ~ 1 with 1 M HCl_{aq}. The mixture was extracted with EtOAc (3 × 75 mL) and the combined organic layers were washed with brine (4 × 100 mL) and finally with 1 M $NaOH_{aq}$ (30 mL), dried over $MgSO_4$ and concentrated, providing the crude methyl ether (5.69 g) as a dark brown colored crystalline solid which was sufficiently pure to be used in the next step.

^{1}H-NMR (400 MHz, $CDCl_3$) δ 10.87 (s, 1H), 9.17 (d, *J* = 8.8 Hz, 1H), 7.95 (d, *J* = 9.1 Hz, 1H), 7.52 (s, 1H), 7.45 (dd, *J* = 8.9, 1.9 Hz, 1H), 7.23 (d, *J* = 9.1 Hz, 1H), 4.01 (s, 3H), 2.47 (s, 3H).

^{13}C-NMR (101 MHz, $CDCl_3$) δ 192.0, 163.4, 136.9, 134.3, 132.1, 129.6, 128.8, 127.2, 124.8, 116.6, 112.5,

56.5, 21.2.

HRMS calcd for $C_{13}H_{12}O_2$ {M+H}$^+$: 201.0910, found: 201.0908.

Mp 51 – 55 °C.

Using a modified literature procedure,[3] the crude methyl ether (5.68 g, 28.4 mmol) was added to a suspension of $H_2NOMe{\cdot}HCl$ (2.84 g, 34.0 mmol) in DCM (100 mL). Pyridine (10 mL, 9.80 g, 124 mmol) was added and the mixture was stirred for 45 min. The reaction mixture was poured into 1 M HCl_{aq} (150 mL) and extracted with DCM (3 × 50 mL). The combined organic layers were washed with brine (100 mL) and dried over $MgSO_4$, concentration by means of rotary evaporation yielded a brown oil which was purified by means of column chromatography (ether/pentane, gradient 2.5:97.5, 10:90 and 20:80) affording the compound **16** (5.99 g, 26.2 mmol, 79% over four steps) as a single oxime isomer, being a slightly green oil.

^{1}H-NMR (400 MHz, $CDCl_3$) δ 8.85 (d, *J* = 8.8 Hz, 1H), 8.83 (s, 1H), 7.77 (d, *J* = 9.0 Hz, 1H), 7.55 (s, 1H), 7.40 (dd, *J* = 8.8, 1.9 Hz, 1H), 7.21 (d, *J* = 9.0 Hz, 1H), 4.09 (d, *J* = 0.8 Hz, 4H), 3.94 (d, *J* = 0.8 Hz, 3H), 2.49 (s, 3H).

^{13}C-NMR (101 MHz, $CDCl_3$) δ 156.5, 146.4, 133.5, 131.3, 130.2, 129.6, 129.4, 127.2, 125.9, 113.5, 112.8, 62.0, 56.6, 21.3.

HRMS calcd for $C_{14}H_{15}NO_2$ {M+H}$^+$: 230.1176, found, 230.1175.

Nitrile 18

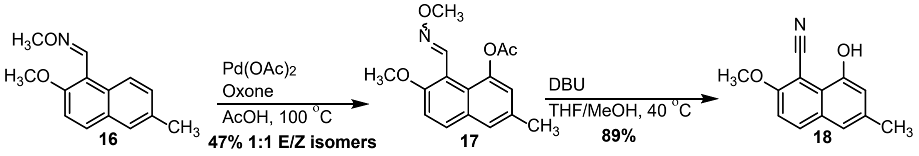

Using a modified procedure by Sanford *et al.*,[4] the reaction was performed in duplicates, in two 1 L round bottom flasks. In each flask were placed, a stir bar, oxime ether **16** (14.14 g, 61.7 mmol), $Pd(OAc)_2$ (692 mg, 3.08 mmol, 5 mol%) and oxone (37.9 g, 61.7 mmol). The contents were suspended in acetic acid (380 mL). The reaction mixture was stirred overnight at 100 °C. Each flask was poured into 1 L of ether and filtered over a celite plug to remove salts and palladium. The filtrates were combined and concentrated by means of rotary evaporation. The residue was co-evaporated with toluene one time to remove residual acetic acid, and loaded onto silica for purification by means of column chromatography (gradient, 10% and 20% EtOAc in pentane). This afforded the acetylated product **17** (16.76 g, 58.3 mmol, 47%) as an inseparable 1:1 isomeric* mixture being a bright orange coloured sticky that turned into an orange waxy solid upon standing.

^{1}H NMR (400 MHz, D_3COD) δ 8.47 (s, 1H, -C<u>H</u>=N), 7.87 – 7.78 (m, 2H), 7.76 (s, 1H, -C<u>H</u>=N), 7.51 – 7.45 (m, 2H) 7.40 – 7.28 (m, 2H), 7.02 – 6.98 (m, 2H), 3.93 – 3.85 (m, 9H), 3.71 (s, 3H), 2.45 – 2.40 (m, 6H), 2.35 – 2.29 (m, 6H).

^{13}C NMR (101 MHz, D_3COD) δ (170.0, 169.8), (156.0, 154.3), (146.9, 145.5), (145.2, 145.1), (133.4, 133.0), (130.8, 130.7), (130.6, 130.2), (125.3, 125.2), (124.4, 123.5), (123.0, 122.5), (113.8, 113.5), (111.23, 111.19), (60.5, 60.3), (55.9, 55.7), (20.0, 19.9), (19.7, 19.6).

HRMS calcd for $C_{16}H_{17}NO_4$ $\{M+H\}^+$: 288.1230, found: 288.1231.

Note:
*Full characterization of the two isomers by means of NMR was not possible. Some signals in the ^{1}H-NMR spectrum are indistinguishable from each other. In the ^{13}C-NMR spectrum, 16 sets of peaks could be identified, it was however not possible to assign the different peaks to the different isomers.

Optimisation of the conversion of **17** to **18**.

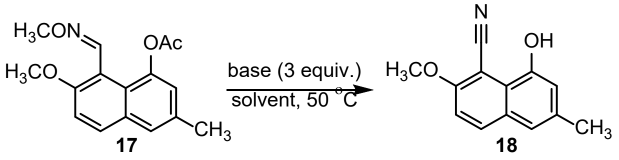

Entry	Base	Solvent	t [h]	yield[a]	Comment
1	K_2CO_3	MeOH	18	21%	-
2	Et_3N	MeOH	6	n.d.	degradation[b]
3	$i Pr_2EtN$	MeOH	6	n.d.	degradation[b]
4	DBU	MeOH	18	57%	-
5	DBU	THF	18	51%	-
6	DBU	EtOH	18	31%	-
7[c]	DBU	THF/MeOH (10:1)	18	86%	-

[a]Isolated yield from 25 mg starting material after column chromatography in a single attempt. [b]Conclusion based on inspection by TLC, which revealed highly polar base-line material. [c]Reaction performed at 400 mg (1.88 mmol).

Table 1: Optimization of the conversion of *peri*-naphthol ester **17** to form *peri*-cyano-naphthol **18**.

Typical procedure at large scale:

Using the optimised conditions, compound **17** (16.8 g, 58.3 mmol) was dissolved in a THF/MeOH mixture (10:1, 45 mL). DBU (26.8 mL, 27.3 g, 180 mmol) was added, whereupon the mixture turned black, and was heated to 55 °C for 68 h. The mixture was poured into 1 M HCl_{aq} (200 mL) and extracted using EtOAc (5 × 100 mL). The combined organic layers were washed with brine and concentrated by means of rotary evaporation. The resulting black mass was loaded onto silica for purification by column chromatography (EtOAc/pentane, gradient 40:60 and 50:50), affording nitrile **18** (11.1 g, 52.4 mmol, 89% yield) as a pale yellow crystalline solid.

^{1}H-NMR (400 MHz, $(CD_3)_2SO$) δ 10.36 (s, 1H, H-14), 8.02 (d, J = 9.2 Hz, 1H, H-2), 7.47 (d, J = 9.2 Hz, 1H, H-3), 7.17 (s, 1H, H-7), 6.78 (d, J = 1.7 Hz, 1H, H-9), 3.99 (s, 3H, H-12), 2.33 (s, 3H, H-12).

^{13}C-NMR (101 MHz, $(CD_3)_2SO$) δ 162.3, 151.9, 135.2, 134.9, 130.2, 121.8, 119.0, 117.0, 113.9, 113.6, 91.3, 57.2, 21.5.

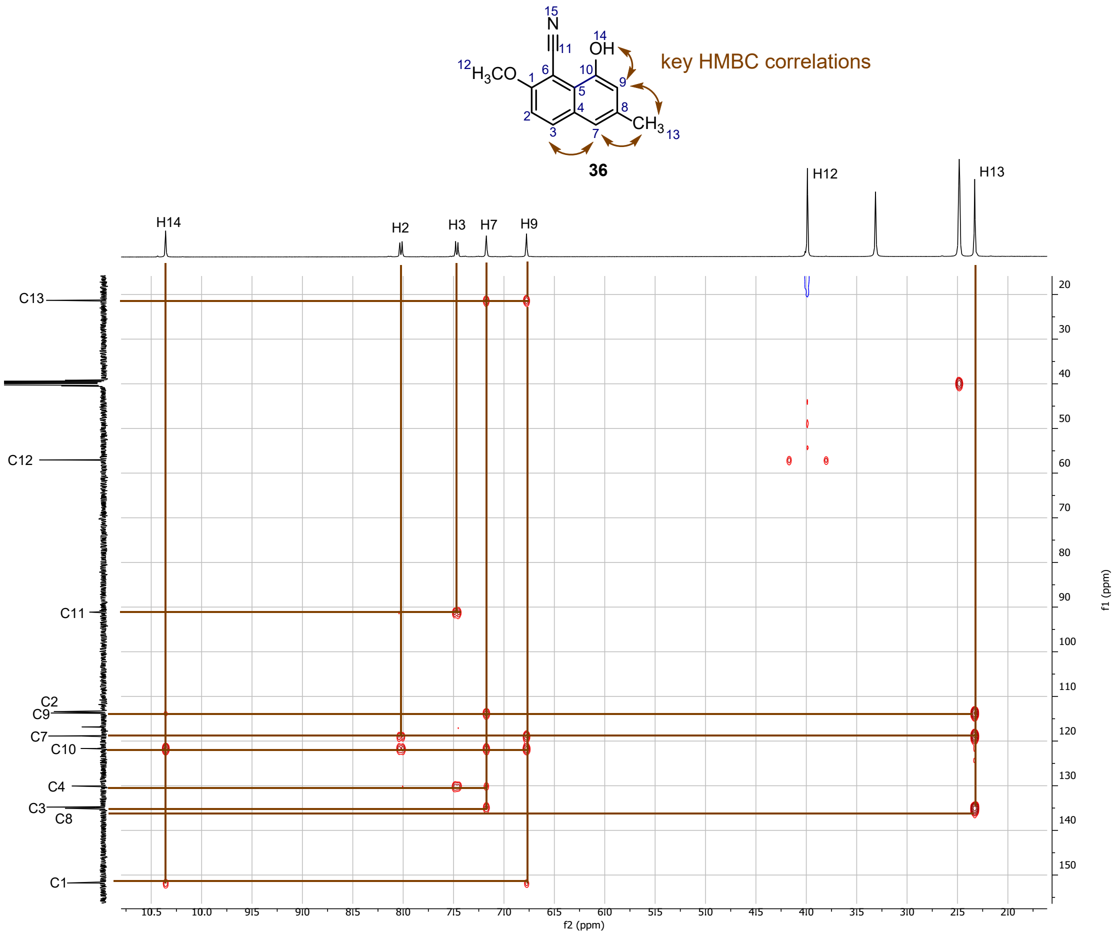

IR(neat) ν 3308 (-OH), 2218 (-CN) cm^{-1}.

HRMS calcd for $C_{13}H_{11}NO_2$ $\{M+H\}^+$: 214.0863, found: 214.0861.

Mp 168 – 172 °C.

Naphthalene fragment 9

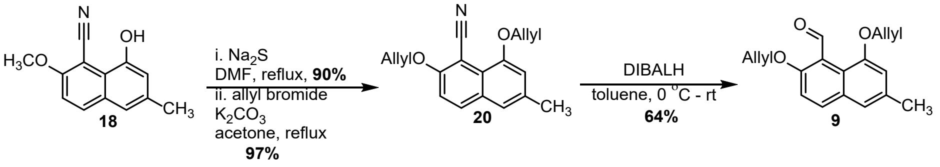

Compound **18** (1.92 g, 8.99 mmol) and anhydrous[a] Na_2S (841 mg, 10.8 mmol) were dissolved in dry DMF (36 mL). The mixture was heated to reflux[b] for 4 h and then cooled to room temperature. The mixture was diluted with water (100 mL), carefully acidified[c] using 1 M HCl_{aq} and extracted with EtOAc (4 × 75 mL), the combined organic layers were washed with brine (4 × 100 mL) and then dried over $MgSO_4$. The residue was loaded onto silica for purification by means of column chromatography (EtOAc/pentane, gradient 30:70 and 50:50), which afforded the *bis*-naphthol **19** (1.70 g, 8.13 mmol, 90% yield) as a pale yellow crystalline solid.

^{1}H-NMR (400 MHz, MeOD) δ 7.74 (dd, J = 9.1, 1.4 Hz, 1H), 7.08 (dd, 9.0, 1.5 Hz, 1H), 7.05 (s, 1H), 6.75

(s, 1H), 2.35 (s, 3H).

^{13}C-NMR (101 MHz, MeOD) δ 161.3, 151.4, 134.5, 134.1, 129.7, 121.6, 118.8, 117.4, 116.6, 113.1, 88.4, 19.9.

IR(neat) ν 3464 (–OH), 3395 (–OH), 3243 (–OH), 2210 (–CN) cm^{-1}.

HRMS calcd for $C_{12}H_9NO_2$ {M+H}$^+$: 200.0706, found: 200.0706.

Mp 205 – 208 °C.

Notes:
[a]It is essential to use fully anhydrous sodium sulphide. In one case, accidental use of hydrated sodium sulphide, due to erroneous labelling of the bottle by a chemical vendor, led to a poor yield.

[b]This reaction was performed in a well-ventilated hood because of the formation of copious amounts of foul-smelling $(H_3C)_2S$ and H_3CSH.

[c]It was observed that proper acidification to pH ~ 1 is essential for complete extraction of all the material. Residual sulphide might be converted to toxic H_2S gas, therefore aqueous waste should be kept in a fume hood and basified before disposal. Exposure to any H_2S should be avoided during the extraction procedure as much as possible.

The *bis*-naphthol **19** (1.70 g, 8.53 mmol), K_2CO_3 (4.13 g, 29.9 mmol) and allyl bromide (2.40 mL, 3.36 g, 27.7 mmol) were suspended in acetone. The resulting turbid mixture was heated to reflux for 20 h and then allowed to cool to room temperature. The reaction mixture was diluted with water (100 ml) and extracted with EtOAc (3 × 100 mL), the combined organic layers were washed with brine (30 mL) and dried over $MgSO_4$. The resulting brown solid was loaded onto silica for purification by means of column chromatography (gradient, EtOAc/pentane 10:90, 15:85 and 20:80), affording compound **20** (2.32 g, 8.29 mmol, 97%) as a pale yellow crystalline solid.

^{1}H-NMR (400 MHz, $CDCl_3$) δ 7.81 (d, J = 9.1 Hz, 1H), 7.18 (d, J = 9.2 Hz, 1H), 7.15 (s, 1H), 6.78 (s, 1H), 6.27 (ddt, J = 15.8, 10.6, 5.4 Hz, 1H), 6.08 (ddt, J = 16.7, 10.2, 4.9 Hz, 1H), 5.56 – 5.46 (m, 2H), 5.39 – 5.23 (m, 2H), 4.84 – 4.68 (m, 4H), 2.44 (s, 3H).

^{13}C-NMR (101 MHz, $CDCl_3$) δ 161.3, 152.9, 135.1, 133.7, 132.9, 132.3, 129.9, 123.3, 120.2, 120.1, 118.1, 118.0, 116.7, 113.9, 111.3, 70.4, 70.2, 21.7.

HRMS calcd for $C_{18}H_{17}NO_2$ {M+H}$^+$: 280.1332, found: 280.1333.

Mp 73 – 77 °C.

Compound **20** (2.32 g, 8.30 mmol) was dissolved in toluene (64 mL). The mixture was cooled in an ice bath and a 1 M solution of DIBALH in toluene (10 mL, 10.0 mmol) was added dropwise to the reaction mixture. The solution was stirred for 2 h, then the ice bath was removed and stirring was continued for 24 h. The reaction mixture was added to a saturated solution of Rochelle's salt, and the mixture was left to stand overnight with vigorous stirring. The mixture was extracted with EtOAc (3 × 75 mL), the combined organic layers were washed with brine (20 mL) and dried with $MgSO_4$. The residue was loaded onto silica

for purification by means of column chromatography (EtOAc/pentane, gradient 12:88 and 25:75), which afforded recovered starting material (207 mg, 0.741 mmol, 9%) and fragment **9** (1.50 g, 5.30 mmol, 64%) as a dark green crystalline solid.

^{1}H NMR (400 MHz, $CDCl_3$) δ 10.77 (s, 1H), 7.71 (d, J = 9.0 Hz, 1H), 7.21 (d, J = 9.0 Hz, 1H), 7.17 (s, 1H), 6.70 (d, J = 1.4 Hz, 1H), 6.16 – 5.95 (m, 2H), 5.46 – 5.37 (m, 2H), 5.35 – 5.22 (m, 2H), 4.72 – 4.47 (m, 4H), 2.44 (d, J = 1.0 Hz, 3H).

^{13}C-NMR (101 MHz, $CDCl_3$) δ 194.8, 153.6, 153.0, 134.4, 133.1, 132.6, 130.9, 130.5, 122.9, 122.3, 120.1, 118.1, 117.6, 116.1, 110.1, 71.0, 70.2, 21.7.

IR(neat) ν 3082 (Ar C–H), 2918 (Alk. C–H), 1701 (HC=O), 1265 cm^{-1}.

HRMS calc for $C_{18}H_{18}O_3$ $\{M+Na\}^+$: 305.1148, found: 305.1152.

Mp 45 – 51 °C.

Synthesis of anisole fragments 12 and 8

Anisole fragment 12

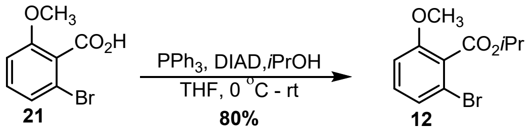

2-bromo-6-methoxybenzoic acid **21** (3.00 g, 13.0 mmol), PPh_3 (3.40 g, 13.0 mmol) and *i*PrOH (3.00 mL, 2.36 g, 39.2 mmol) were dissolved in THF (90 mL). The solution was cooled in an ice bath, DIAD (2.60 mL, 2.65 g, 13.1 mmol) was added dropwise via syringe. The ice bath was removed and the solution was stirred at rt for 1 h, then the reaction mixture was concentrated by means of rotary evaporation. The yellow oily residue was triturated with EtOAc/pentane (1:99), giving a biphasic mixture. After vigorous swirling, white crystals of TPPO separated from the mixture*, the suspension was passed through a glass filter, the residue was washed with EtOAc/pentane (1:99) and the combined filtrates were concentrated by rotary evaporation, affording a yellow oil which was purified by column chromatography (EtOAc/pentane, 20:80), affording the title compound **12** (2.84 g, 10.4 mmol, 80% yield) as a colourless oil.

^{1}H-NMR (400 MHz, $CDCl_3$) δ 7.19 (t, J = 8.10 Hz, 1H), 7.13 (dd, J = 8.06, 1.11 Hz, 1H), 6.86 (dd, J = 8.1, 1.1 Hz, 1H), 5.32 (hept., J = 6.3 Hz, 1H), 3.82 (s, 3H), 1.39 (s, 3H), 1.37 (s, 3H).

^{13}C-NMR (101 MHz, $CDCl_3$) δ 165.7, 157.1, 131.0, 126.5, 124.5, 119.6, 110.0, 69.5, 56.2, 21.7.

HRMS calcd for $C_{11}H_{13}BrO_3$ $\{M+H\}^+$: 273.0121, found: 273.0122.

Mp 41 – 43 °C.

Note:
*In some instances, prolonged swirling and repeated heating to 45 °C, and cooling back to room temperature was required to stimulate crystallization of the phosphine oxide by-product.

Anisole fragment 8

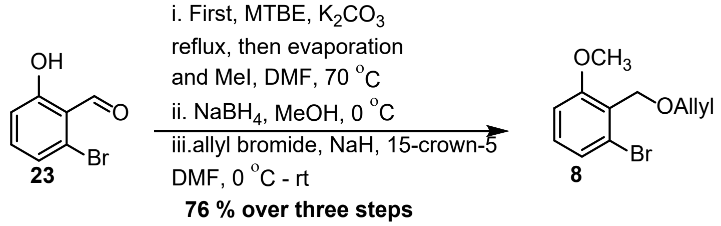

2-bromo-6-hydroxybenzaldehyde **23** (15.0 g, 74.6 mmol) and K_2CO_3 (30.9 g, 224 mmol) were suspended in MTBE (150 mL) and the mixture was heated to reflux overnight. The resulting intensely yellow suspension was concentrated by means of rotary evaporation. The mixture was re-suspended in DMF (150 mL), MeI (31.8 g, 224 mmol) was added and the mixture was heated to 70 °C overnight. The mixture was concentrated by means of rotary evaporation, the residue was triturated by addition of 100 mL of water. The suspension was poured over a Büchner funnel, the residue was washed with water (3 × 50 mL) and dried on the filter for one h. The solid was transferred to a 250 mL round-bottom flask and further dried to a constant weight by using a rotary evaporator at 45 °C and full vacuum. The methyl ether (15.3 g, 224 mmol, quantitative) was obtained as a yellow crystalline solid, which sufficiently pure to be used in the next step.

^{1}H-NMR (400 MHz,$CDCl_3$) δ 10.39 (s, 1H), 7.31 (t, J = 8.2 Hz, 1H), 7.22 (dd, J = 8.1, 1.0 Hz, 1H), 6.94 (dd, J = 8.3, 1.0 Hz, 1H), 3.90 (s, 4H).

^{13}C-NMR (101 MHz, $CDCl_3$) δ 190.4, 161.9, 134.8, 126.5, 124.8, 123.4, 111.0, 56.2.

Mp 53 – 55 °C.

NMR data are in agreement with those reported earlier in the literature.[3]

The crude methyl ether was dissolved in MeOH (150 mL) and cooled in an ice bath. Sodium borohydride (4.06 g, 107 mmol) was added in small portions in such a rate to control hydrogen evolution, the mixture was stirred for 30 min, and the ice bath was removed, the mixture was diluted with water (60 mL) and 1 M HCl_{aq} (10 mL), and then extracted with Et_2O (3 × 25 mL). The combined organic layers were washed with brine (25 mL) and dried over $MgSO_4$, concentration by means of rotary evaporation provided the crude alcohol (15.0 g) as a pale red oil that solidified upon standing. The crude but essentially pure alcohol was used immediately in the next step.

^{1}H-NMR (400 MHz, $CDCl_3$) δ 7.18 (dd, J = 8.1, 1.3 Hz, 1H), 7.12 (t, J = 8.1 Hz, 1H), 6.85 (dd, J = 8.0, 1.2 Hz, 1H), 4.88 (s, 2H), 3.87 (s, 3H), 2.20 (bs, 1H).

^{13}C-NMR (101 MHz,$CDCl_3$) δ 158.8, 129.9, 128.5, 125.1, 125.0, 109.9, 60.2, 55.9.

Mp 64 – 65 °C.

The analytical data are in agreement with those reported previously in the literature.[5]

A 1 L round bottom flask was provided with a pressure equalizing dropping funnel, a nitrogen inlet and a stir bar. Sodium hydride (60% in mineral oil, 4.33 g, 108 mmol), was added, pre-washed (three times) with small portions of pentane and then subsequently suspended in DMF (50 mL). The flask was cooled in an ice bath, the crude alcohol (15.0 g, 69.0 mmol) was dissolved in DMF (100 mL) and added dropwise via a

dropping funnel to the sodium hydride suspension, while stirring. 15-crown-5 (20.0 mL, 22.3 g, 101 mmol) was added (note: hydrogen gas formation), then allyl bromide (8.00 mL, 11.2 g, 92.5 mmol) was added dropwise to the reaction mixture and the ice bath was removed*. The solution was stirred at rt overnight, and quenched by pouring it into 1M HCl_{aq} (300 mL). The mixture was extracted using Et_2O (3 × 200 mL), the combined organic layers were washed with brine (5 × 200 mL) dried using $MgSO_4$ and concentrated by means of rotary evaporation. The residue was purified by column chromatography (EtOAc/pentane, 5:95), which yielded fragment **8** (14.7 g, 57.0 mmol, 67% over three steps) as a colourless oil.

^{1}H-NMR (400 MHz, $CDCl_3$) δ 7.18 (dd, *J* = 8.0, 1.2 Hz, 1H), 7.12 (t, *J* = 8.1 Hz, 1H), 6.83 (dd, *J* = 8.1, 1.2 Hz, 1H), 5.98 (ddt, *J* = 17.3, 10.3, 5.7 Hz, 1H), 5.32 (dq, *J* = 17.2, 1.7 Hz, 1H), 5.19 (dq, *J* = 10.3, 1.3 Hz, 1H), 4.69 (s, 2H), 4.08 (dt, *J* = 5.8, 1.4 Hz, 2H), 3.84 (s, 3H).

^{13}C-NMR (101 MHz, $CDCl_3$) δ 159.3, 135.2, 130.2, 127.0, 126.1, 125.1, 117.0, 110.0, 71.6, 65.7, 56.1.

HRMS calcd for $C_{11}H_{13}BrO_2$ $\{M+H\}^+$: 257.0172, found: 257.0171.

Note:
*The addition of the crown ether and allyl bromide induced sudden hydrogen gas formation, the addition steps should be performed with care.

Synthesis of elmonin 1

Benzhydrol 7

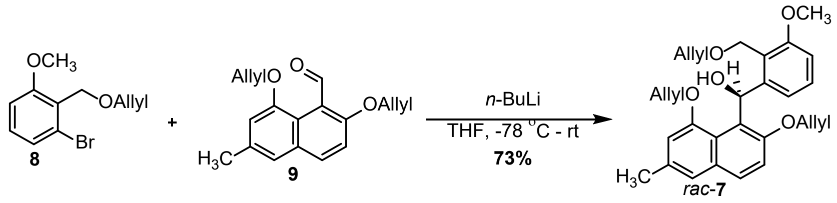

A Schlenk flask was provided with a solution of anisole fragment **8** (309 mg, 1.20 mmol) in THF (4.8 mL) and TMEDA (0.17 mL, 134 mg, 1.36 mmol). The solution was cooled to -78 °C, and 1.6 M *n*-BuLi solution in hexanes (0.72 mL, 74 mg, 1.16 mmol) was added dropwise through a syringe. The solution was stirred for 1.5 h and progress of the lithiation was probed by GC-MS.* Fragment **9** (261 mg, 0.924 mmol) was dissolved in THF (4.5 mL) and added dropwise via a syringe to the aryl lithium solution, the cooling bath was removed and the solution was stirred at rt for 1 h. The mixture was quenched by addition of 1 M NH_4Cl_{aq} (10 mL), the mixture was diluted with EtOAc (10 mL) and further neutralized by dropwise addition of 1 M HCl_{aq}. The organic layer was separated and the aqueous layer was extracted with EtOAc (2 × 25 mL), the combined organic layers were washed with brine (20 mL), dried over $MgSO_4$ and concentrated. The crude product was purified by column chromatography (EtOAc/Pentane, 10:90), providing the racemic benzhydrol **7** (330 mg, 0.682 mmol, 73% yield) as a pale yellow gummy solid.

^{1}H-NMR (400 MHz, $CDCl_3$) δ 7.68 (d, *J* = 9.0 Hz, 1H), 7.40 (d, *J* = 11.4 Hz, 1H), 7.27 – 7.16 (m, 2H), 6.98 (t, *J* = 8.0 Hz, 1H), 6.78 (d, *J* = 8.1 Hz, 1H), 6.69 (d, *J* = 1.6 Hz, 1H), 6.55 (d, *J* = 7.8 Hz, 1H), 6.10 – 5.84 (m, 2H), 5.44 – 5.24 (m, 3H), 5.20 – 5.02 (m, 6H), 4.82 (d, *J* = 9.9 Hz, 1H), 4.62 – 4.49 (m, 2H), 4.45 (dd, *J* = 12.4, 6.0 Hz, 1H), 4.24 (dd, *J* = 12.4, 5.3 Hz, 1H), 4.10 (d, *J* = 5.7 Hz, 2H), 3.86 (s, 3H), 2.43 (s, 3H).

^{13}C-NMR (101 MHz, $CDCl_3$) δ 159.0, 154.2, 153.9, 145.7, 135.9, 133.5, 133.3, 132.5, 132.3, 129.0, 127.7, 125.7, 125.0, 123.6, 121.5, 120.6, 117.5, 116.3, 116.2, 111.1, 109.7, 71.9, 71.0, 70.2, 68.9, 63.3, 56.1, 21.4.

HRMS calcd for $C_{29}H_{32}O_5$ $\{M+Na\}^+$: 483.2142, found: 483.2152.

Note:
*A 0.1 mL aliquot of the reaction mixture was injected in 1 mL of MeOH and shaken for ten s. The solvent was evaporated and the residue triturated in 1 mL of EtOAc which was analysed by GC-MS to monitor the disappearance of the starting material (the bromide isotopes were diagnostic).

Benzophenone 24

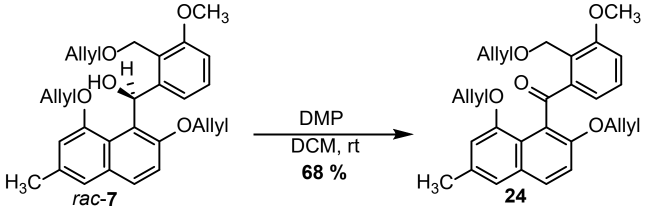

Benzhydrol **7** (300 mg, 0.651 mmol) was dissolved in CH_2Cl_2 (5 mL), Dess-Martin periodinane (414 mg, 0.977 mmol) was added in a single portion. The solution was stirred for 16 h. The reaction mixture was quenched by addition of 5% $Na_2S_2O_{3aq}$ (50 mL), the solution was stirred for 10 min and then extracted with EtOAc (3 × 25 mL). The combined organic layers were washed with brine (15 mL), dried over $MgSO_4$ and concentrated by means of rotary evaporation. The residue was loaded onto celite for purification by means of column chromatography (EtOAc/pentane 10:90), providing benzophenone **24** (204 mg, 0.445 mmol, 68% yield) as a pale yellow oil which turned into a gummy solid on standing.

^{1}H-NMR (400 MHz, $CDCl_3$) δ 7.70 (d, J = 9.0 Hz, 1H), 7.21 – 7.17 (m, 2H), 7.16 – 7.07 (m, 2H), 7.00 (dd, J = 7.9, 1.6 Hz, 1H), 6.59 (s, 1H), 6.11 – 5.97 (m, 1H), 5.86 – 5.72 (m, 1H), 5.64 – 5.52 (m, 1H), 5.32 (d, J = 17.3, 1.6 Hz, 1H), 5.21 – 4.92 (m, 7H), 4.61 – 4.31 (m, 4H), 4.19 (d, 2H), 3.89 (s, 3H), 2.42 (s, 3H).

^{13}C-NMR (101 MHz, $CDCl_3$) δ 197.8, 159.3, 153.2, 152.8, 139.6, 136.3, 134.2, 133.4, 132.8, 130.8, 129.3, 128.4, 126.8, 124.9, 122.5, 120.0, 117.5, 117.3, 116.4, 116.1, 114.4, 109.9, 72.0, 70.8, 69.7, 62.6, 56.4, 21.9, 14.3.

HRMS calcd for $C_{29}H_{30}O_5$ $\{M+Na\}^+$: 481.1986, found: 481.1992.

Elmonin 1

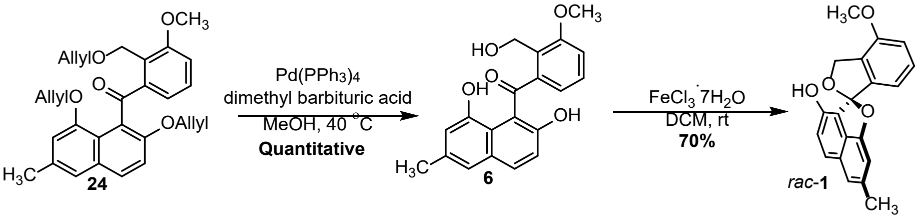

A 100 mL round-bottom flask was provided with a stir bar, $Pd(PPh_3)_4$ (435 mg, 0.375 mmol), dimethyl barbituric acid (1.76 g, 11.3 mmol) and a solution of compound **24** (1.73 g, 3.72 mmol) in absolute methanol. The mixture was heated for 3.5 h at 40 °C. The mixture was allowed to cool to room temperature and concentrated by means of rotary evaporation. The residue was redissolved in EtOAc (50 mL) and washed with water (50 mL) The organic layer was separated and the aqueous layer was extracted with EtOAc (2 × 75 mL). The combined organics were dried over $MgSO_4$. The material was loaded on silica and the compound was separated* on a column using 50% EtOAc in pentane, to provide compound **6** (1.57

g, quantitative) as a yellow glassy foam.

^{1}H-NMR (600 MHz, D_3COD) δ 7.67 (d, *J* = 8.9 Hz, 1H), 7.23 – 7.13 (m, 2H), 7.12 – 7.07 (m, 2H), 6.92 (dd, *J* = 6.5, 2.4 Hz, 1H), 6.51 (d, *J* = 1.6 Hz, 1H), 4.97 (s, 2H), 3.90 (s, 3H), 2.34 (s, 3H).

^{13}C-NMR (151 MHz, D_3COD) δ 204.1, 159.4, 152.7, 152.6, 142.3, 135.0, 131.5, 129.8, 122.7, 120.5, 56.62, 56.58, 21.5.

HRMS calcd for $C_{20}H_{18}O_5$ $\{M+Na\}^+$: 361.1046, found: 361.1047.

Note:
*In some instances, elmonin was isolated as a side product of the reaction, but this result is not reproducible.

A 250 round bottom flask was fitted with compound **6** (900 mg, 2.66 mmol), a stir bar and DCM. To the yellow solution was added $FeCl_3·6H_2O$. The reaction mixture turned dark red/purple. After 1.5 h of stirring at rt, the reaction mixture was washed with water (50 mL) and the aq. layer was extracted with DCM (2 × 40 mL). The combined organic layers were dried over $MgSO_4$ and concentrated by means of rotary evaporation. The material was loaded onto silica and chromatographed using 25% EtOAc in pentane, affording elmonin **1** (596 mg, 1.86 mmol, 70%) as a pale yellow glassy foam.

^{1}H-NMR (400 MHz, $(CD_3)_2CO$) δ 8.55 (bs, 1H, OH), 7.66 (d, *J* = 8.6 Hz, 1H), 7.31 (t, *J* = 7.8 Hz, 2H), 7.15 (d, *J* = 8.6 Hz, 1H), 7.09 (s, 1H), 7.05 (d, *J* = 8.1 Hz, 1H), 6.60 (d, *J* = 7.6 Hz, 1H), 6.46 (s, 1H), 5.32 (d, *J* = 12.9 Hz, 1H), 5.23 (d, *J* = 12.9 Hz, 1H), 3.94 (s, 4H), 2.44 (s, 3H).

^{13}C-NMR (101 MHz, $(CD_3)_2CO$) δ 157.1, 155.0, 150.6, 140.5, 137.0, 130.9, 128.9, 128.5, 128.3, 126.4, 123.9, 122.0, 117.9, 115.8, 115.3, 111.9, 103.9, 71.9, 55.9, 22.7.

HRMS calcd for $C_{20}H_{16}O_4$ $\{M+H\}^+$: 321.1121, found: 321.1124.

The analytical data are in agreement with those reported for natural elmonin.[6,7]

Synthesis of pratenone A 2

Lactone 11

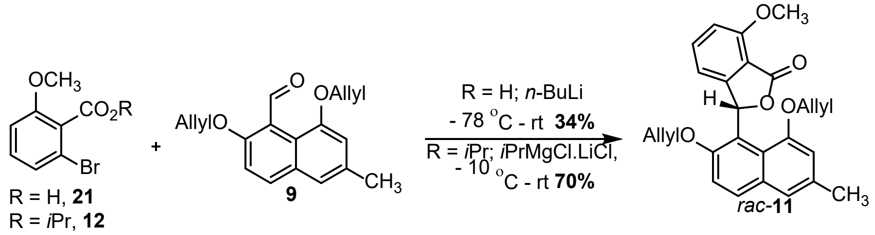

method 1, via lithium-bromo exchange:

2-bromo-6-methoxybenzoic acid **21** (123 mg, 0.531 mmol) was dissolved in THF (3 mL) and transferred to a 10 mL dried Schlenk flask, which was subsequently cooled to -78 °C. 1.6 M *n*-BuLi solution in hexane (0.67 mL, 1.07 mmol) was added dropwise via a syringe, and the solution was stirred for 15 min. Compound **9** (150 mg, 0.531 mmol) was dissolved in THF (3 mL) and added dropwise via a syringe to the reaction mixture. The cooling bath was removed and the reaction mixture was allowed to warm to room temperature. The mixture was quenched by adding 1 M HCl_{aq} (20 mL) and was then extracted with EtOAc (3 × 20 mL),

the combined organic layers were washed with brine (20 mL), dried over $MgSO_4$ and concentrated by means of rotary evaporation. The crude residue was loaded onto silica for purification by means of column chromatography (EtOAc/pentane, gradient, 20:80 and 50:50), to provide lactone **11** (76 mg, 0.182 mmol, 34%) as a pale yellow crystalline solid.

method 2, by bromo-magnesium exchange:

According a modified literature procedure by Krasovsky and Knochel,[8] a dried Schlenk flask was provided with a stir bar and 1.3 M solution of *i*PrMgCl.LiCl in THF (1.0 mL, 1.30 mmol). 15-crown-5 (0.260 mL, 289 mg, 1.31 mmol) was added dropwise via syringe, whereupon a white precipitate and a slight exothermic reaction was observed. The mixture was cooled in a cooling bath (-15 °C – -10 °C, acetone/ice 50:50), compound **12** (355 mg, 1.30 mmol) was added in one portion via a syringe.* After 1.5 h a solution of aldehyde **9** (282 mg, 1.00 mmol) in THF (1.5 mL) was added dropwise. The cooling bath was removed and the solution was stirred at rt for 45 min. The reaction mixture was poured into 1 M NH_4Cl_{aq} solution (50 mL) to which was added ethyl acetate (40 mL). The turbid biphasic mixture was stirred for 15 min until both layers became clear. The organic layer was separated and the aqueous layer was extracted with EtOAc (2 × 60 mL), the combined organic layers were washed with brine (50 mL), dried over $MgSO_4$ and concentrated by means of rotary evaporation. The residue was loaded onto silica for purification by column chromatography (EtOAc/pentane, gradient, 20:80 and 50:50), providing lactone **11** (291 mg, 0.699 mmol, 70% yield) as a pale yellow crystalline solid.

^{1}H-NMR (400 MHz, $CDCl_3$) δ 8.32 (s, 1H), 7.67 (d, J = 9.0 Hz, 1H), 7.45 (t, J = 7.9 Hz, 1H), 7.20 (s, 1H), 7.07 (d, J = 9.1 Hz, 1H), 6.90 – 6.82 (m, 2H), 6.80 (s, 1.6 Hz, 1H), 6.05 (ddt, J = 17.2, 10.8, 5.5 Hz, 1H), 5.66 (ddt, J = 16.5, 11.0, 5.6 Hz, 1H), 5.44 – 5.35 (m, 1H), 5.25 – 5.16 (m, 1H), 5.09 – 4.91 (m, 2H), 4.78 – 4.63 (m, 2H), 4.46 – 4.38 (m, 1H), 4.17 – 4.09 (m, 1H), 4.01 (s, 3H), 2.46 (s, 3H).

^{13}C-NMR (101 MHz, $CDCl_3$) δ 170.2, 158.1, 156.5, 154.8, 154.7, 135.3, 133.3, 132.9, 132.7, 131.3, 130.6, 123.6, 121.2, 118.4, 117.7, 117.6, 115.8, 114.9, 113.9, 110.9, 109.6, 78.3, 70.3, 70.2, 55.9, 21.5.

HRMS calcd for $C_{26}H_{24}O_5$ $\{M+H\}^+$: 417.1697 found: 417.1701.

Mp 135 – 138 °C.

Note:
*bromo-magnesium exchange under these conditions likely leads to the formation of a *bis*-aryl magnesiate species.[8] After 1.5 h its formation stagnates and the conversion is estimated at approximately 75%, according to GC-MS. (The bromide isotopes are diagnostic)

Pratenone A 2

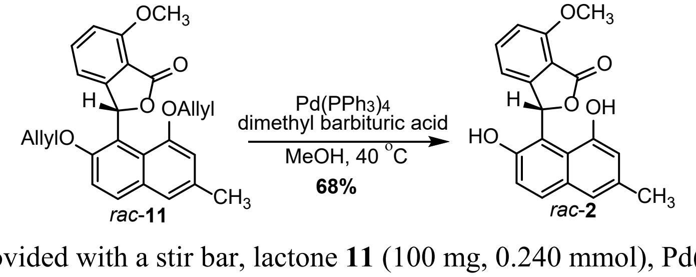

A 20 mL vial was provided with a stir bar, lactone **11** (100 mg, 0.240 mmol), $Pd(PPh_3)_4$ (21.0 mg, 0.018 mmol, 7.6 mol%) and dimethyl barbituric acid (37.5 mg, 0.240 mmol). The solids were suspended in MeOH, the mixture was heated at 40 °C for 1.5 h. Methanol (30 mL) and DCM (10 mL) were added to solubilise the solids that had collected in the reaction mixture, five drops of water and ammonium

pyrrolidine dithiocarbamate (12 mg, 0.73 mmol) were added as a palladium scavenger. The mixture was stirred for 1 h at rt, then the mixture was pressed through a PTFE filter disk. The filtrate was concentrated and loaded on celite to be chromatographed on an automated Grace flash column chromatography system. With a gradient 0 to 4% MeOH, the compound eluted at 3% MeOH. Evaporation of the main fraction gave the product (88.9 mg) as a brown solid, which was re-dissolved in MeOH (10 mL), water was added and the brown precipitate was separated by centrifugation at 7000 rpm – 9000 rpm. The supernatant was removed by a pipette and the pellet dissolved in MeOH, concentrated by means of rotary evaporation using a 100 mL round bottomed flask, and further dried by co-evaporation with toluene (40 mL), this provided pratenone A **2** (62 mg, 0.162 mmol, 68% yield corrected using ^{1}H-NMR integrations) as a brown solid, which contained approximately 12% triphenyl phosphine oxide as an impurity. An analytically pure sample was obtained by triturating 10 mg of the compound with toluene (3 × 2 mL).

^{1}H-NMR (400 MHz, $(CD_3)_2SO$) δ 10.28 (s, 1H), 9.41 (s, 1H), 8.39 (s, 1H), 7.60 (d, *J* = 8.9 Hz, 1H), 7.55 (t, *J* = 7.9 Hz, 1H), 7.09 (s, 1H), 7.03 (d, *J* = 8.2 Hz, 1H), 6.87 (d, *J* = 8.8 Hz, 1H), 6.83 (d, *J* = 7.6 Hz, 1H), 6.80 (d, *J* = 1.7 Hz, 1H), 3.91 (s, 3H), 2.34 (s, 13H).

^{13}C-NMR (101 MHz, $(CD_3)_2SO$) δ 169.3, 157.8, 155.1, 154.6, 153.3, 136.1, 132.4, 130.9, 130.7, 122.6, 119.8, 119.2, 114.6, 114.2, 113.8, 113.5, 110.6, 77.9, 56.1, 21.2.

HRMS calc for $C_{20}H_{16}O_5$ $\{M+H\}^+$: 337.1071, found: 337.1077.

The physical data are in agreement with those reported for natural pratenone A **2**.[9]

References

(1) Verga, D.; Percivalle, C.; Doria, F.; Porta, A.; Freccero, M. Protecting Group Free Synthesis of 6-Substituted Naphthols and Binols. *J. Org. Chem.* **2011**, *76* (7), 2319–2323.
(2) García, O.; Nicolás, E.; Albericio, F. O-Formylation of Electron-Rich Phenols with Dichloromethyl Methyl Ether and TiCl4. *Tetrahedron Lett.* **2003**, *44* (27), 4961–4963.
(3) Dubost, E.; Fossey, C.; Cailly, T.; Rault, S.; Fabis, F. Selective *Ortho* -Bromination of Substituted Benzaldoximes Using Pd-Catalyzed C–H Activation: Application to the Synthesis of Substituted 2-Bromobenzaldehydes. *J. Org. Chem.* **2011**, *76* (15), 6414–6420.
(4) Desai, L. V.; Malik, H. A.; Sanford, M. S. Oxone as an Inexpensive, Safe, and Environmentally Benign Oxidant for C−H Bond Oxygenation. *Org. Lett.* **2006**, *8* (6), 1141–1144.
(5) Snyder, S. A.; Sherwood, T. C.; Ross, A. G. Total Syntheses of Dalesconol A and B. *Angew. Chem. Int. Ed.* **2010**, *49* (30), 5146–5150.
(6) Yixizhuoma; Tsukahara, K.; Toume, K.; Ishikawa, N.; Abdelfattah, M. S.; Ishibashi, M. Novel Cytotoxic Isobenzofuran Derivatives from Streptomyces Sp. IFM 11490. *Tetrahedron Lett.* **2015**, *56* (46), 6345–6347.
(7) Raju, R.; Gromyko, O.; Fedorenko, V.; Luzhetskyy, A.; Müller, R. Oleaceran: A Novel Spiro[Isobenzofuran-1,2′-Naptho[1,8- *Bc*]Furan] Isolated from a Terrestrial *Streptomyces* Sp. *Org. Lett.* **2013**, *15* (14), 3487–3489.
(8) Krasovskiy, A.; Straub, B. F.; Knochel, P. Highly Efficient Reagents for Br/Mg Exchange. *Angew. Chem. Int. Ed.* **2006**, *45* (1), 159–162.
(9) Zhang, S.; Zhang, L.; Kou, L.; Yang, Q.; Qu, B.; Pescitelli, G.; Xie, Z. Isolation, Stereochemical Study, and Racemization of (±)-pratenone A, the First Naturally Occurring 3-(1-naphthyl)-2-benzofuran-1(3H)-one Polyketide from a Marine-derived Actinobacterium. *Chirality* **2020**, *32* (3), 299–307.

S2: ^{1}H-NMR and ^{13}C-NMR spectra

^{1}H-NMR (400 MHz, $CDCl_3$)

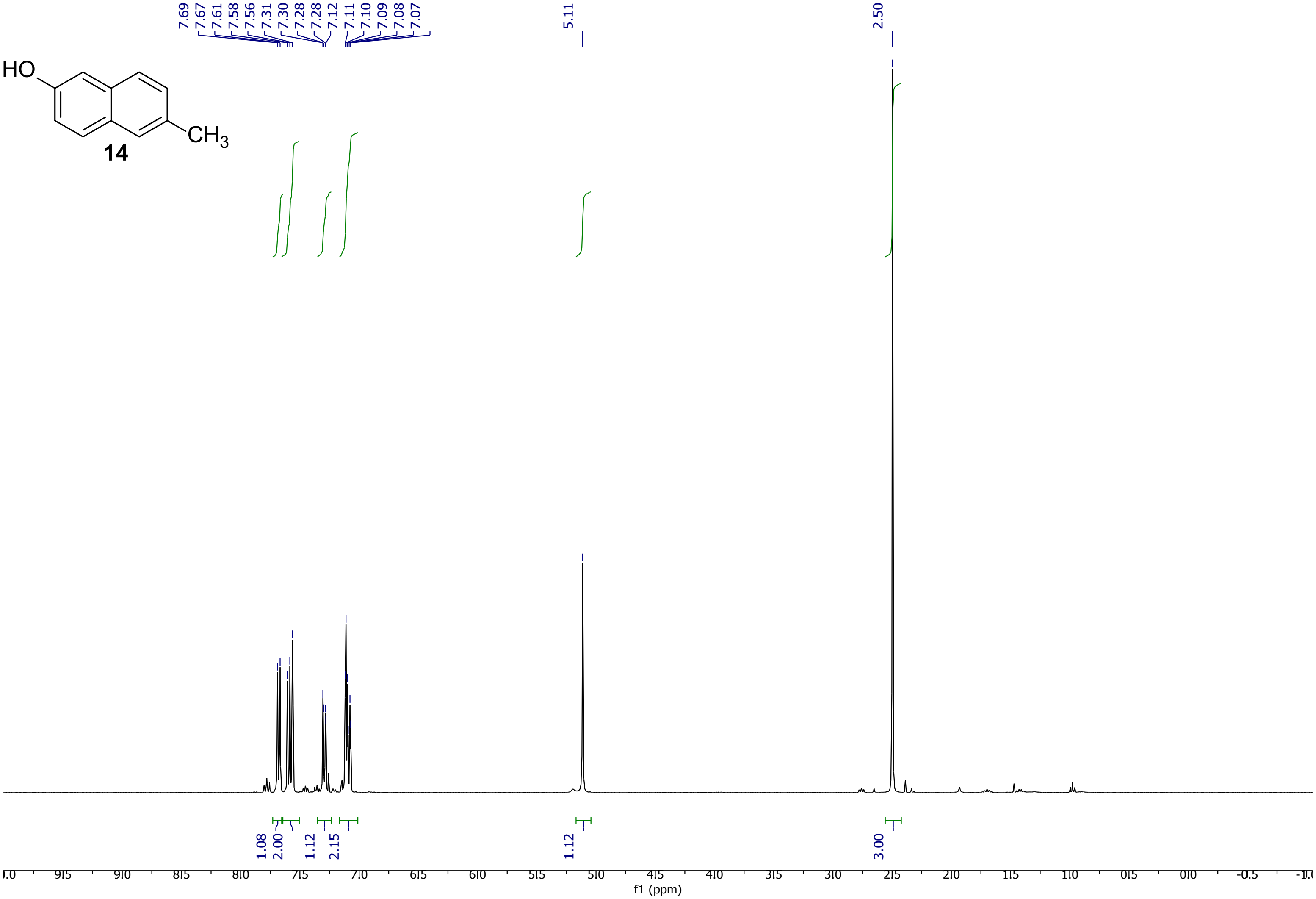

$^{13}C\{^{1}H\}$-NMR APT (101 MHz, $CDCl_3$)

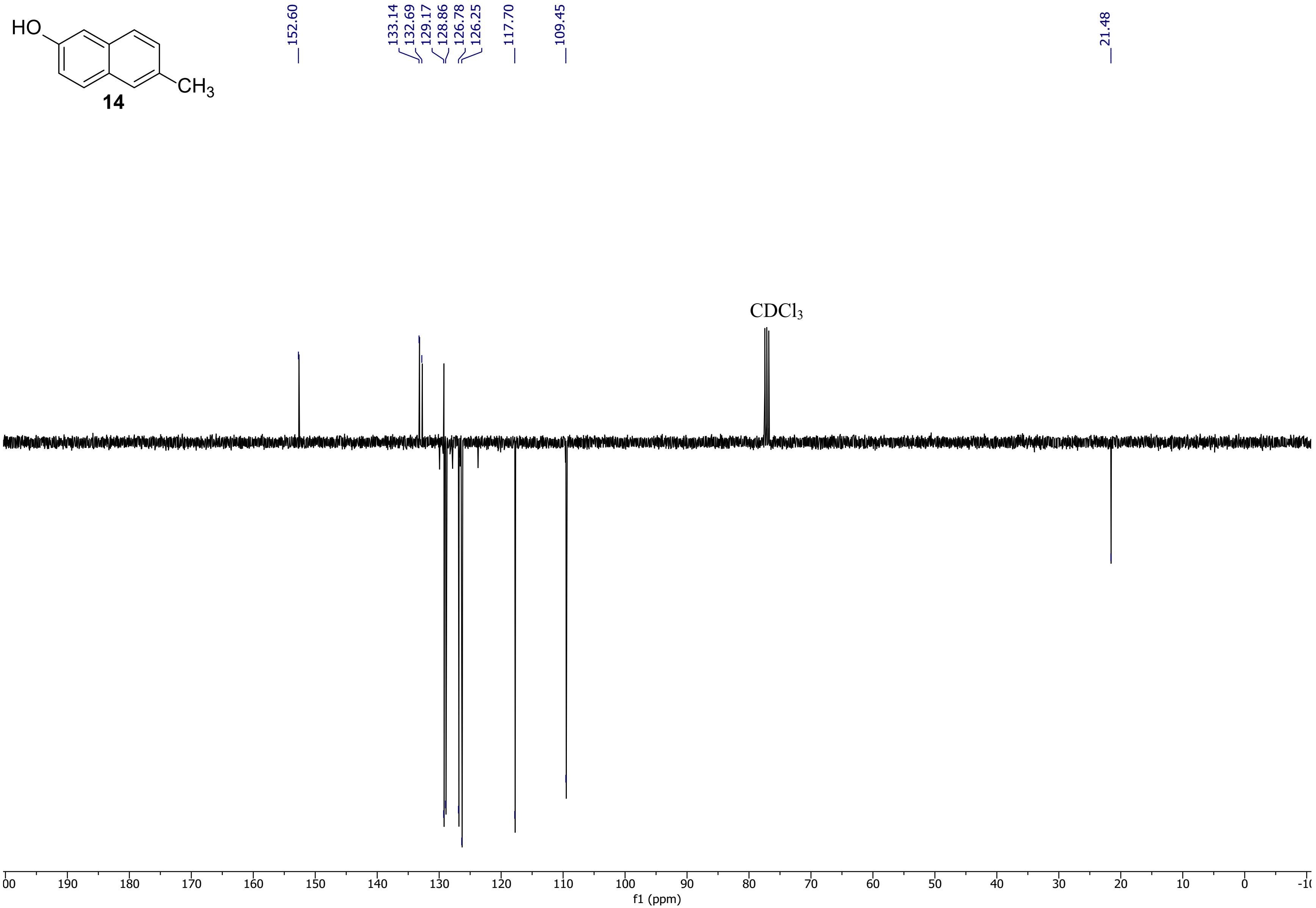

^{1}H-NMR (400 MHz, $CDCl_3$)

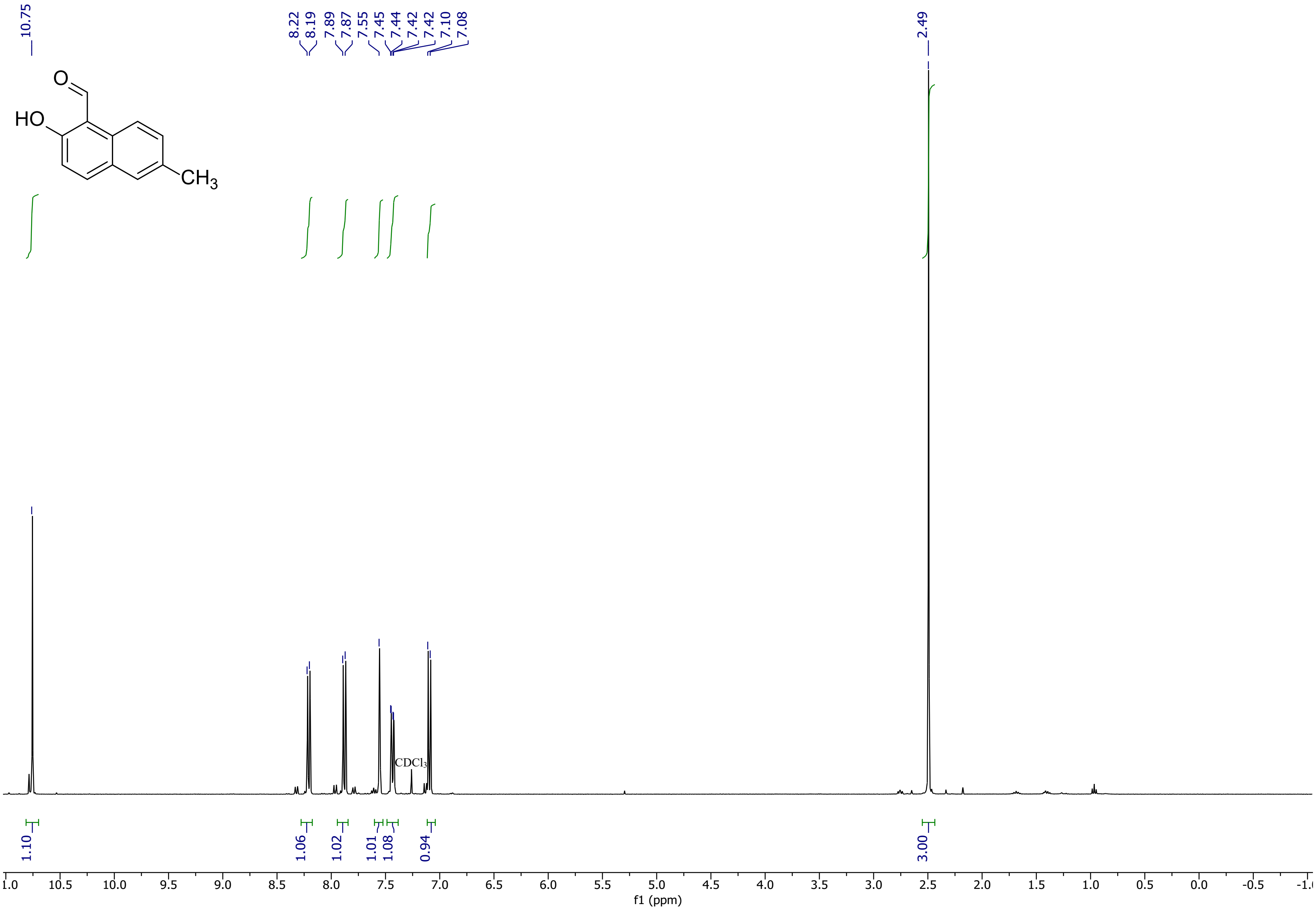

$^{13}C\{^{1}H\}$-NMR APT (101 MHz, $CDCl_3$)

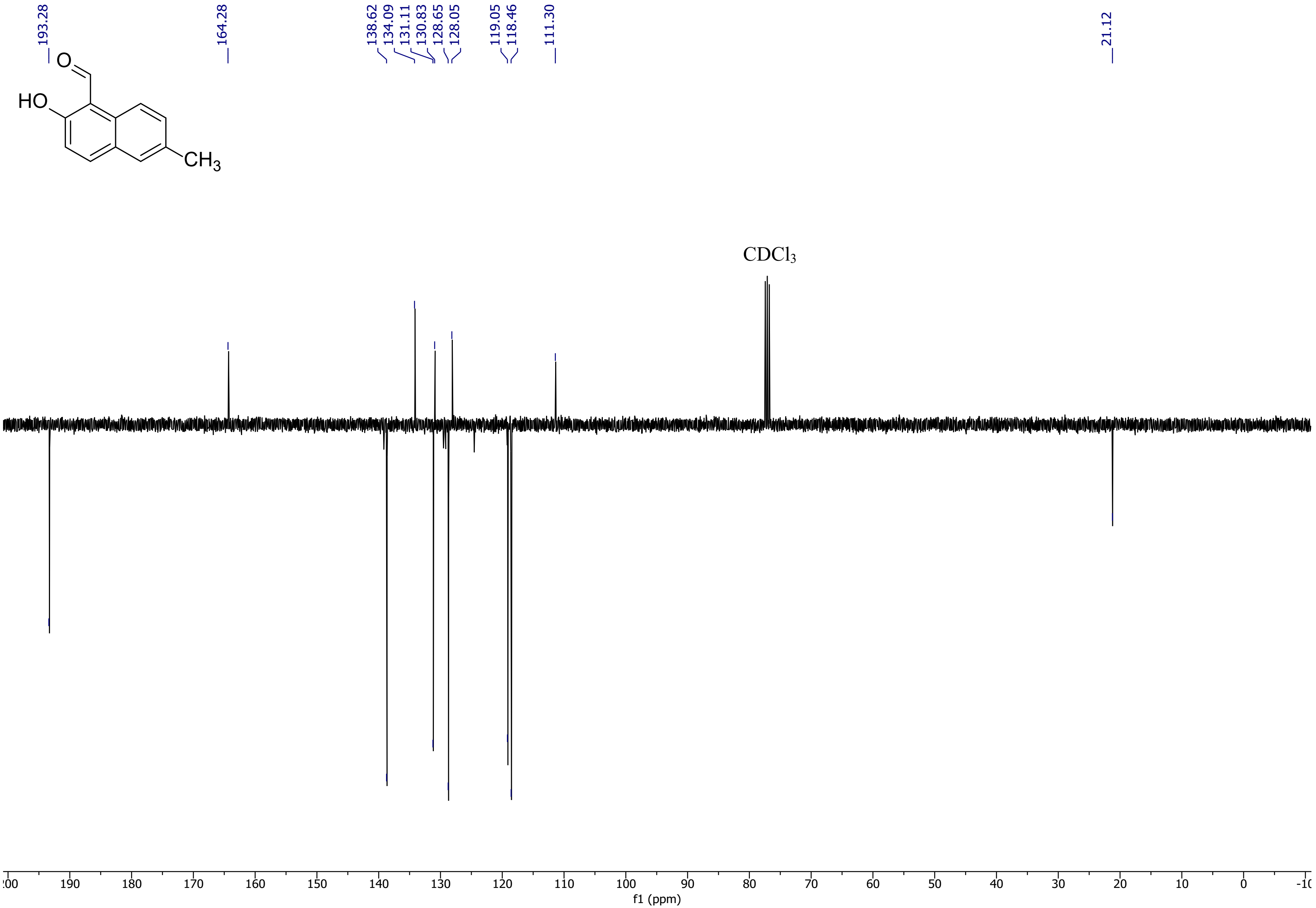

^{1}H-NMR (400 MHz, $CDCl_3$)

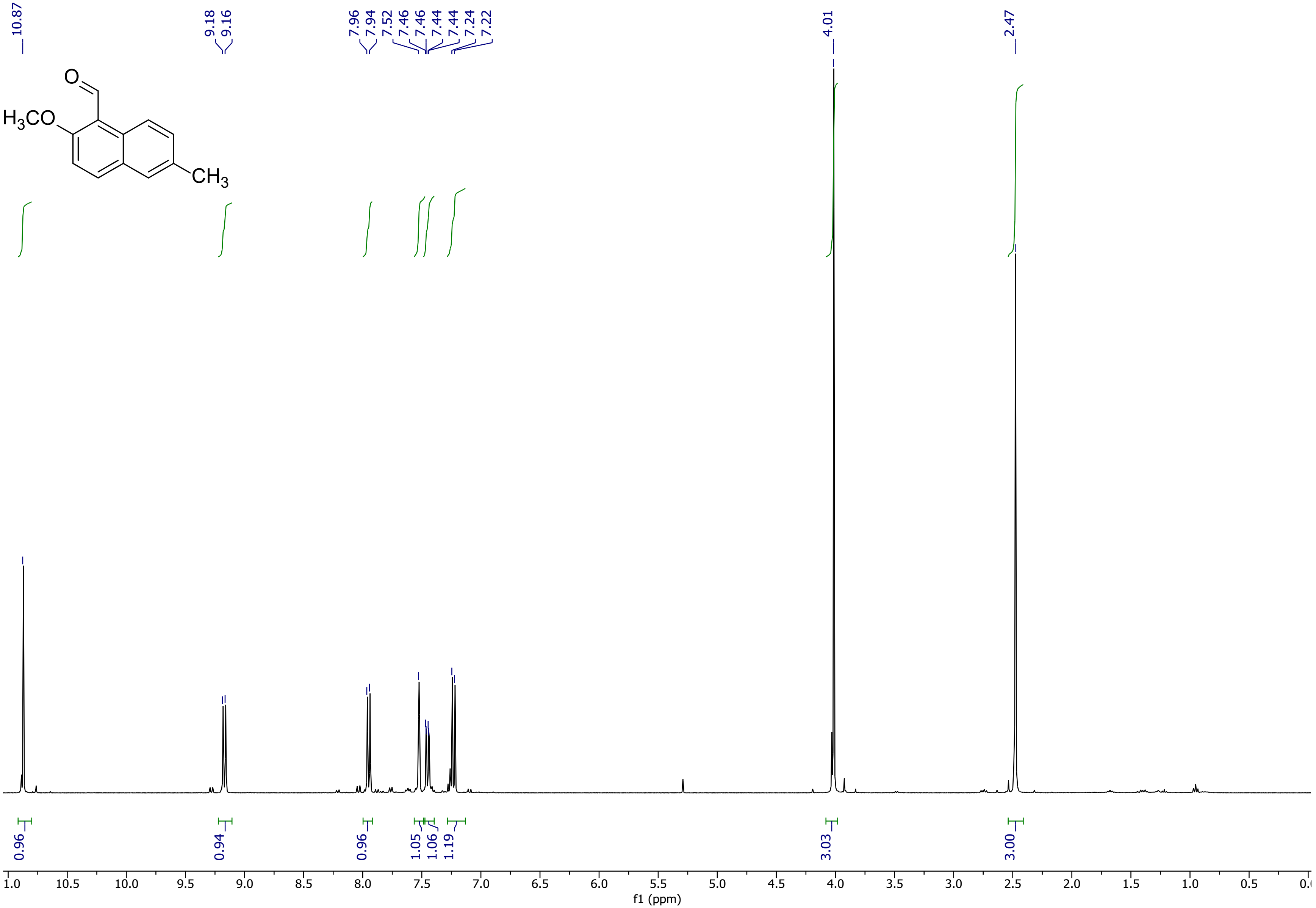

$^{13}C\{^1H\}$-NMR APT (101 MHz, $CDCl_3$)

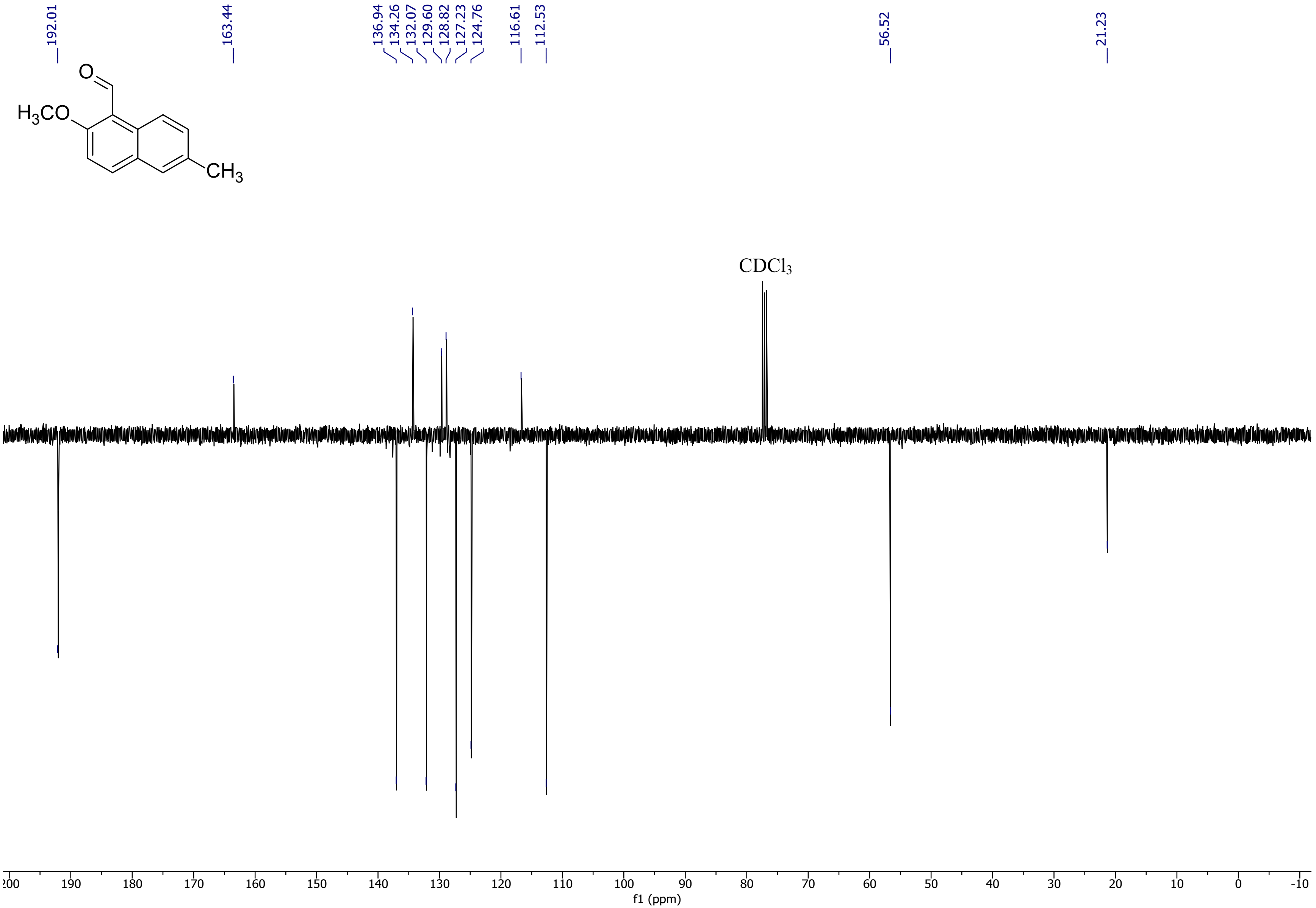

^{1}H-NMR (400 MHz, $CDCl_3$)

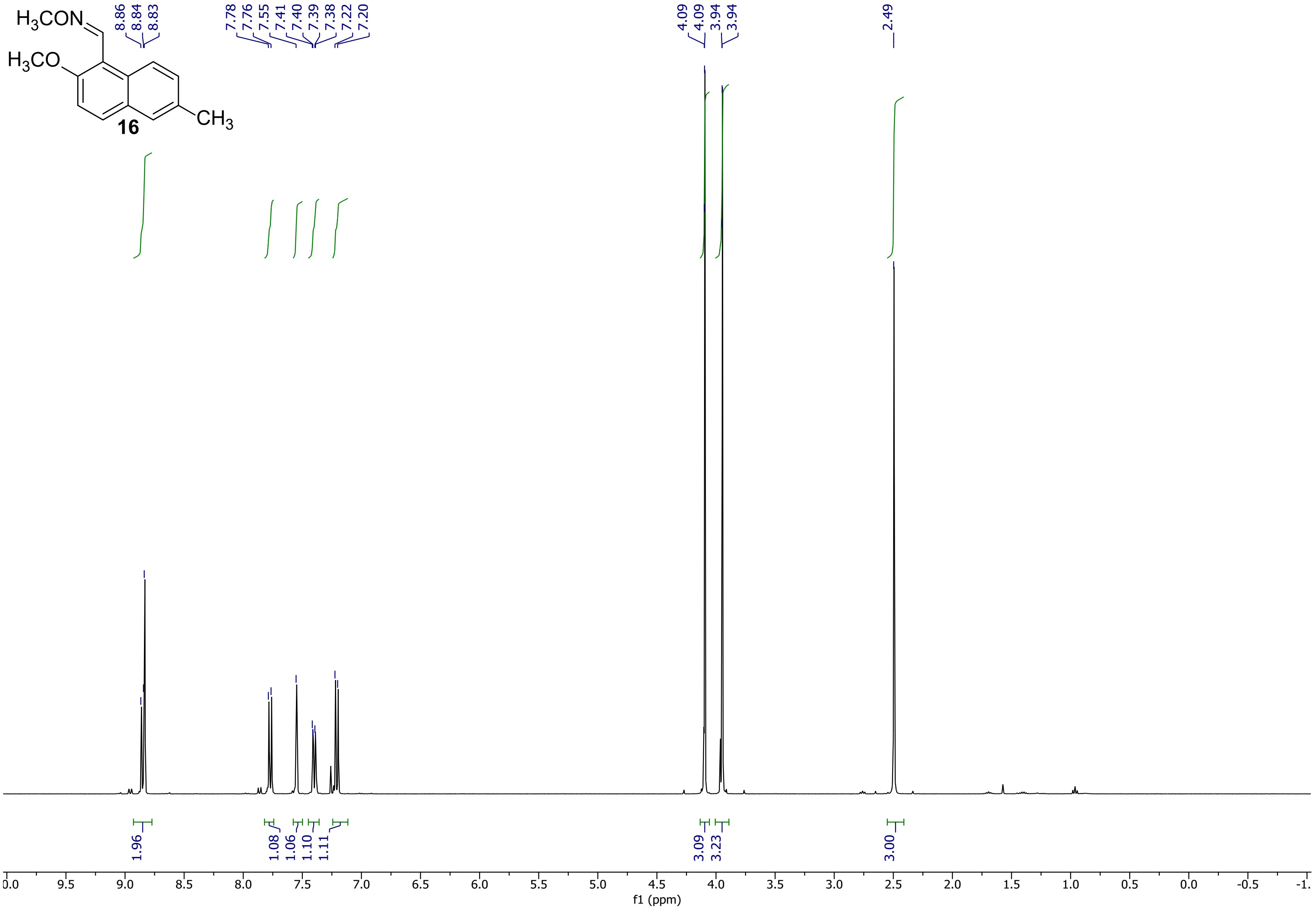

$^{13}C\{^{1}H\}$-NMR APT (101 MHz, $CDCl_3$)

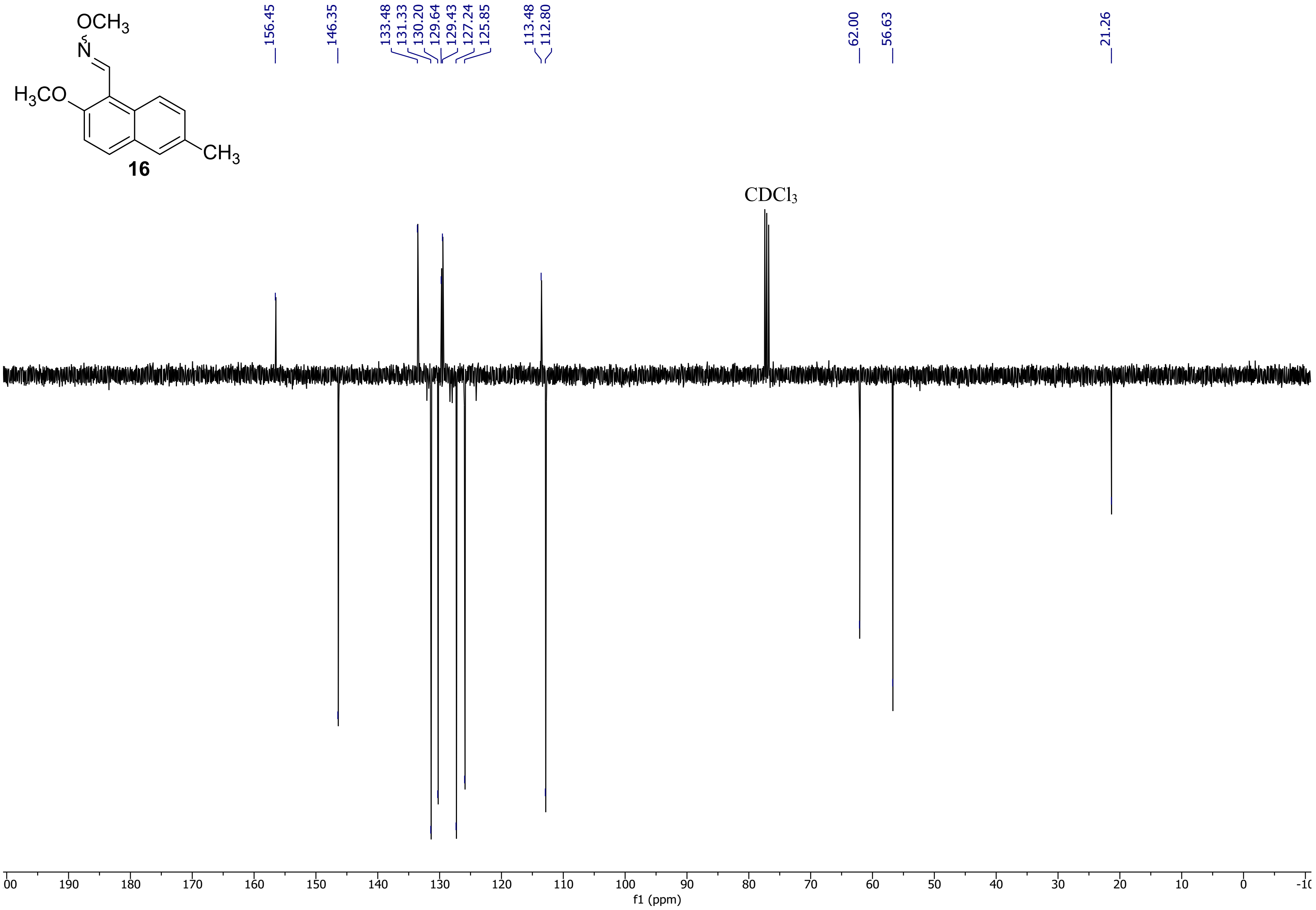

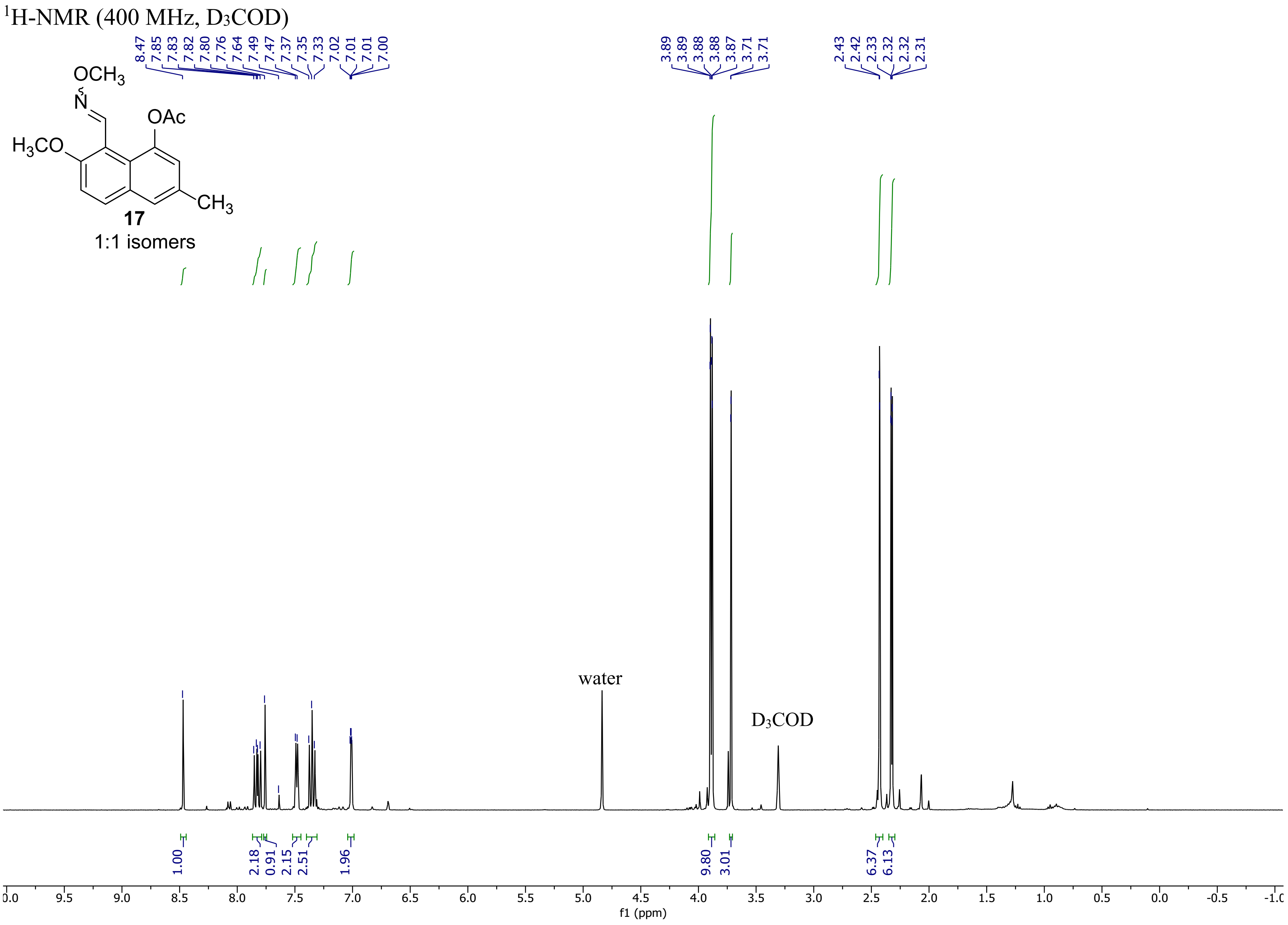

^{1}H-NMR (400 MHz, D3COD)
8.47 7.85 7.83 7.82 7.80 7.76 7.64 7.49 7.47 7.37 7.35 7.33 7.02 7.01 7.01 7.00
3.89 3.89 3.88 3.88 3.87 3.71 3.71
2.43 2.42 2.33 2.32 2.32 2.31
OCH3
OAc
H3CO
CH3
17
1:1 isomers
water
D3COD
1.00 2.18 0.91 2.15 2.51 1.96 9.80 3.01 6.37 6.13
f1 (ppm)

$^{13}C\{^{1}H\}$-NMR (101 MHz, D_3COD)

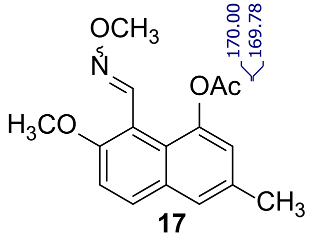

17

1:1 isomers

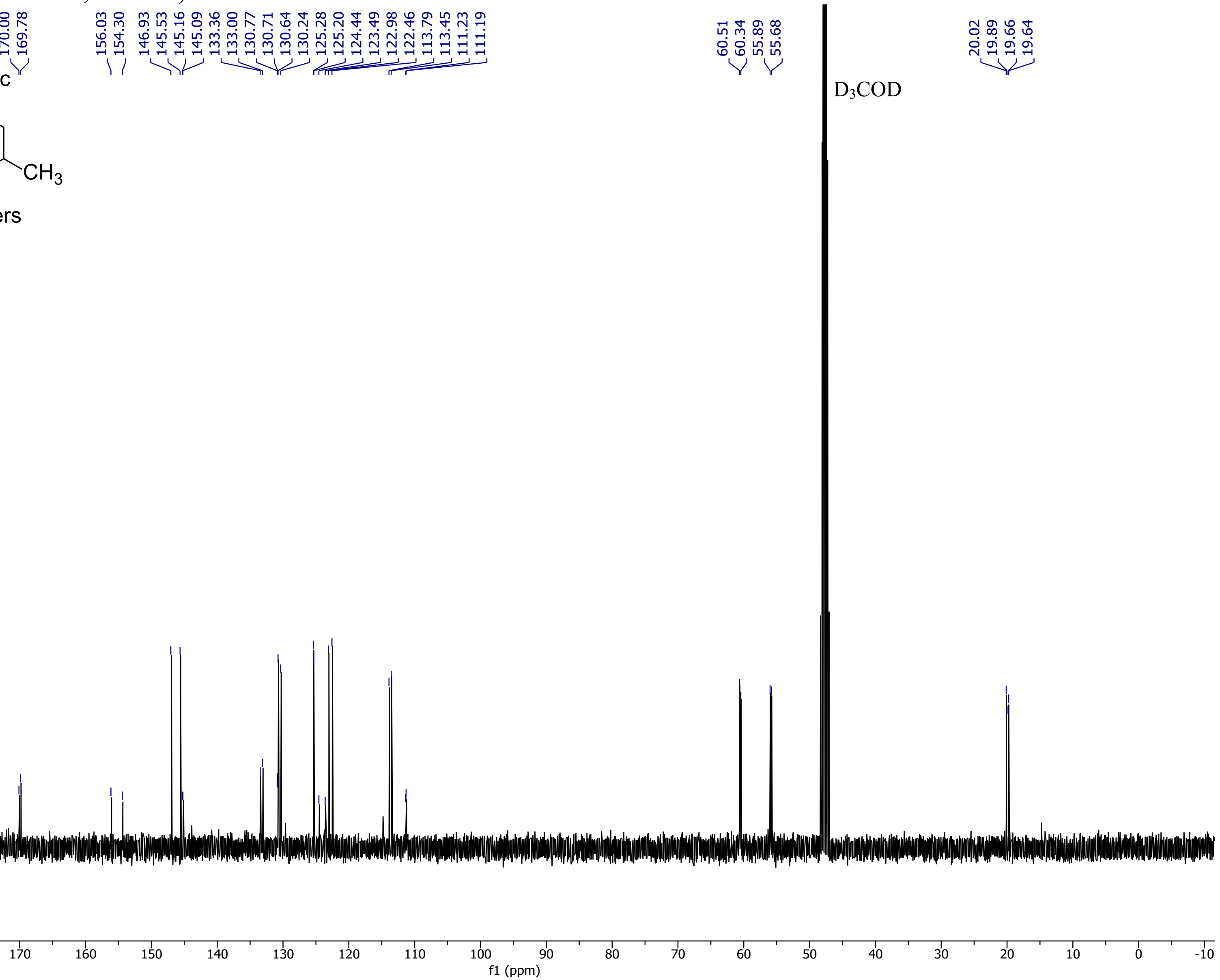

25

^{1}H-NMR (400 MHz, $(D_3C)_2SO$)

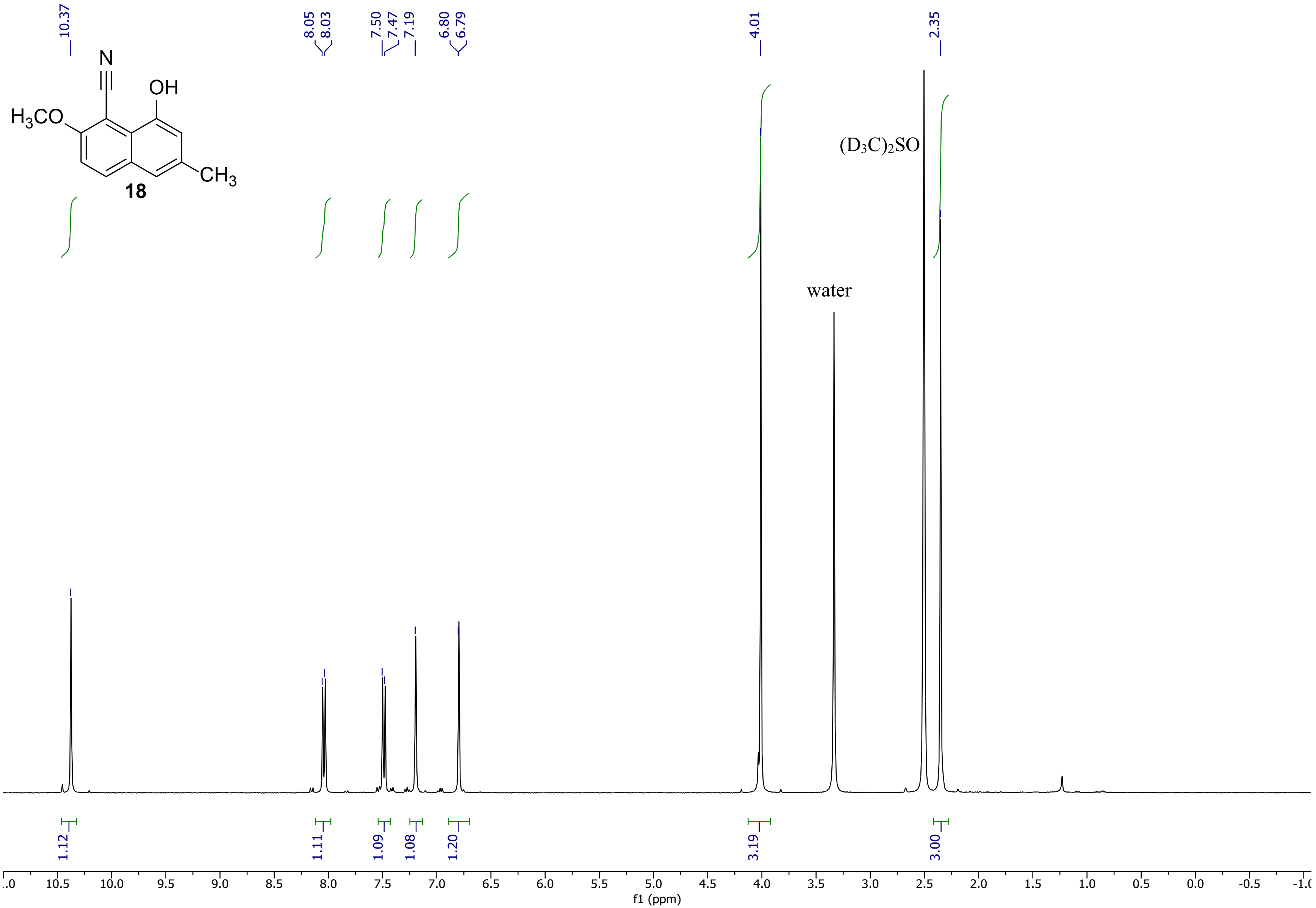

$^{13}C\{^{1}H\}$-NMR (101 MHz, $(D_3C)_2SO$)

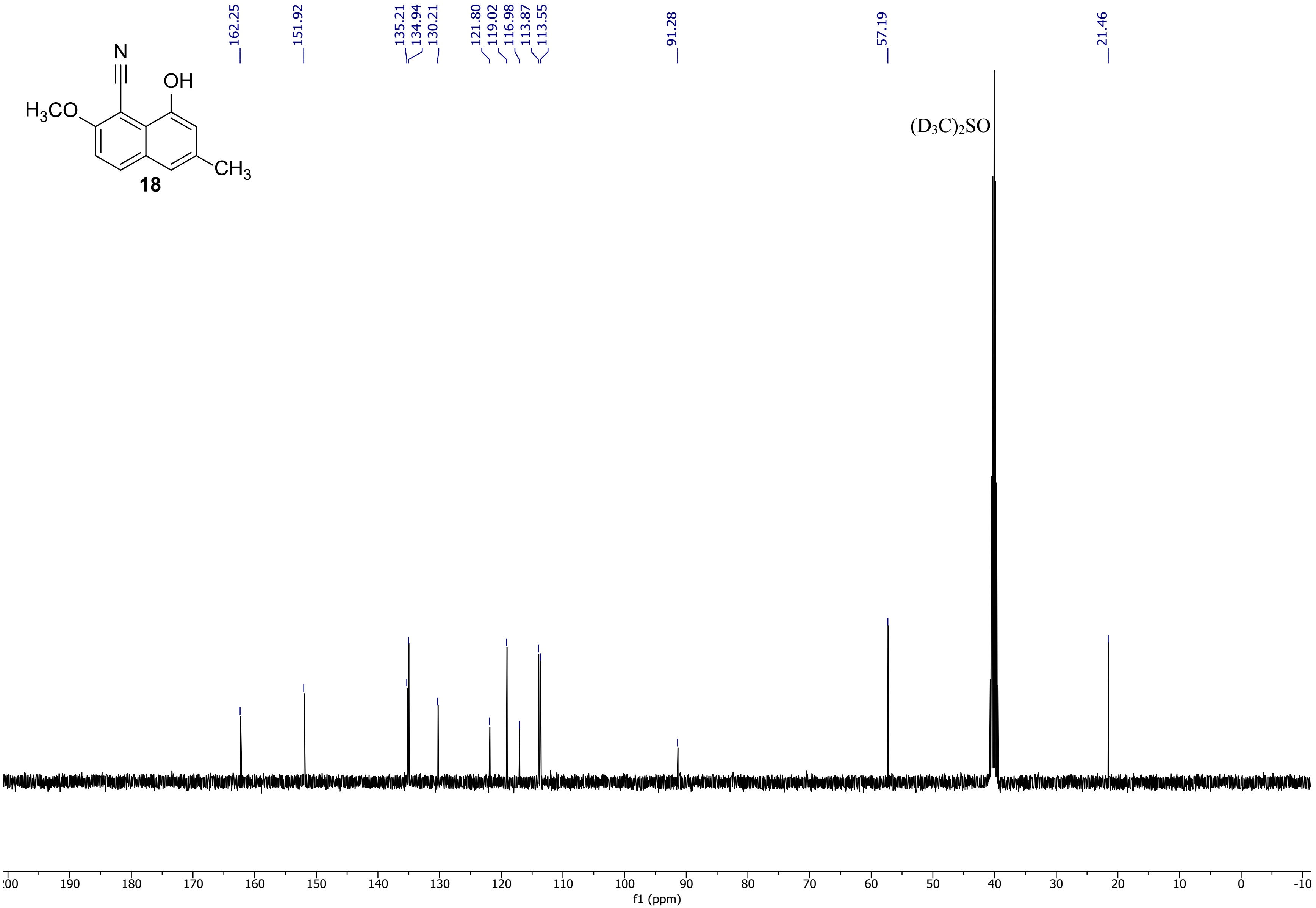

^{1}H-NMR (400 MHz, D_3COD)

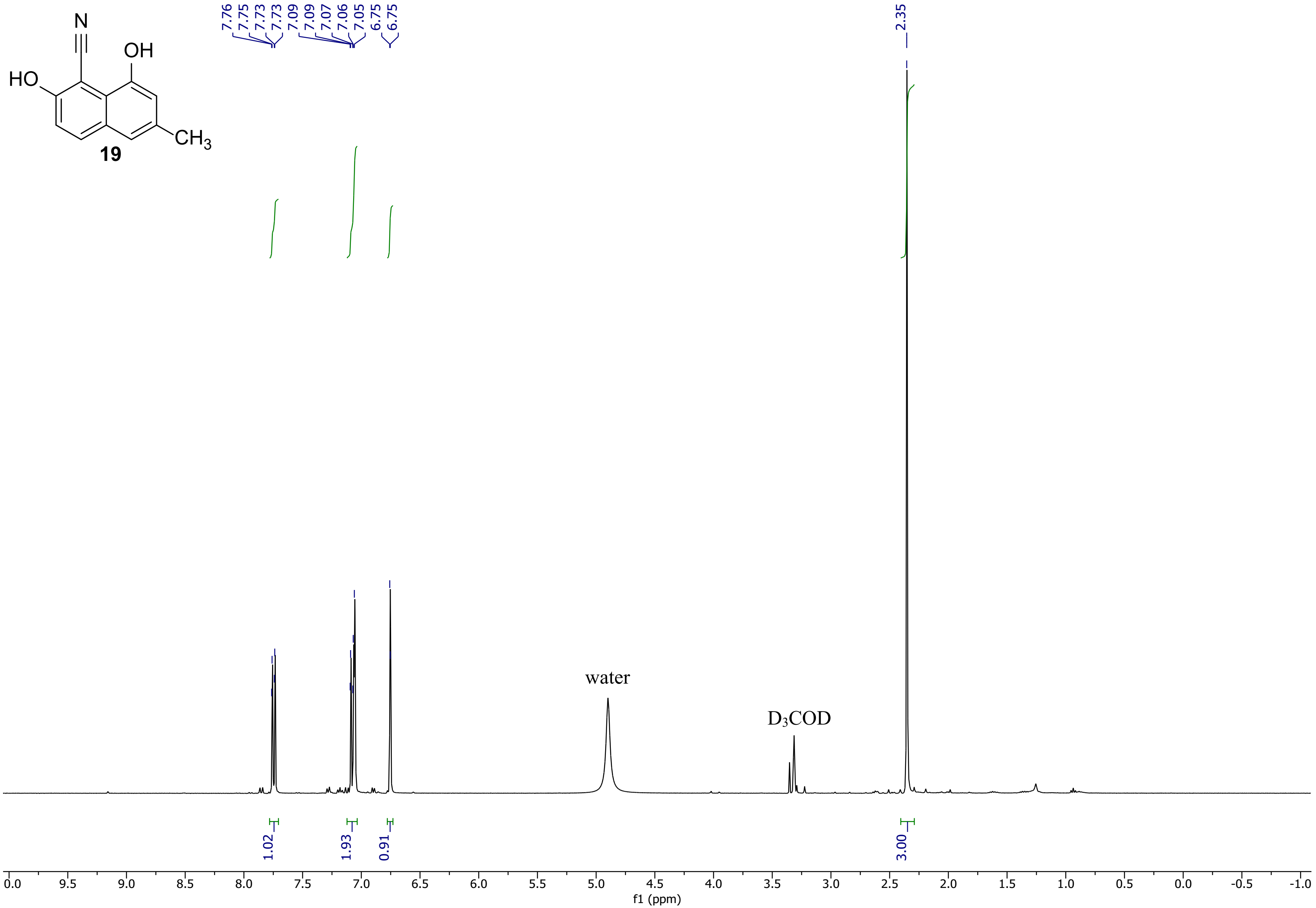

$^{13}C\{^{1}H\}$-NMR APT (101 MHz, D_3COD)

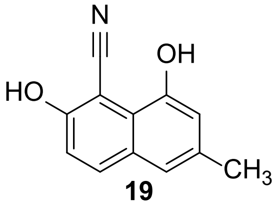

19

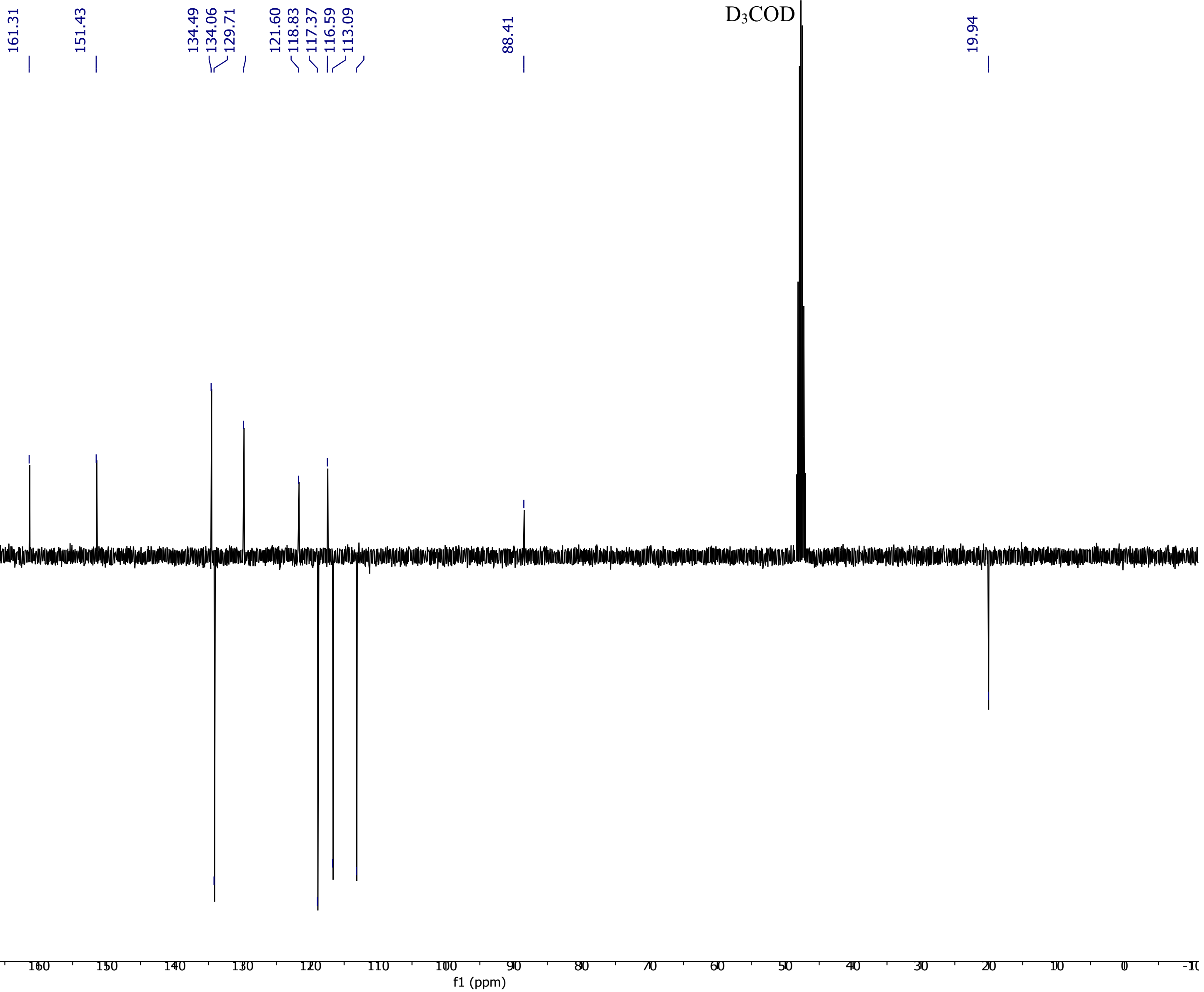

^{1}H-NMR (400 MHz, $CDCl_3$)

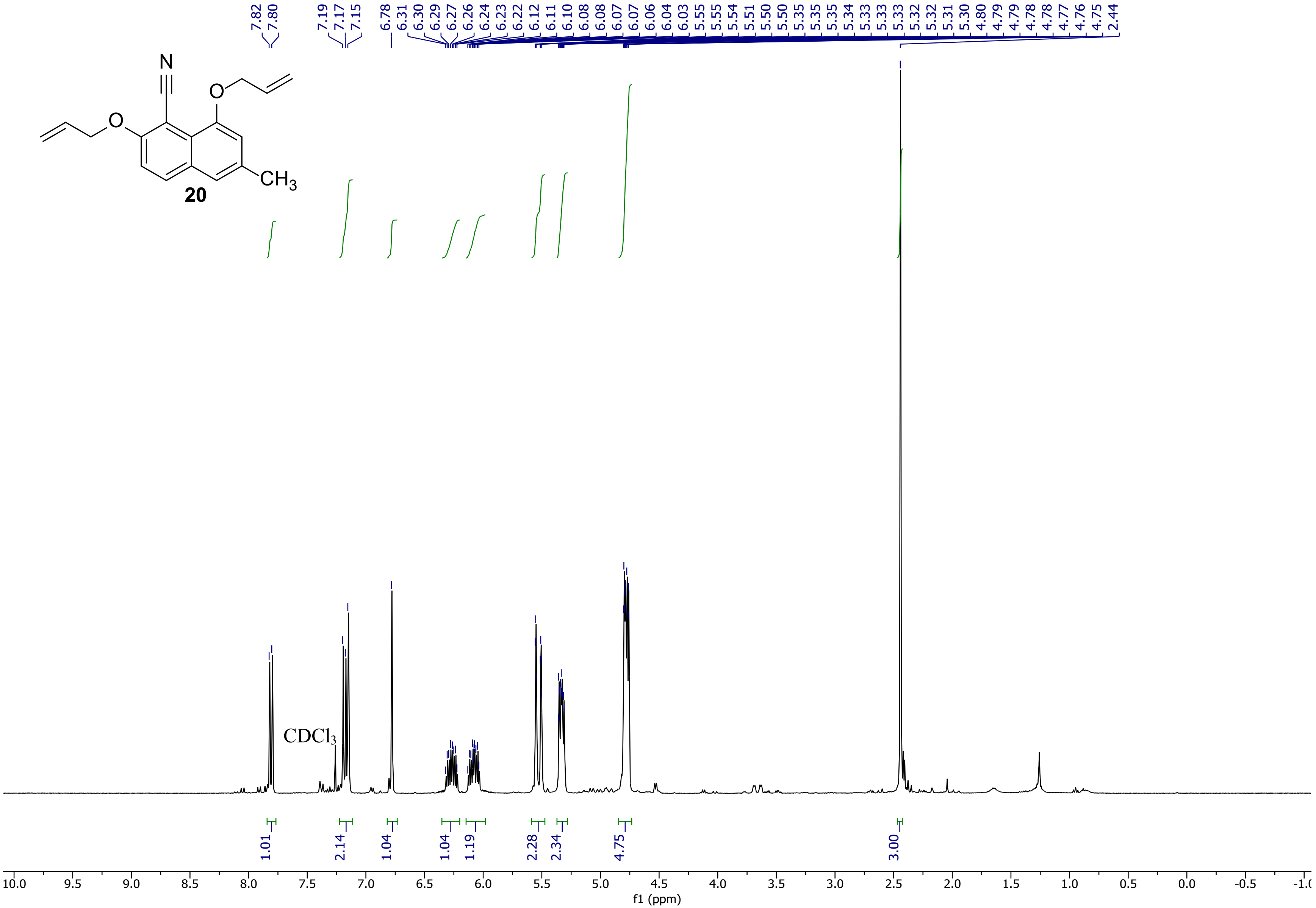

$^{13}C\{^{1}H\}$-NMR APT (101 MHz, $CDCl_3$)

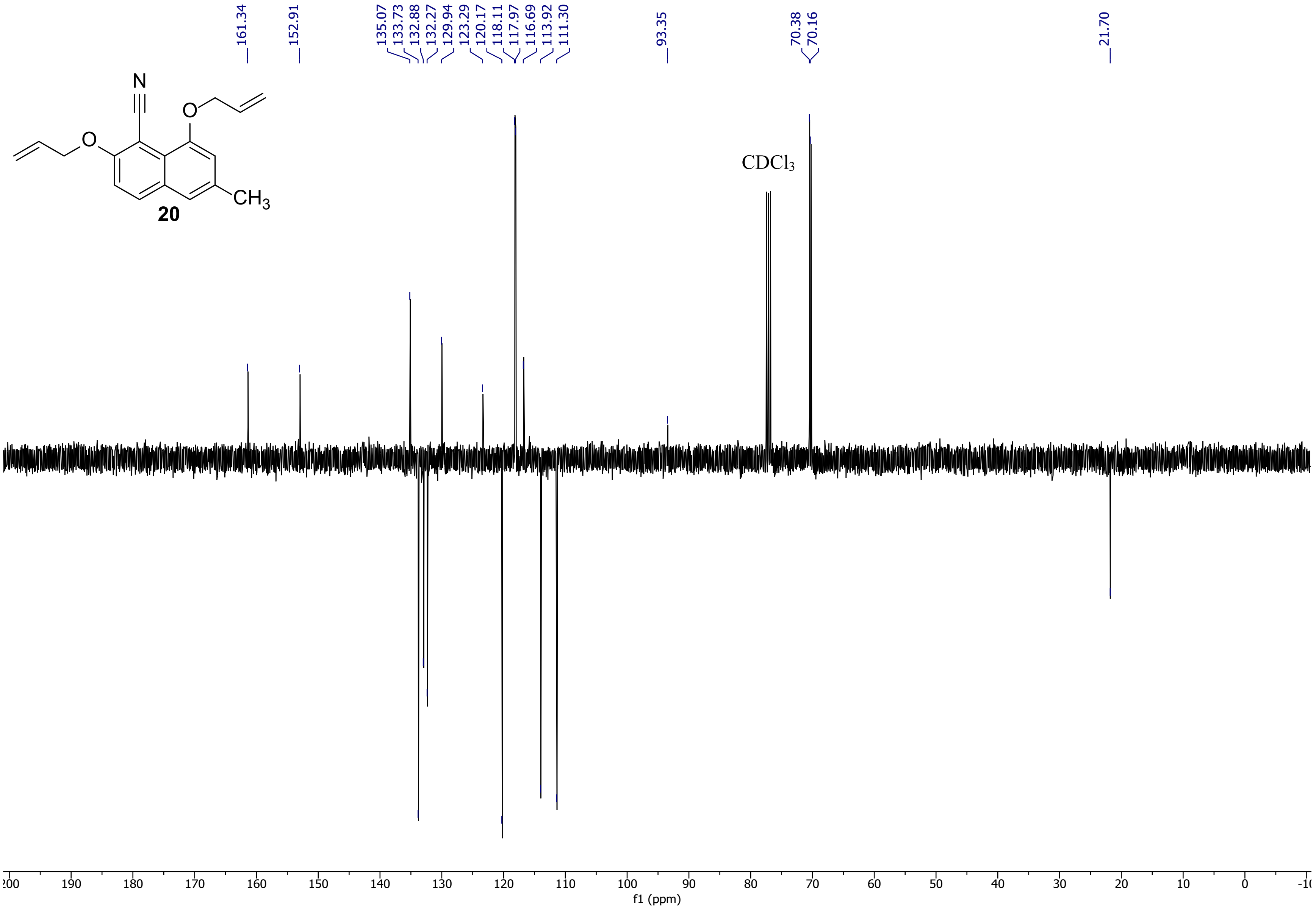

^{1}H-NMR (400 MHz, $CDCl_3$)

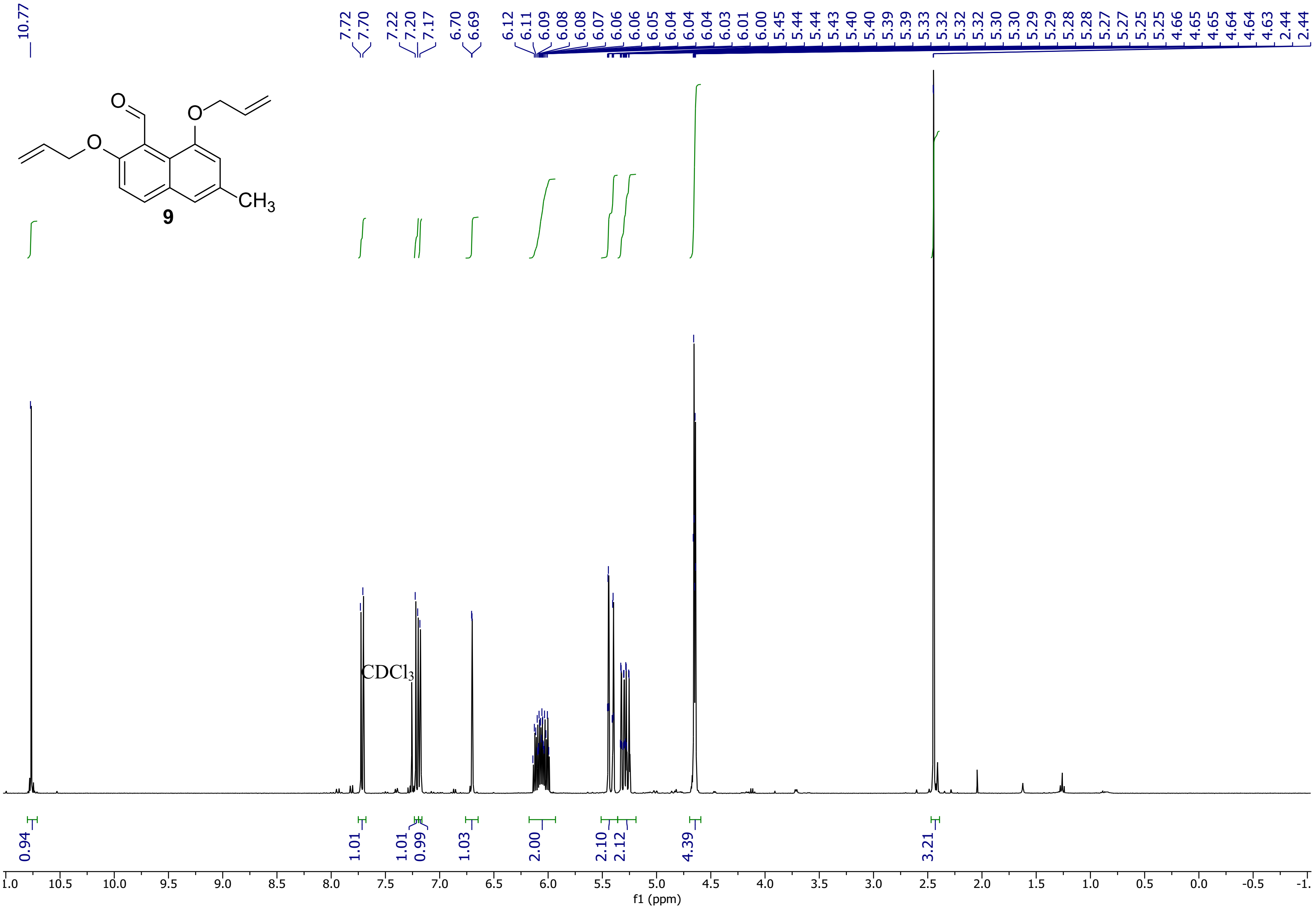

$^{13}C\{^{1}H\}$-NMR APT (101 MHz, $CDCl_3$)

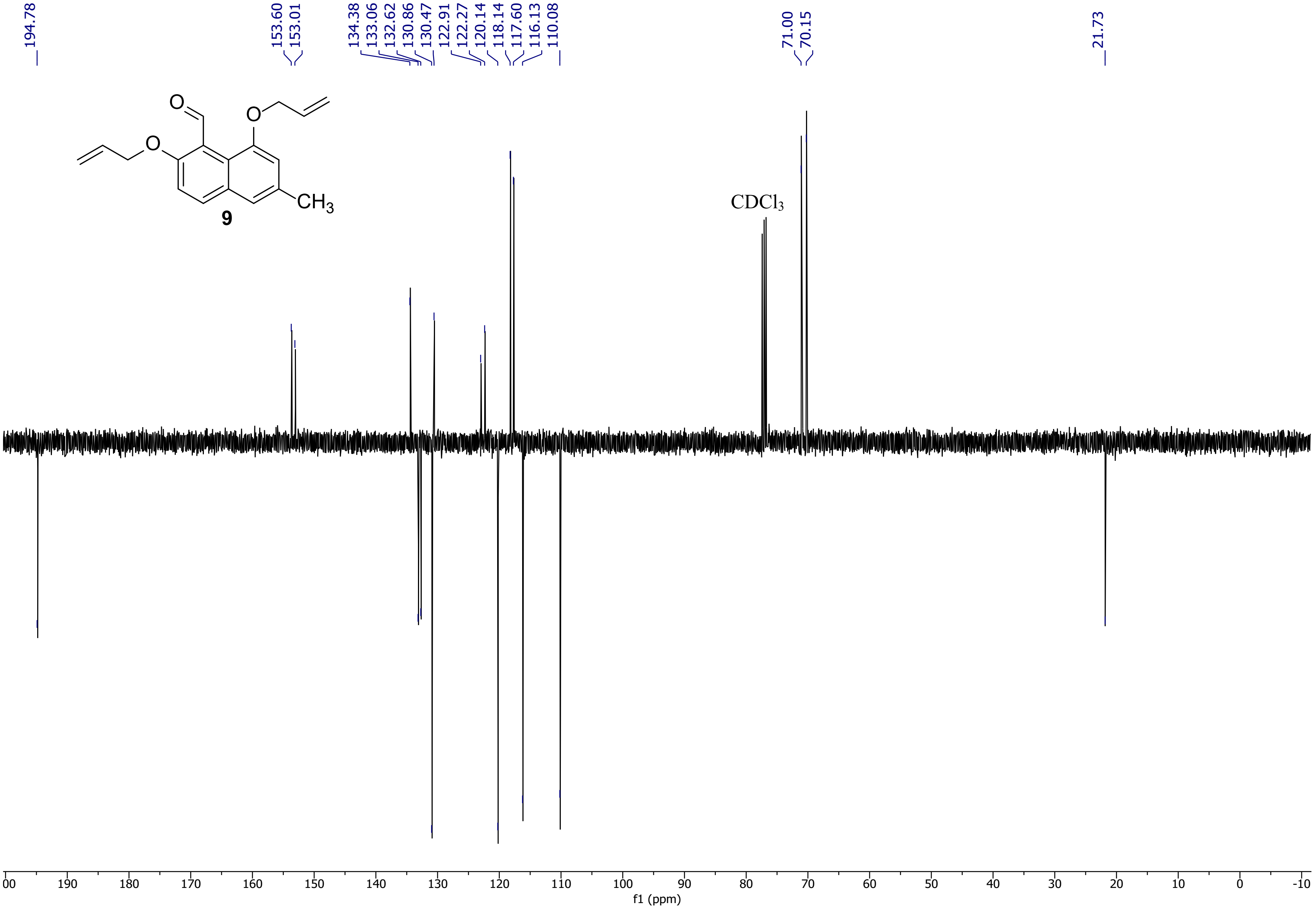

^{1}H-NMR (400 MHz, $CDCl_3$)

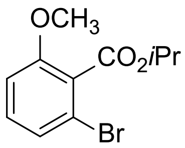

21

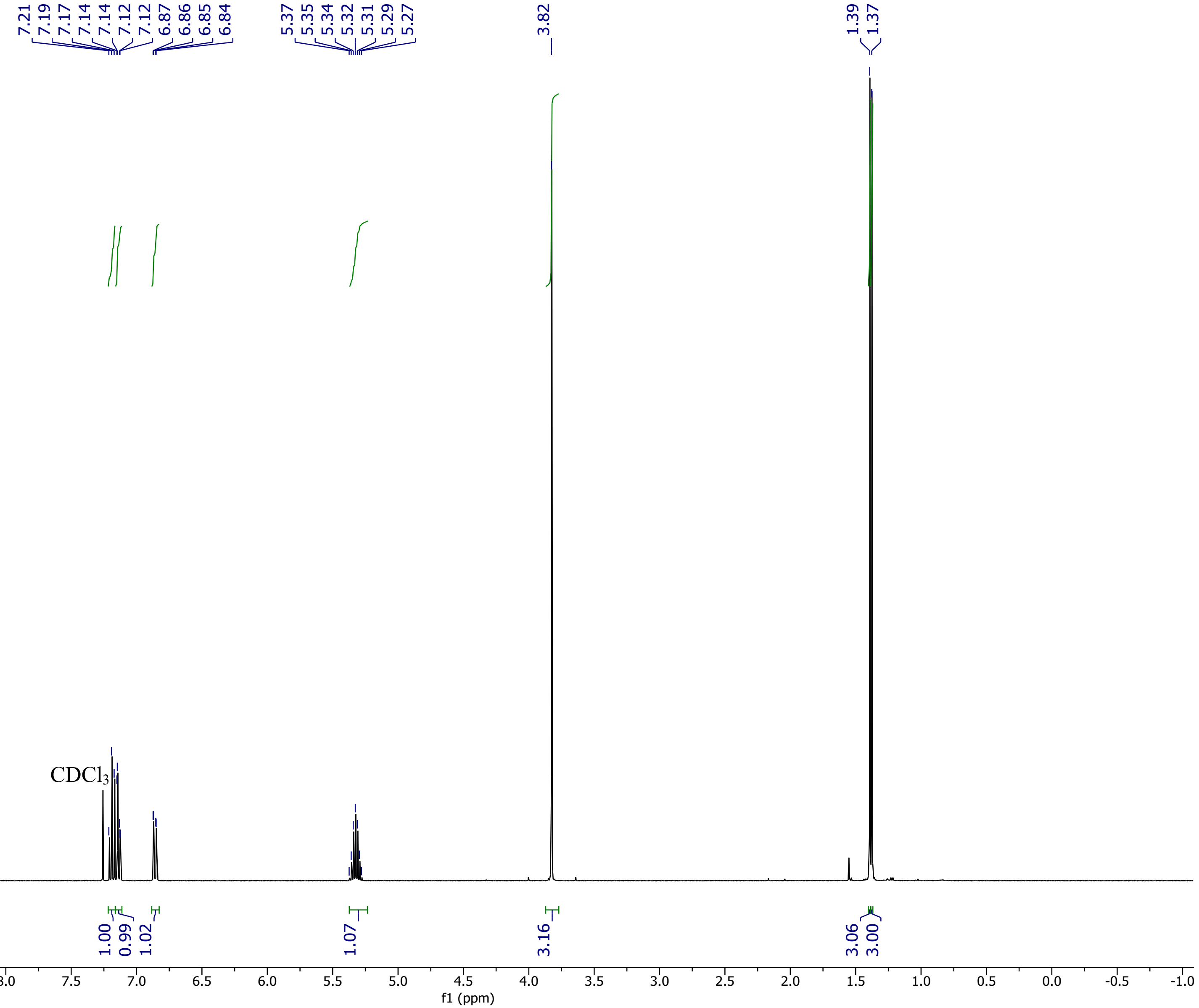

$^{13}C\{^1H\}$-NMR APT (101 MHz, $CDCl_3$)

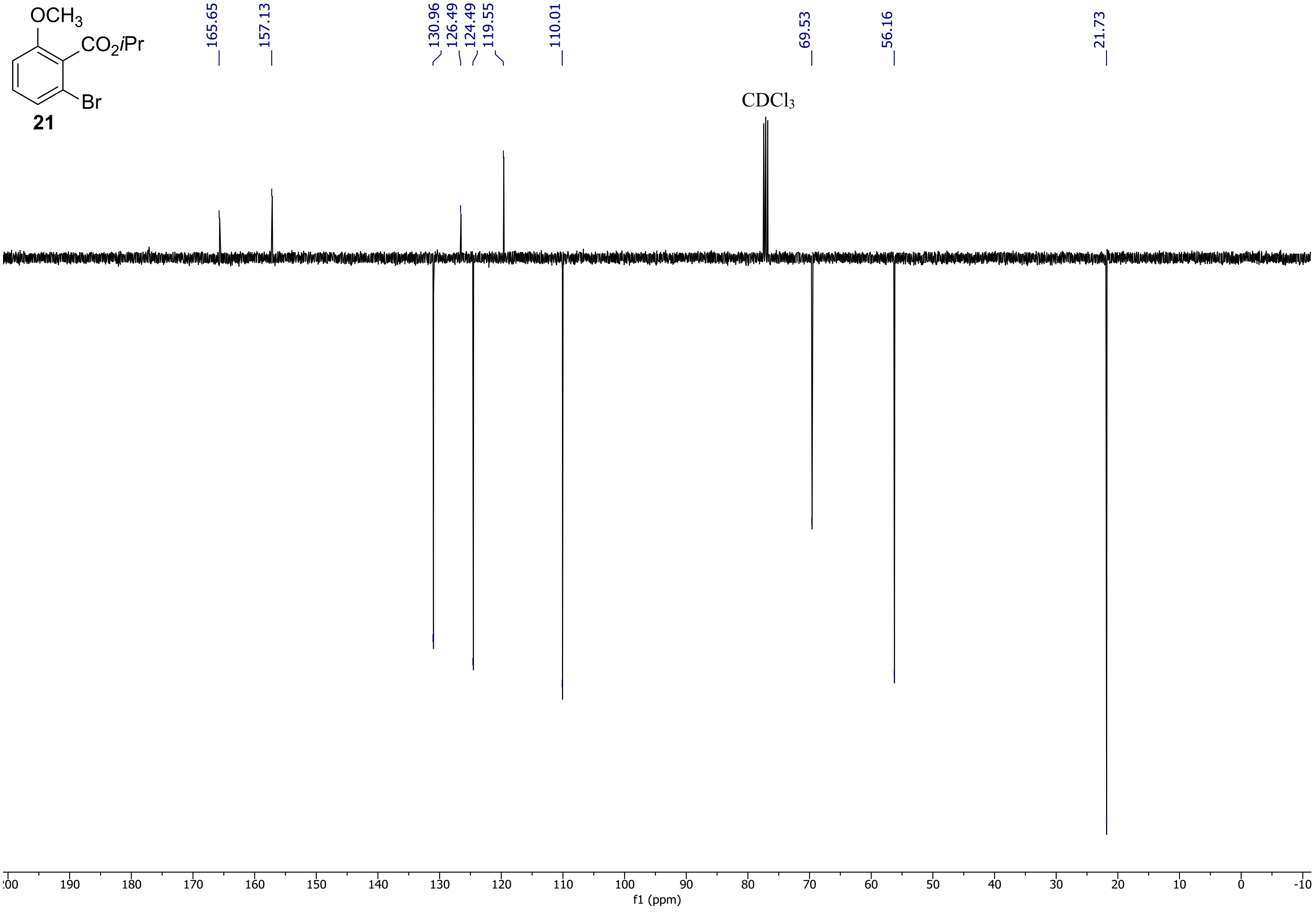

^{1}H-NMR (400 MHz, $CDCl_3$)

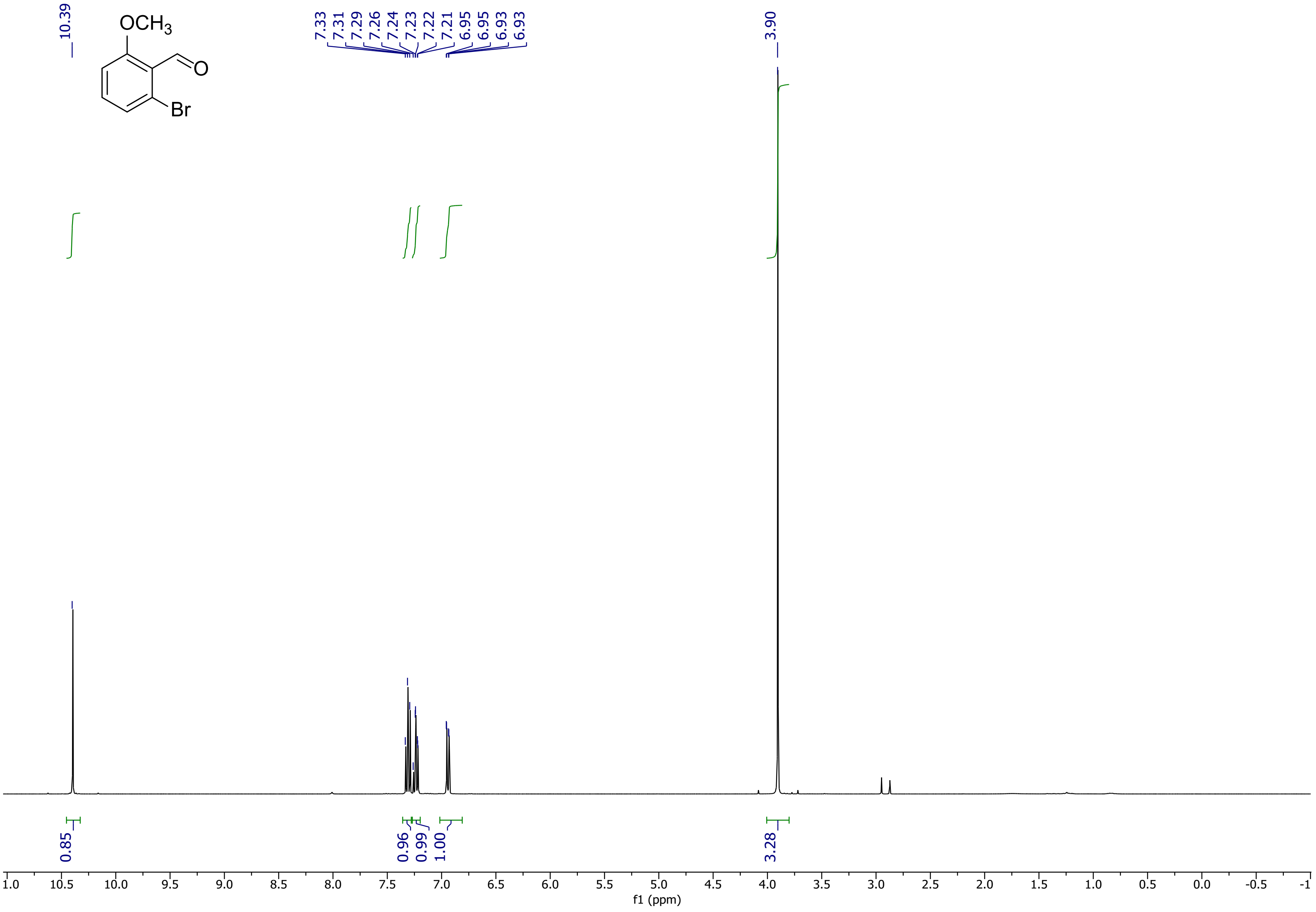

$^{13}C\{^{1}H\}$-NMR APT (101 MHz, $CDCl_3$)

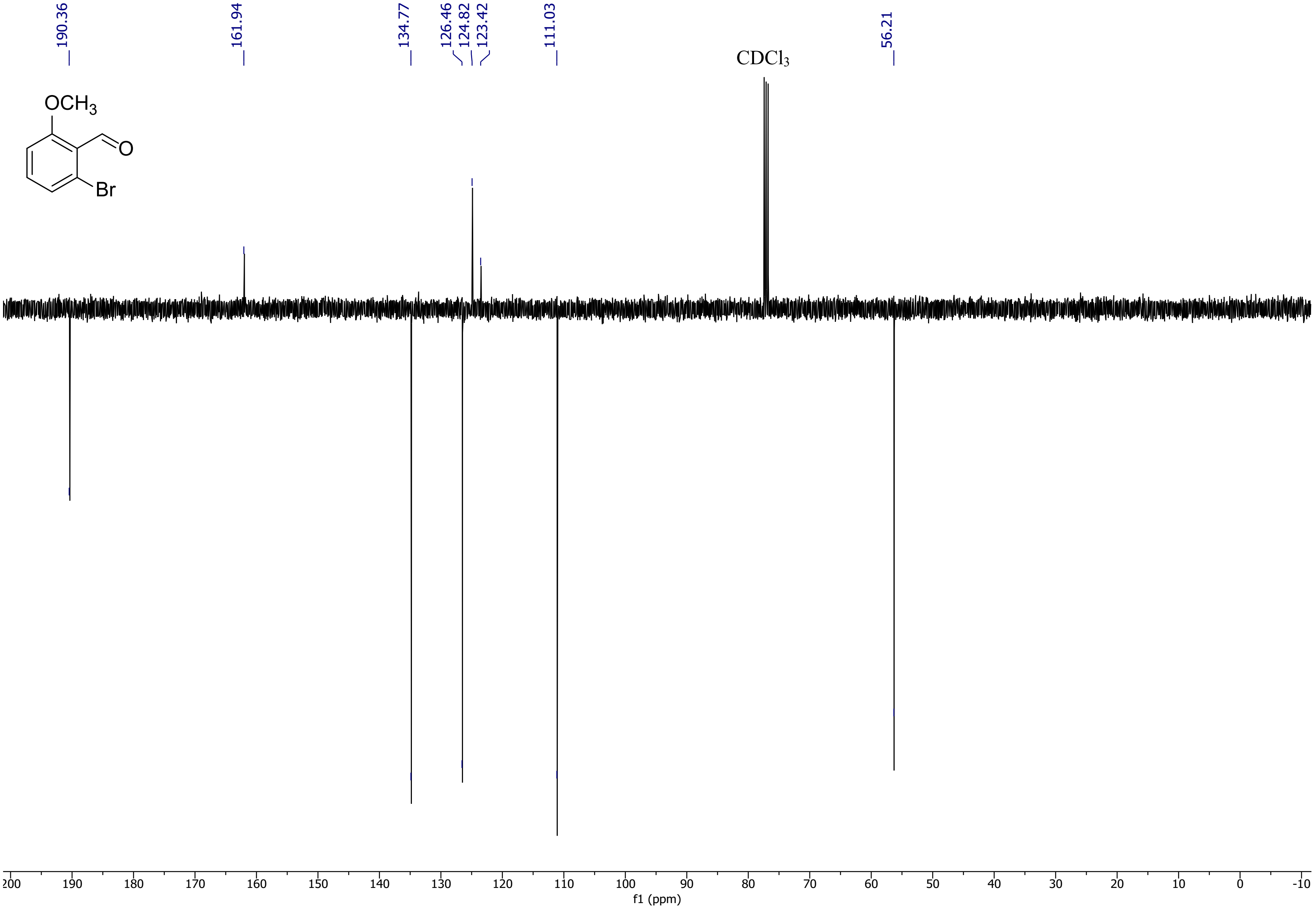

^{1}H-NMR (400 MHz, $CDCl_3$)

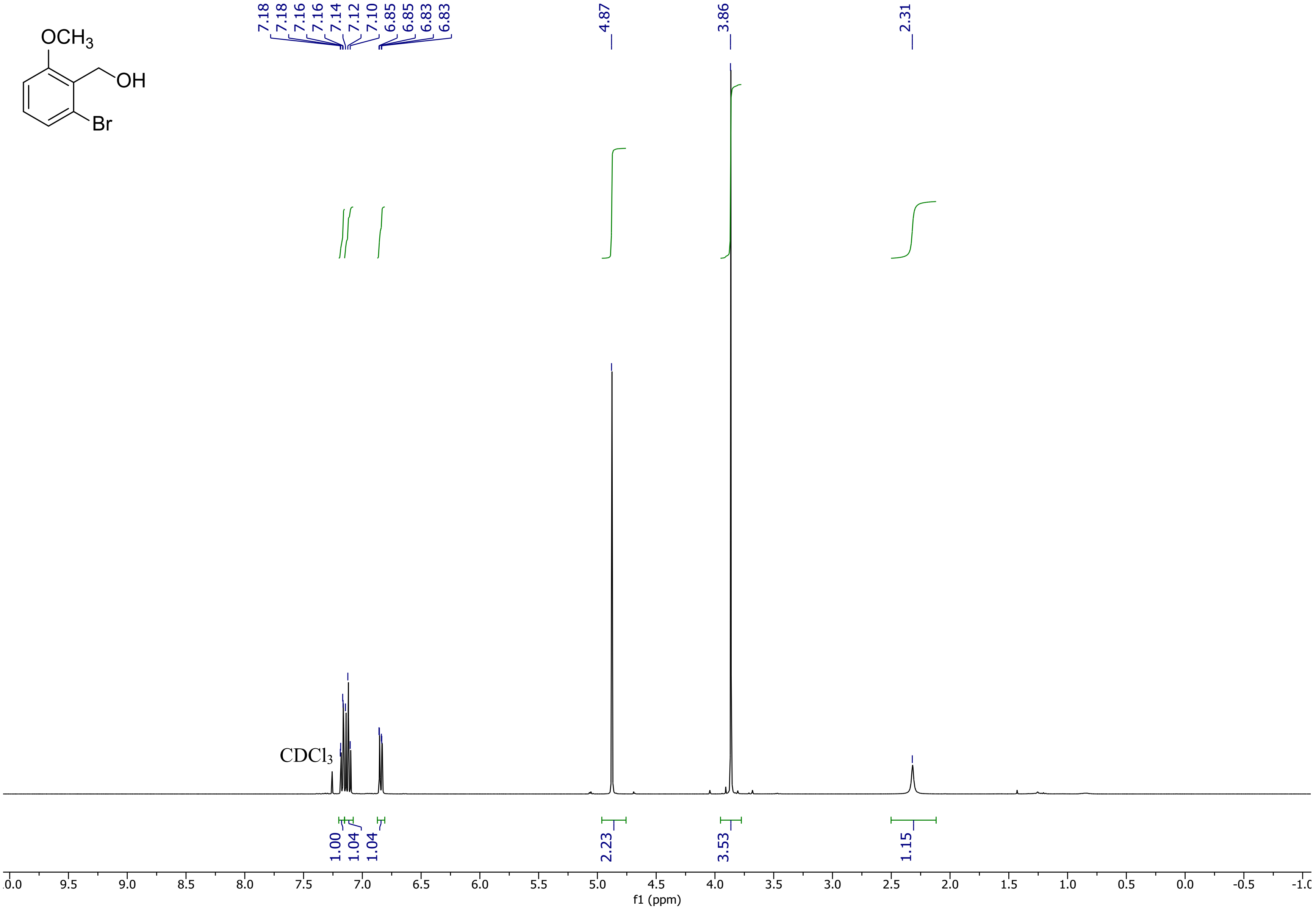

$^{13}C\{^{1}H\}$-NMR APT (101 MHz, $CDCl_3$)

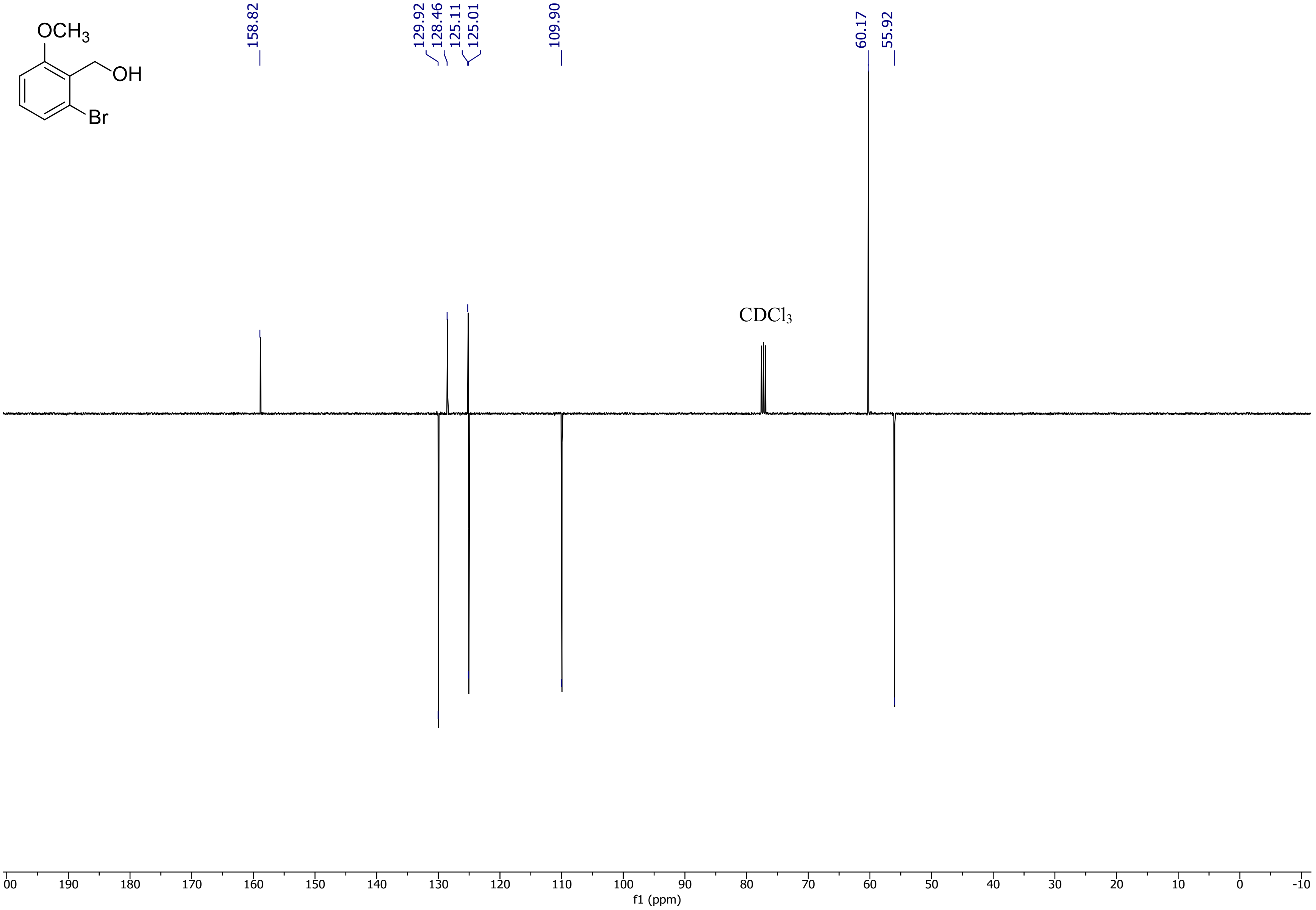

^{1}H-NMR (400 MHz, $CDCl_3$)

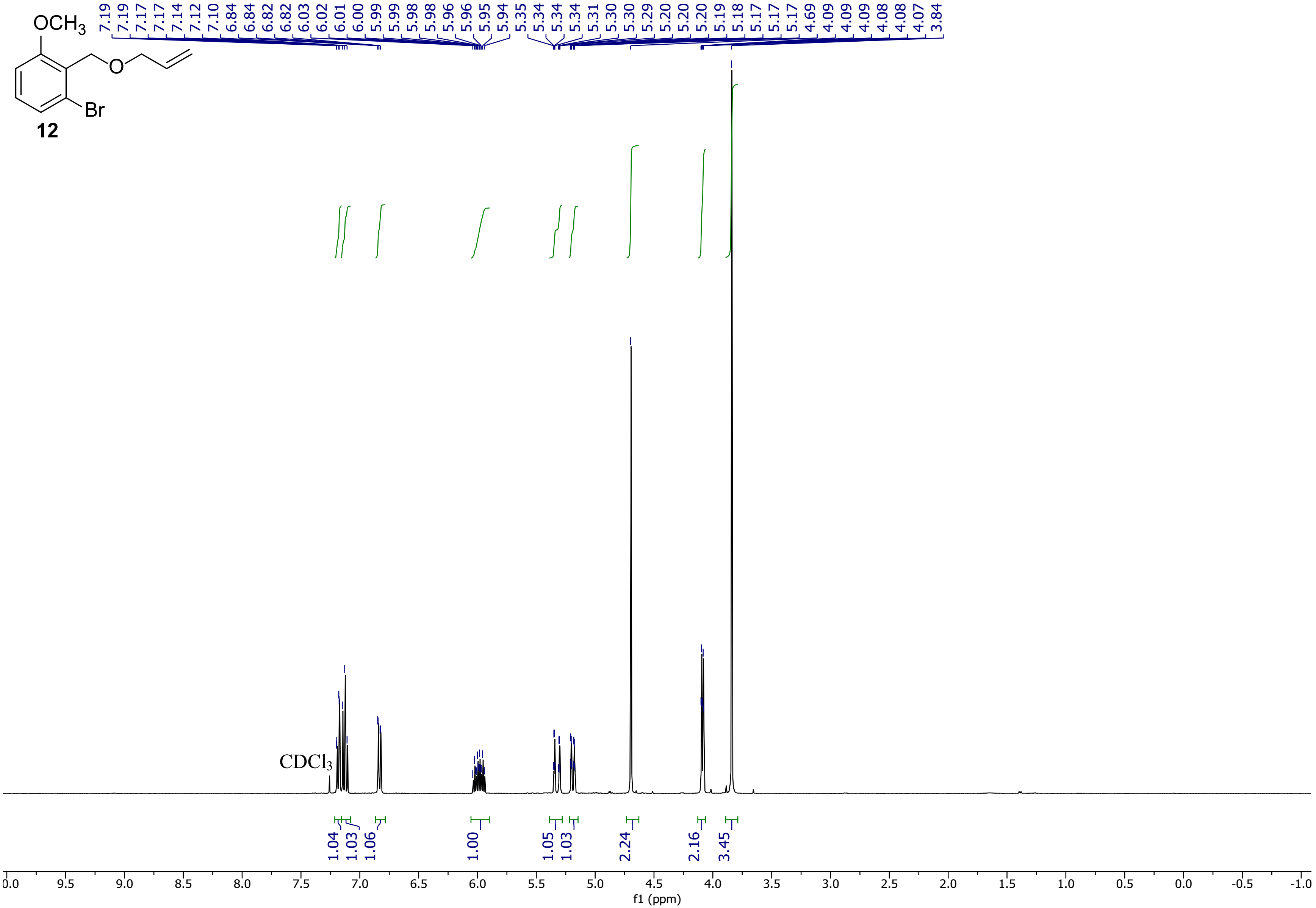

$^{13}C\{^{1}H\}$-NMR APT (101 MHz, $CDCl_3$)

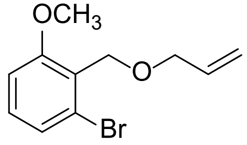

12

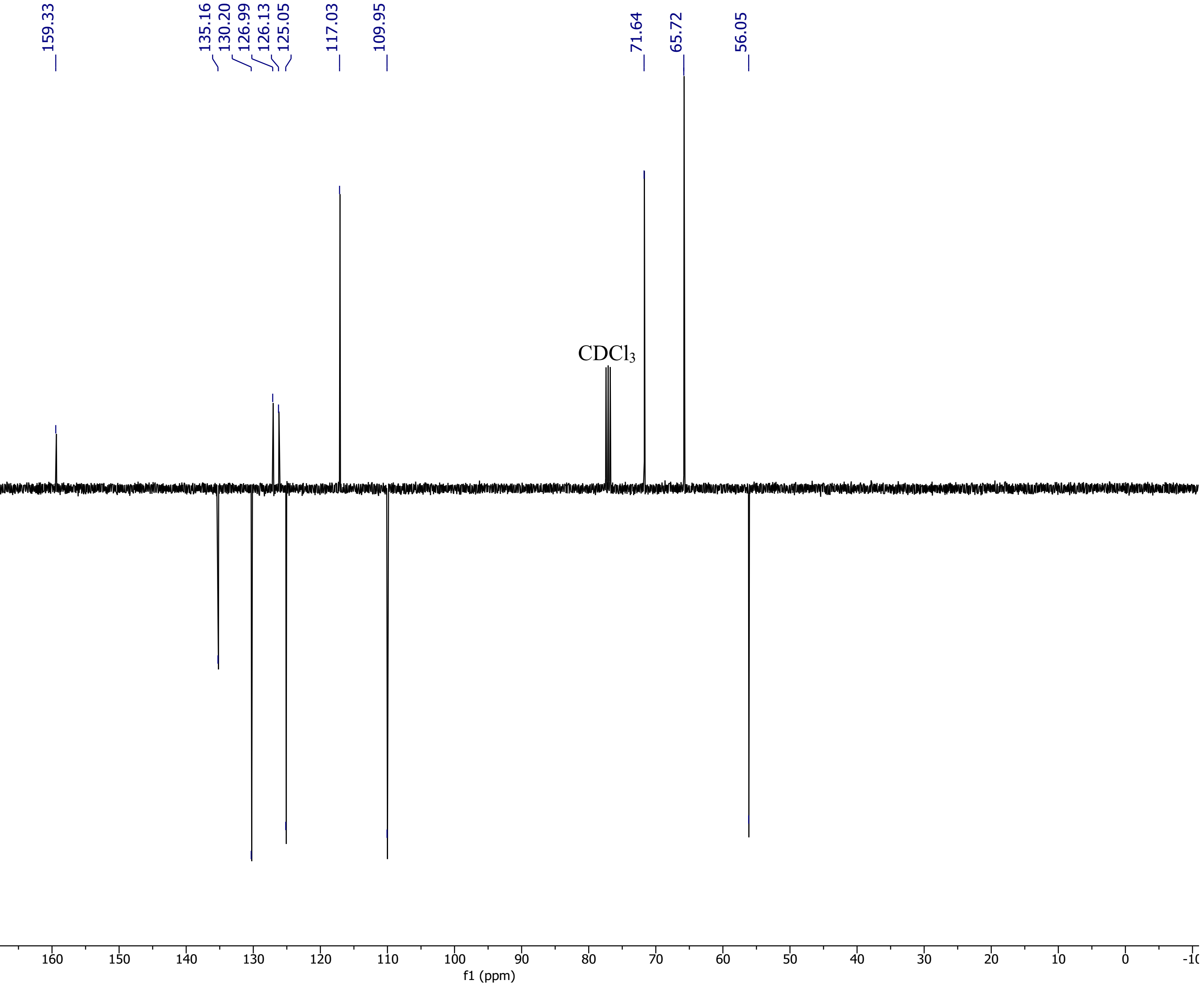

^{1}H-NMR (400 MHz, $CDCl_3$)

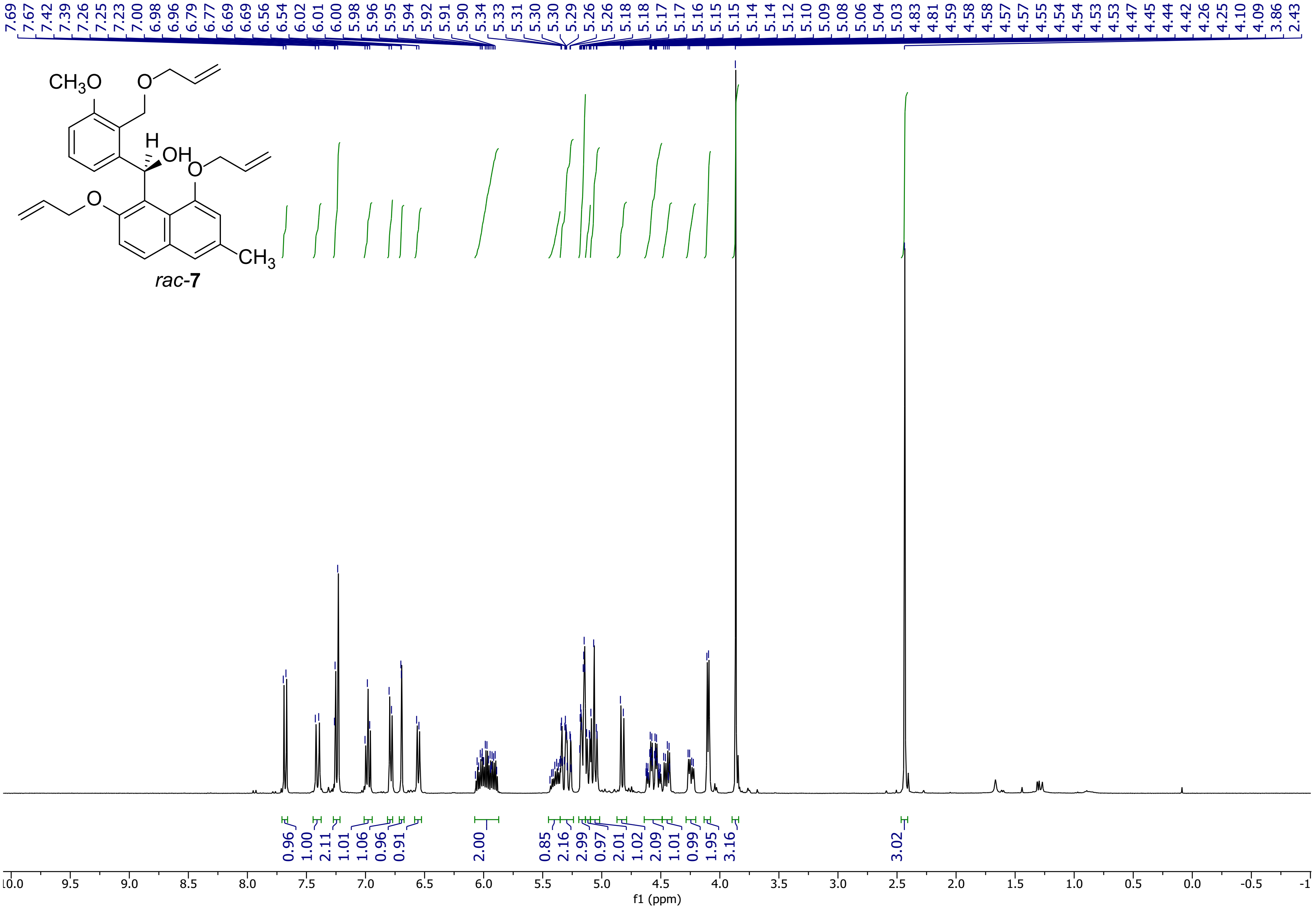

$^{13}C\{^{1}H\}$-NMR APT (101 MHz, $CDCl_3$)

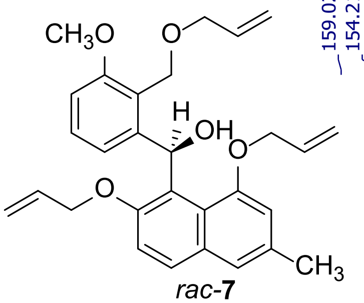

rac-**7**

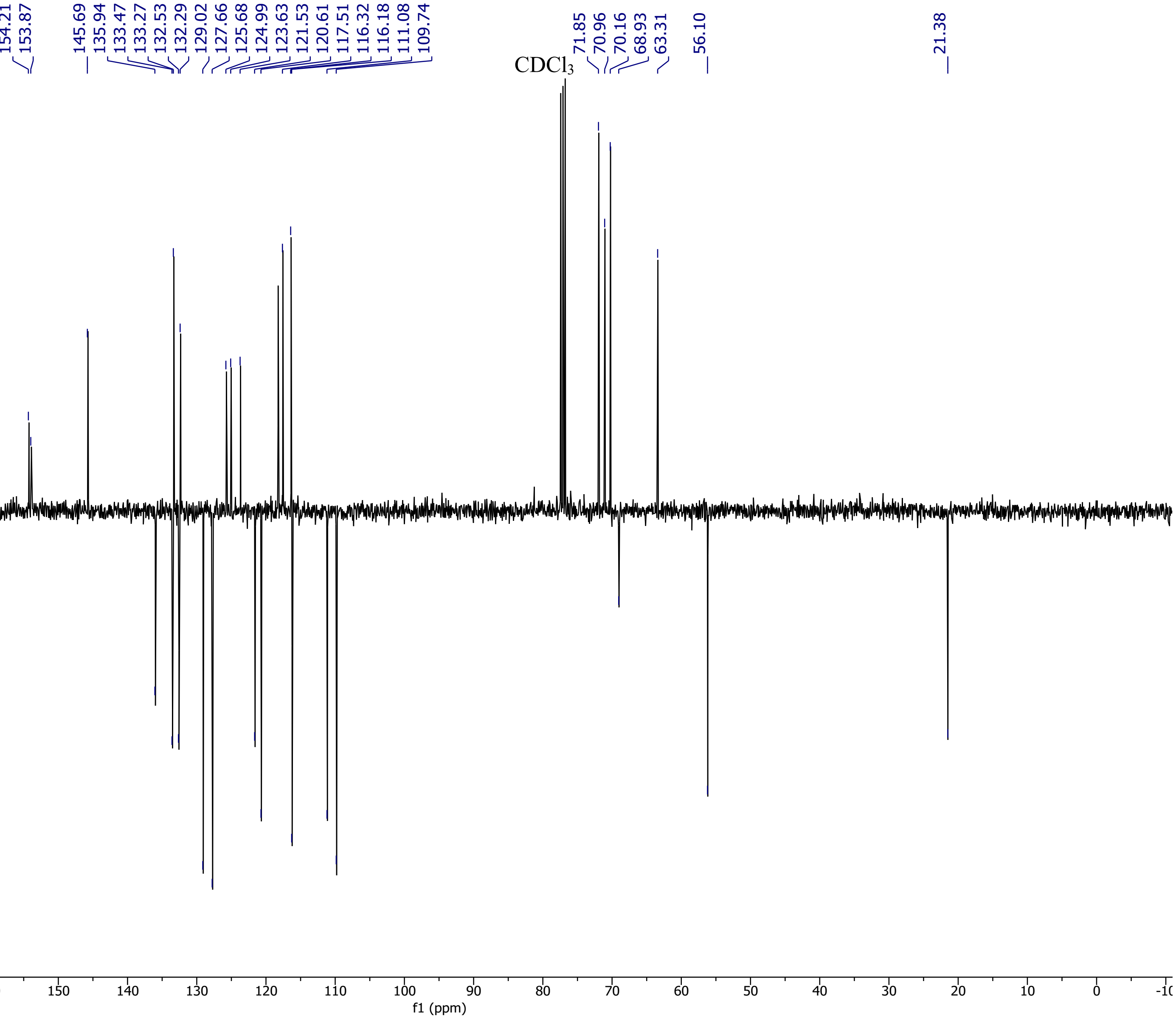

^{1}H-NMR (400 MHz, $CDCl_3$)

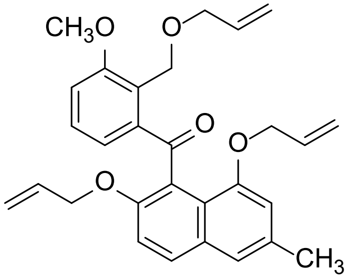

24

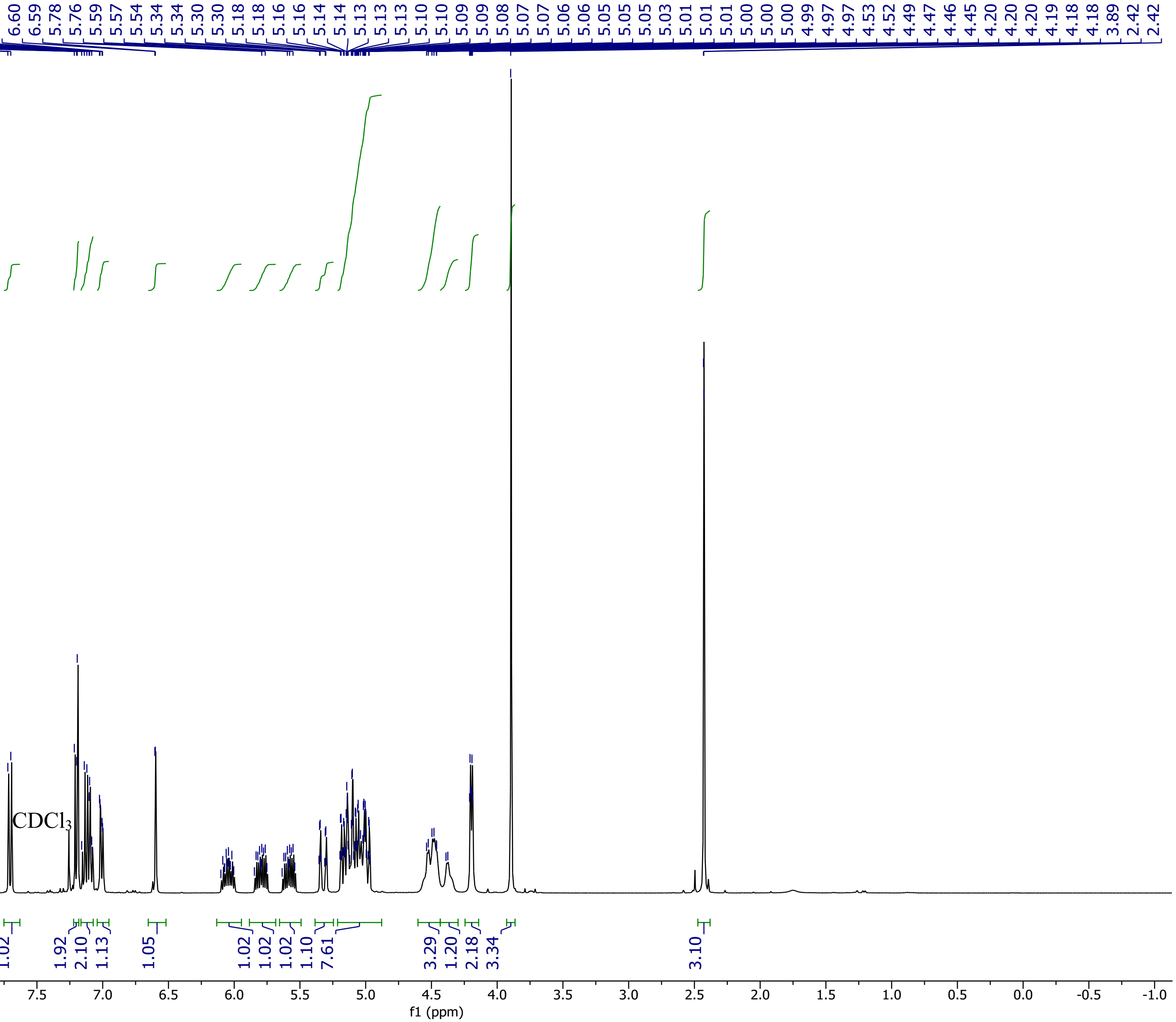

$^{13}C\{^1H\}$-NMR APT (101 MHz, $CDCl_3$)

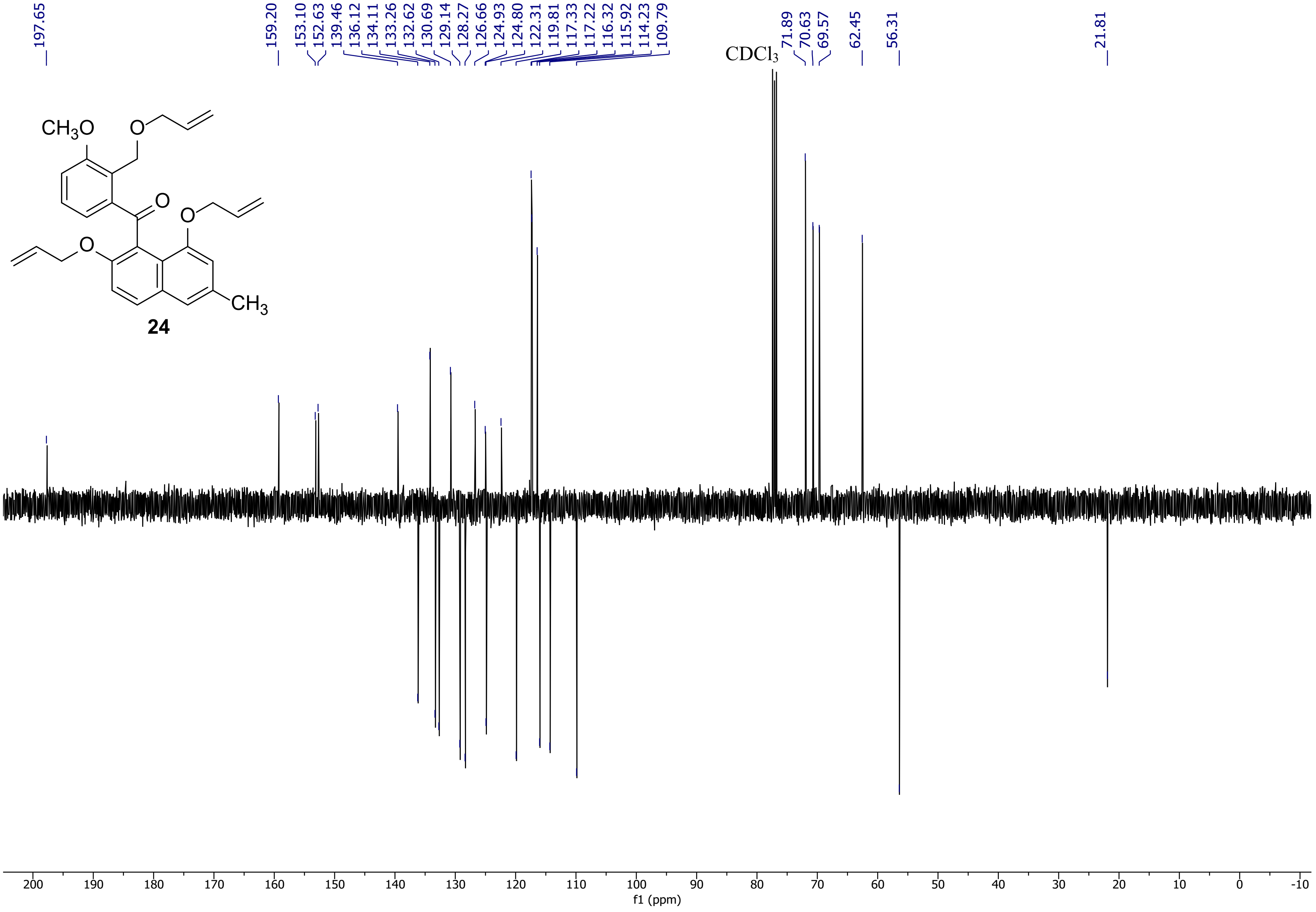

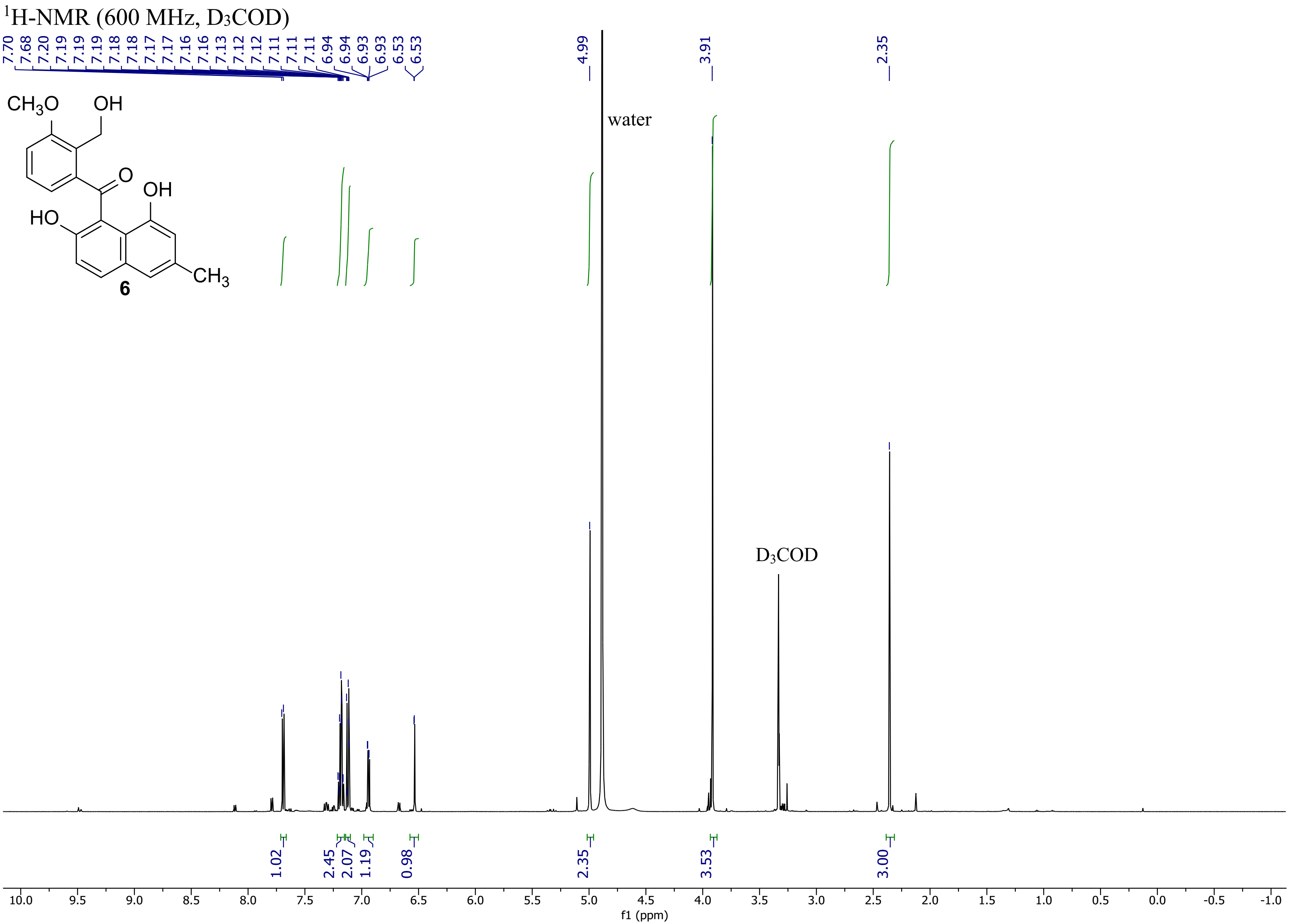

1H-NMR (600 MHz, D3COD)
7.70 7.68 7.20 7.19 7.19 7.19 7.18 7.18 7.17 7.17 7.16 7.16 7.13 7.12 7.12 7.11 7.11 7.11 6.94 6.94 6.93 6.93 6.53 6.53
4.99
3.91
2.35
CH3O
OH
O
OH
HO
CH3
6
water
D3COD
1.02
2.45
2.07
1.19
0.98
2.35
3.53
3.00
10.0 9.5 9.0 8.5 8.0 7.5 7.0 6.5 6.0 5.5 5.0 4.5 4.0 3.5 3.0 2.5 2.0 1.5 1.0 0.5 0.0 -0.5 -1.0
f1 (ppm)

$^{13}C\{^{1}H\}$-NMR APT (151 MHz, D_3COD)

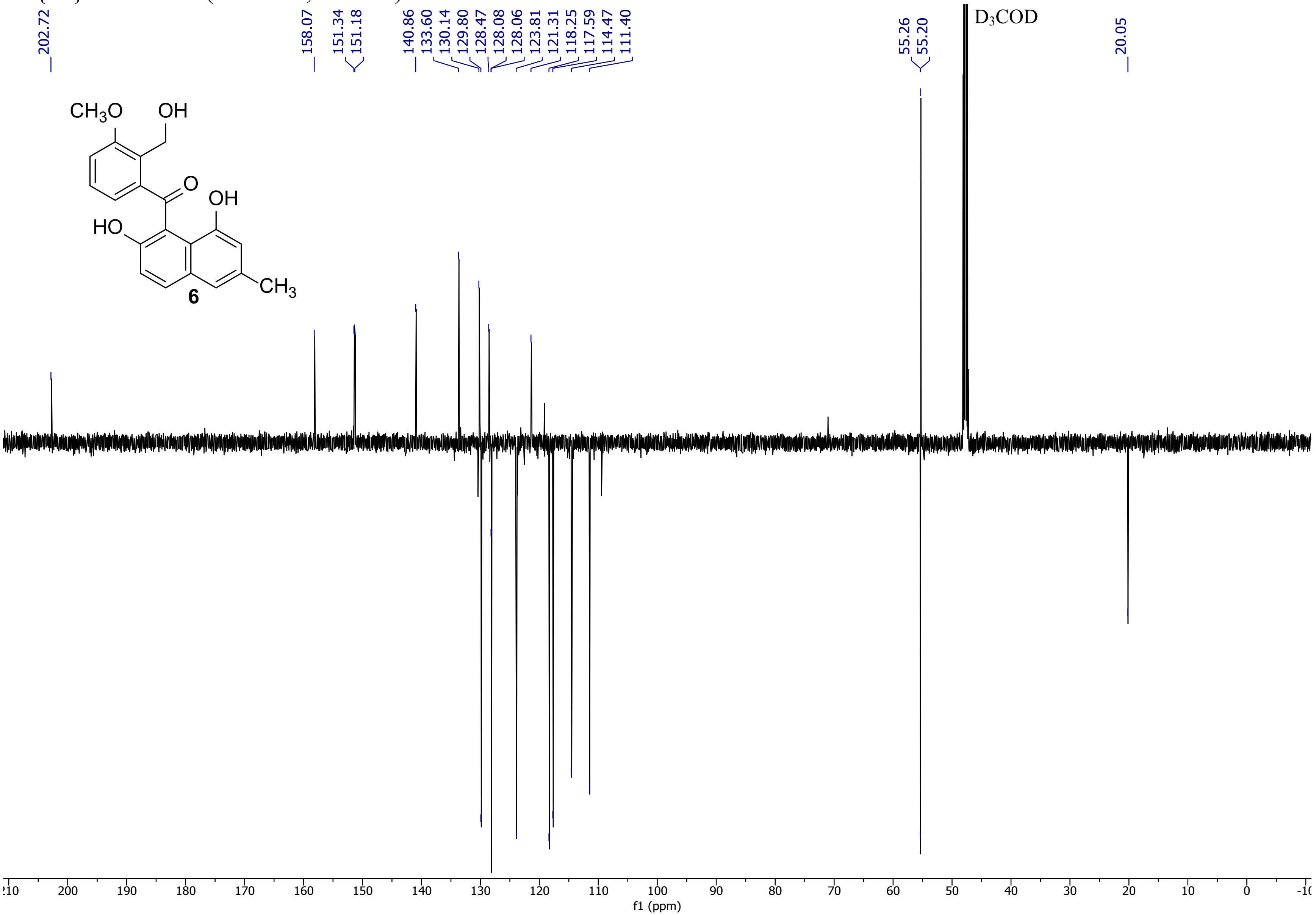

^{1}H-NMR (400 MHz, $(D_3C)_2CO$)

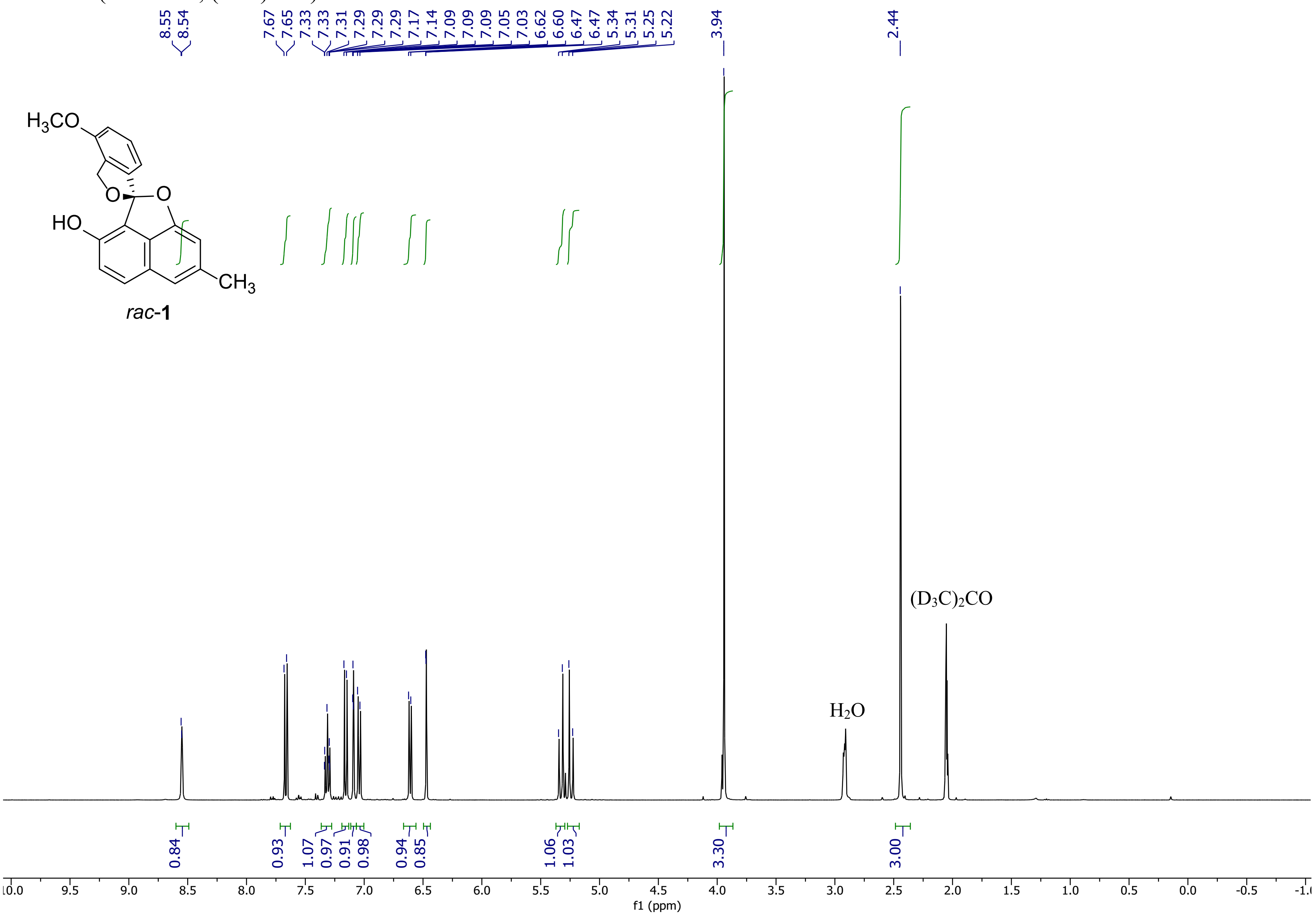

48

$^{13}C\{^{1}H\}$-NMR APT (101 MHz, $(D_3C)_2CO$)

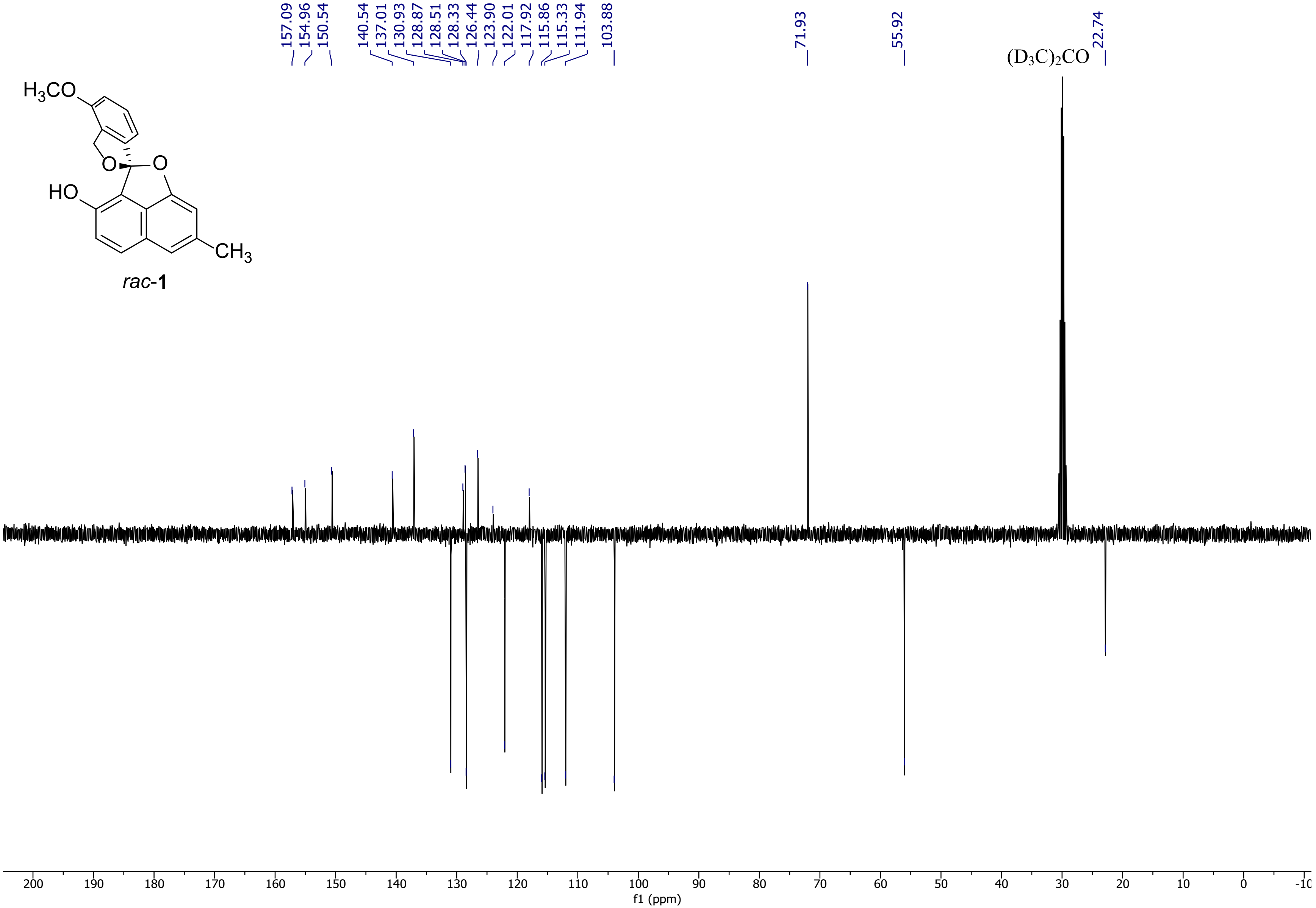

rac-**1**

^{1}H-NMR (400 MHz, $CDCl_3$)

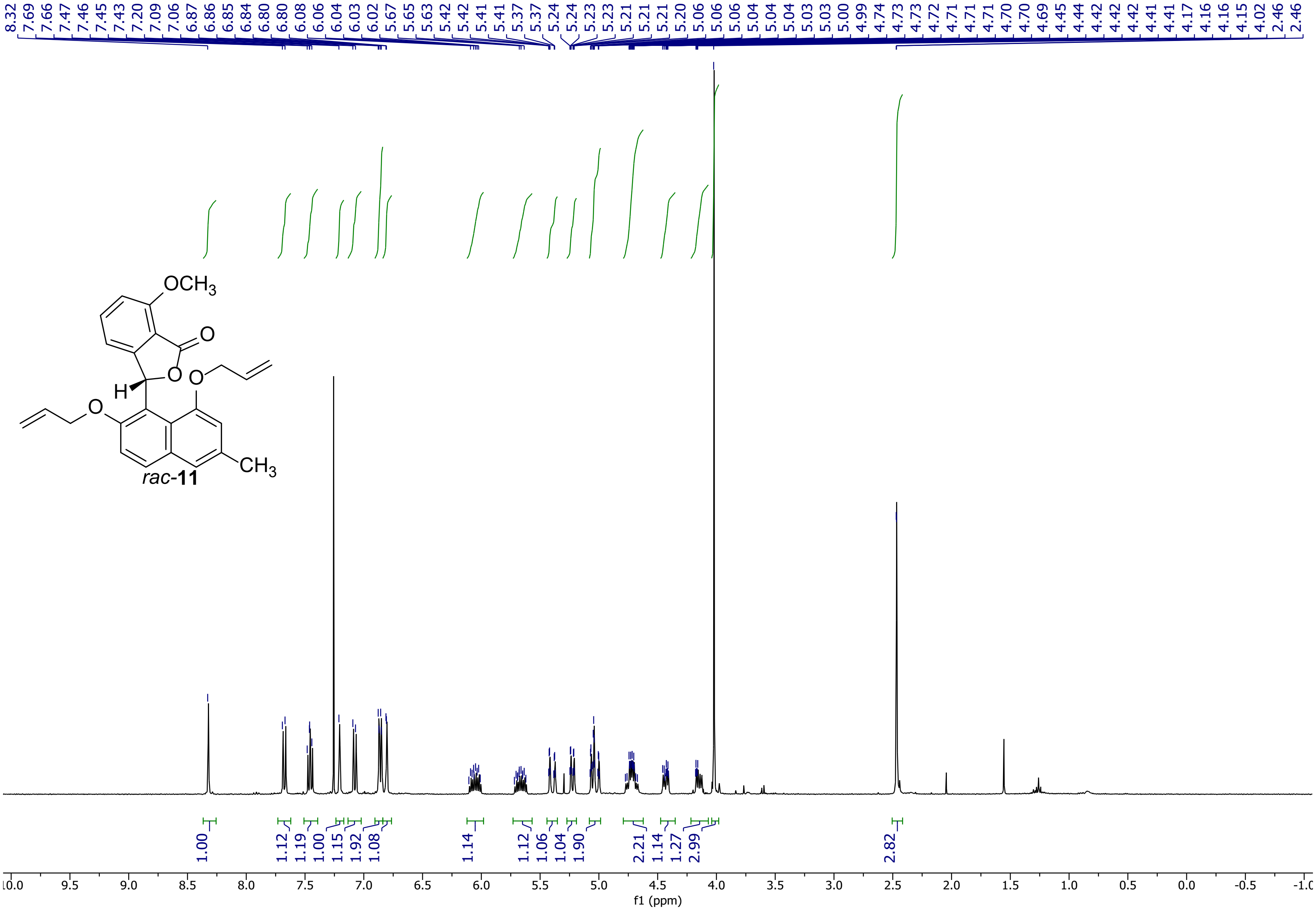

$^{13}C\{^{1}H\}$-NMR APT (101 MHz, $CDCl_3$)

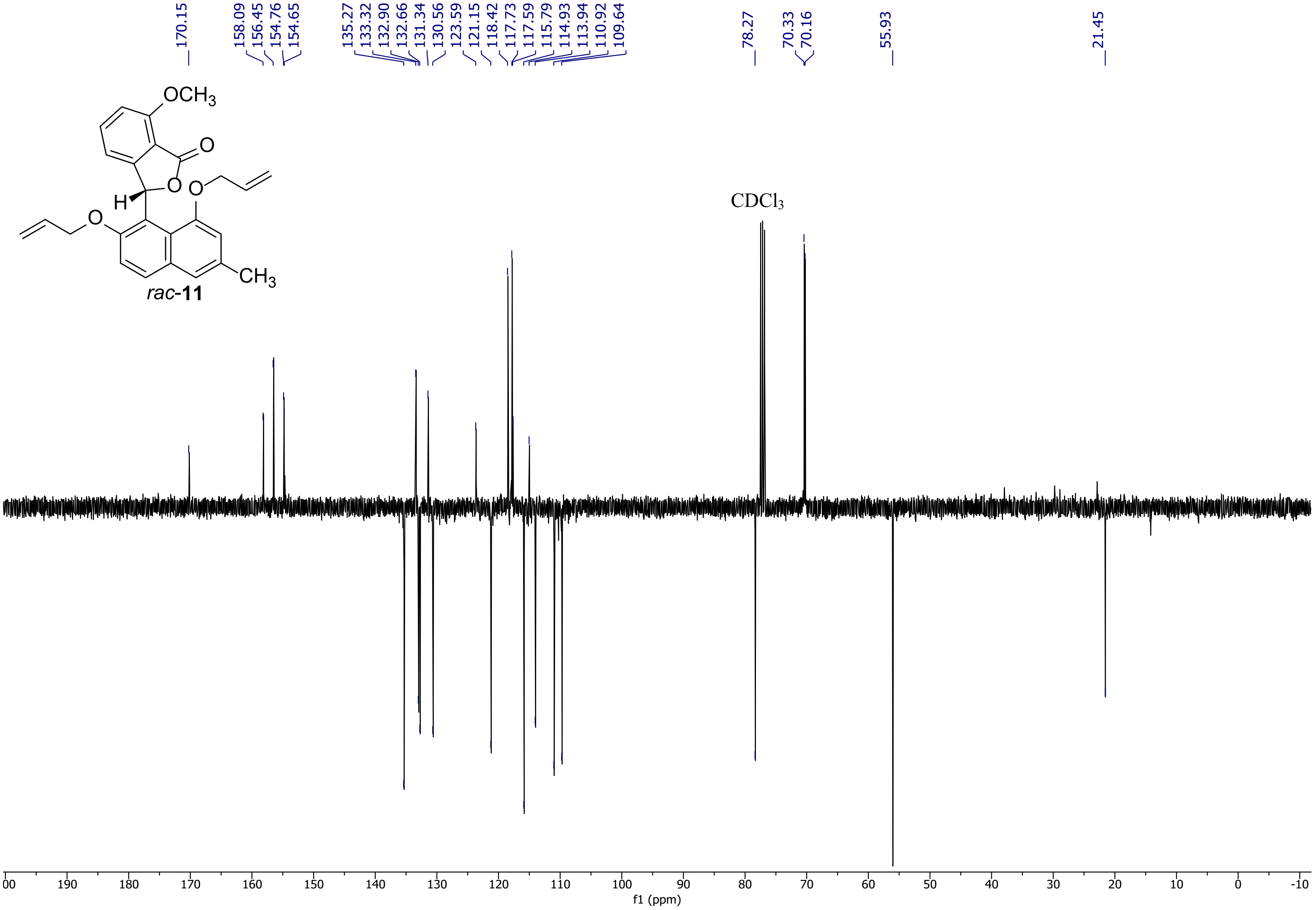

^{1}H-NMR (600 MHz, $(D_3C)_2SO$), after repeated trituration with toluene.

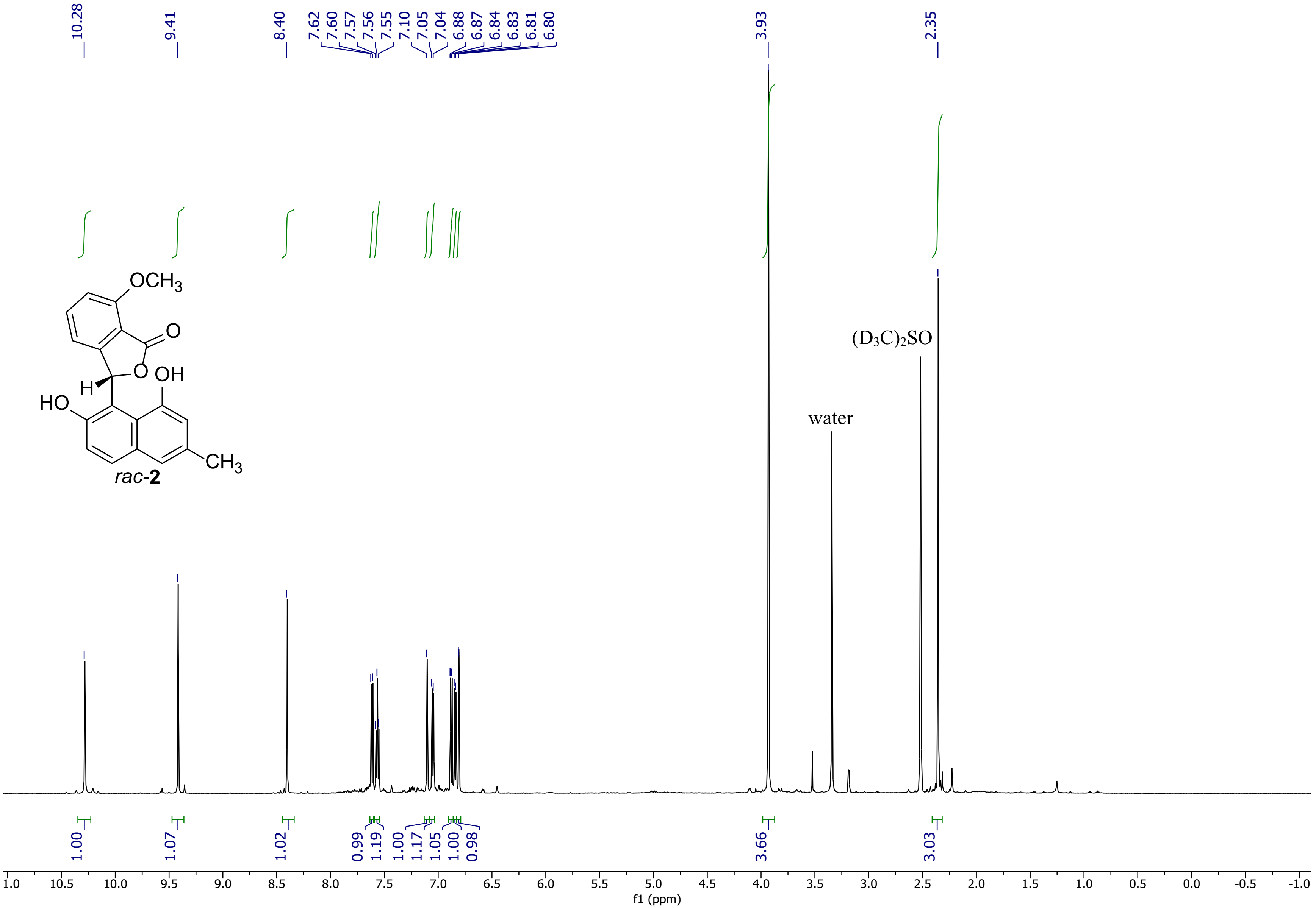

$^{13}C\{^{1}H\}$-NMR APT (151 MHz,$(D_3C)_2SO$), after repeated trituration with toluene.

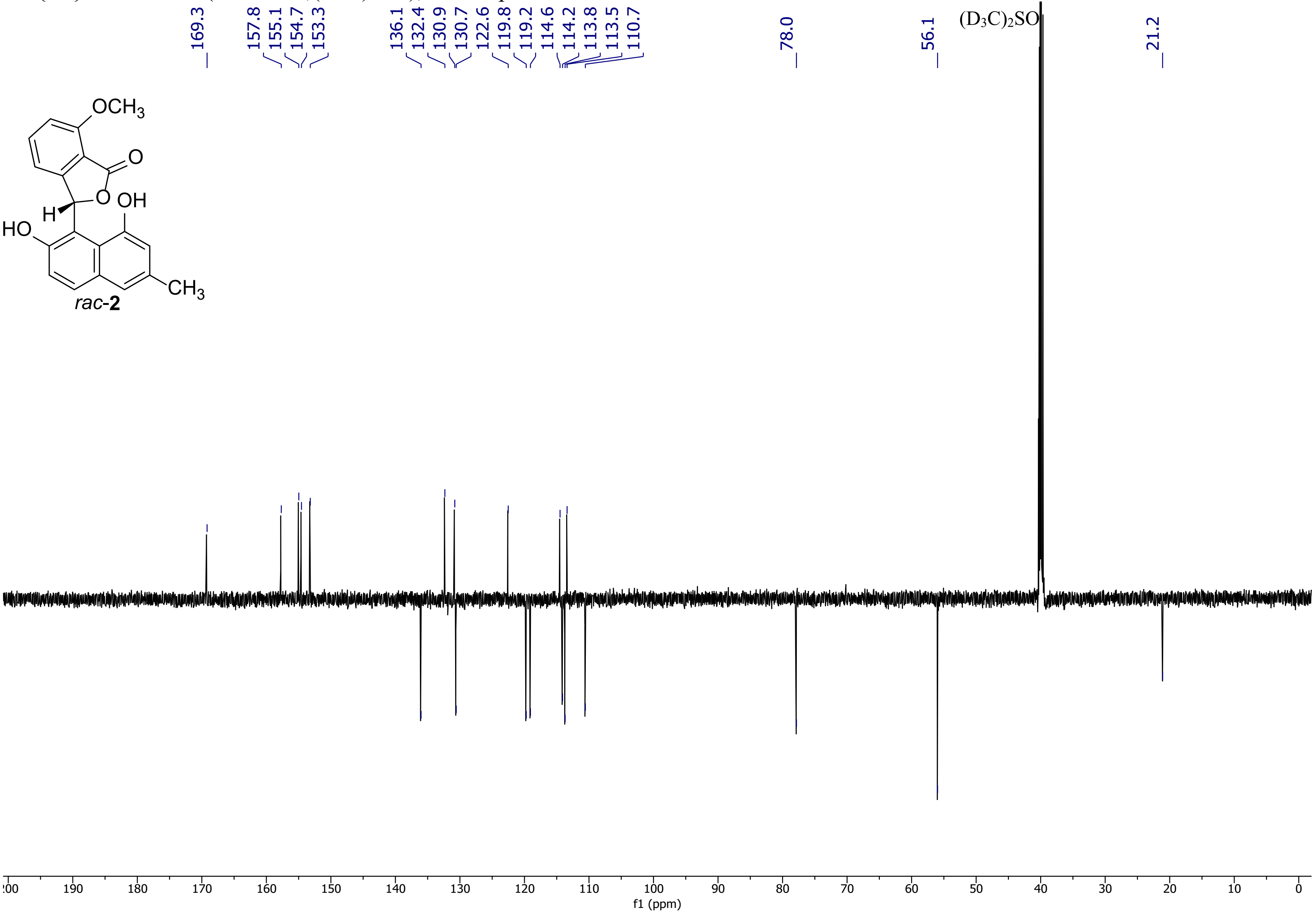

S3: ESI HRMS spectra

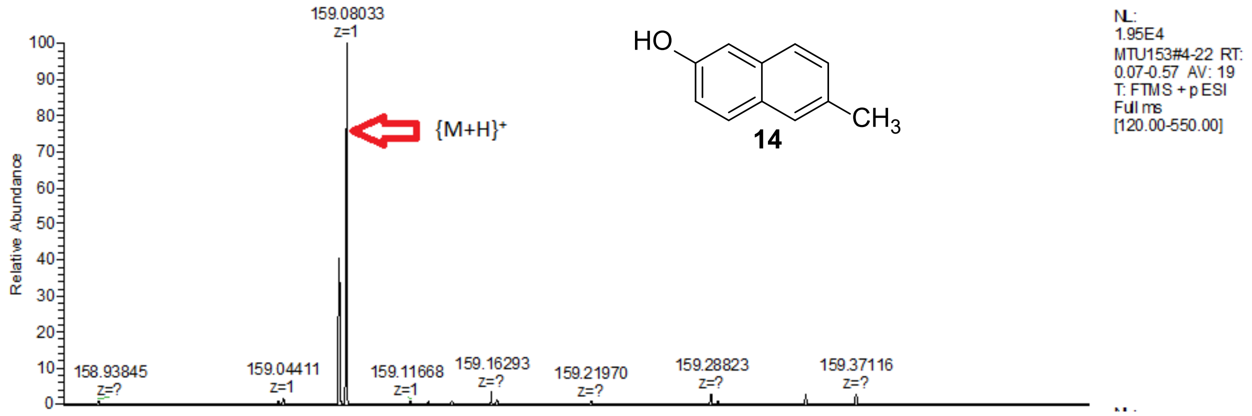

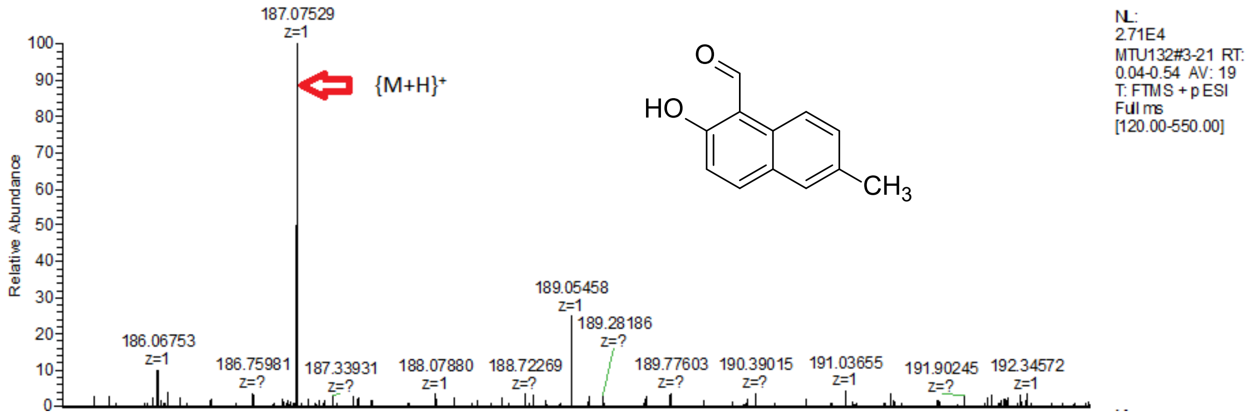

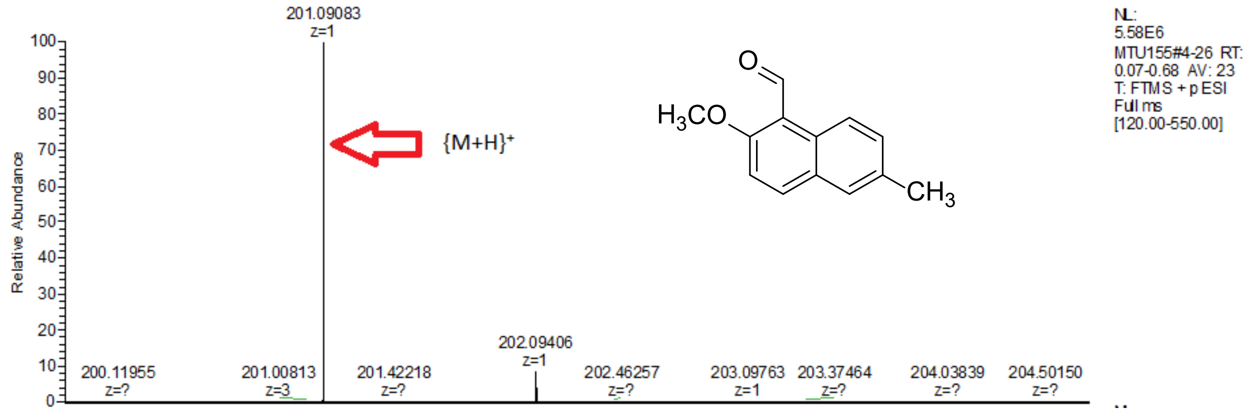

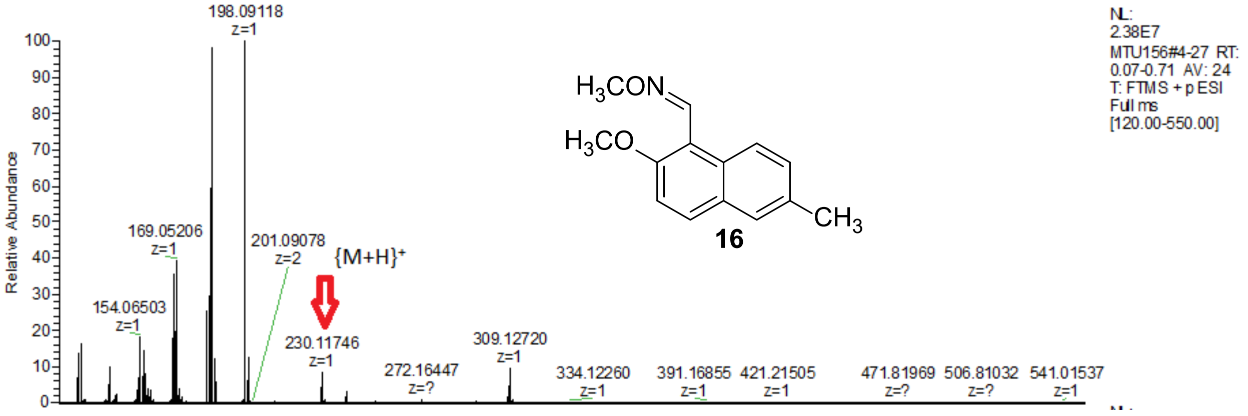

198.09118 z=1
169.05206 z=1
154.06503 z=1
201.09078 z=2
{M+H}+
230.11746 z=1
272.16447 z=?
309.12720 z=1
334.12260 z=1
391.16855 z=1
421.21505 z=1
471.81969 z=?
506.81032 z=?
541.01537 z=1
Relative Abundance
NL: 2.38E7 MTU156#4-27 RT: 0.07-0.71 AV: 24 T: FTMS + p ESI Full ms [120.00-550.00]
H3CON
H3CO
CH3
16

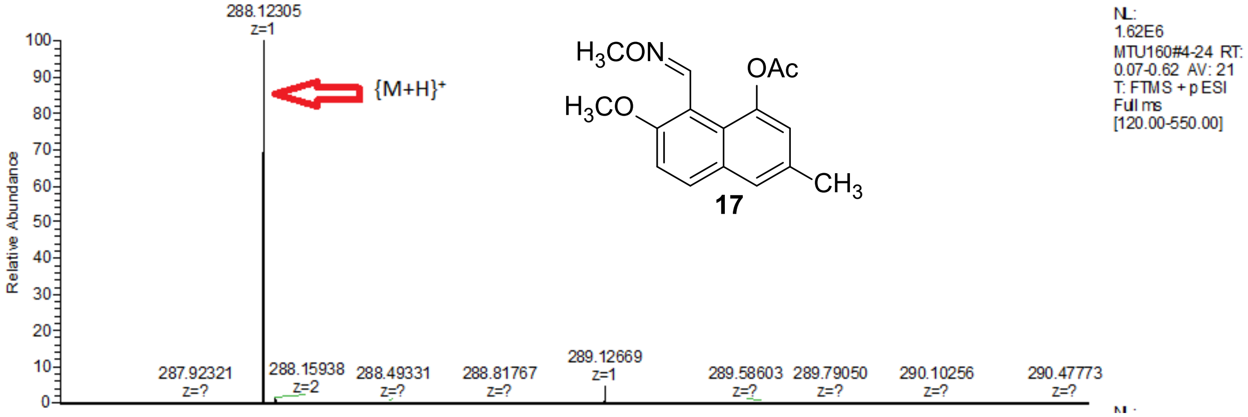

288.12305 z=1
{M+H}+
287.92321 z=?
288.15938 z=2
288.49331 z=?
288.81767 z=?
289.12669 z=1
289.58603 z=?
289.79050 z=?
290.10256 z=?
290.47773 z=?
Relative Abundance
NL: 1.62E6 MTU160#4-24 RT: 0.07-0.62 AV: 21 T: FTMS + p ESI Full ms [120.00-550.00]
H3CON
OAc
H3CO
CH3
17

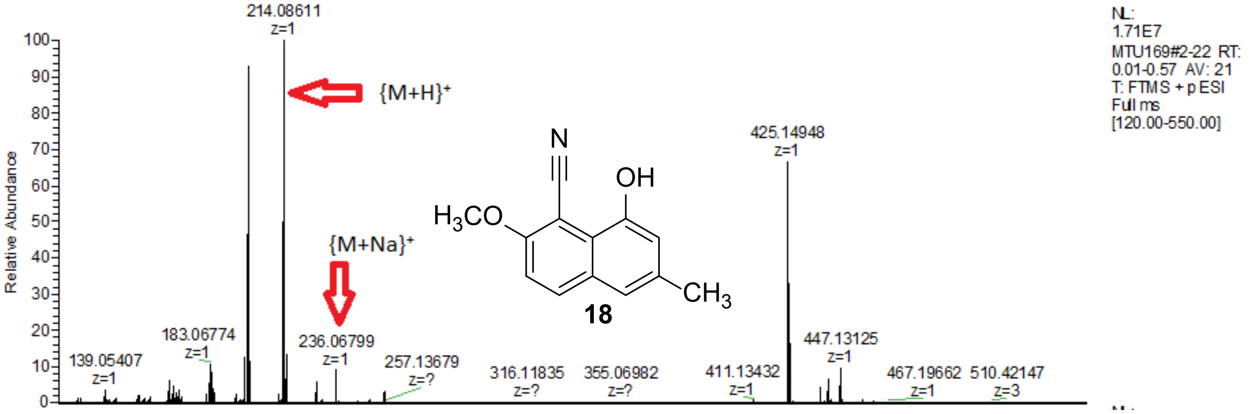

214.08611 z=1
{M+H}+
{M+Na}+
139.05407 z=1
183.06774 z=1
236.06799 z=1
257.13679 z=?
316.11835 z=?
355.06982 z=?
411.13432 z=1
425.14948 z=1
447.13125 z=1
467.19662 z=1
510.42147 z=3
Relative Abundance
NL: 1.71E7 MTU169#2-22 RT: 0.01-0.57 AV: 21 T: FTMS + p ESI Full ms [120.00-550.00]
N
OH
H3CO
CH3
18

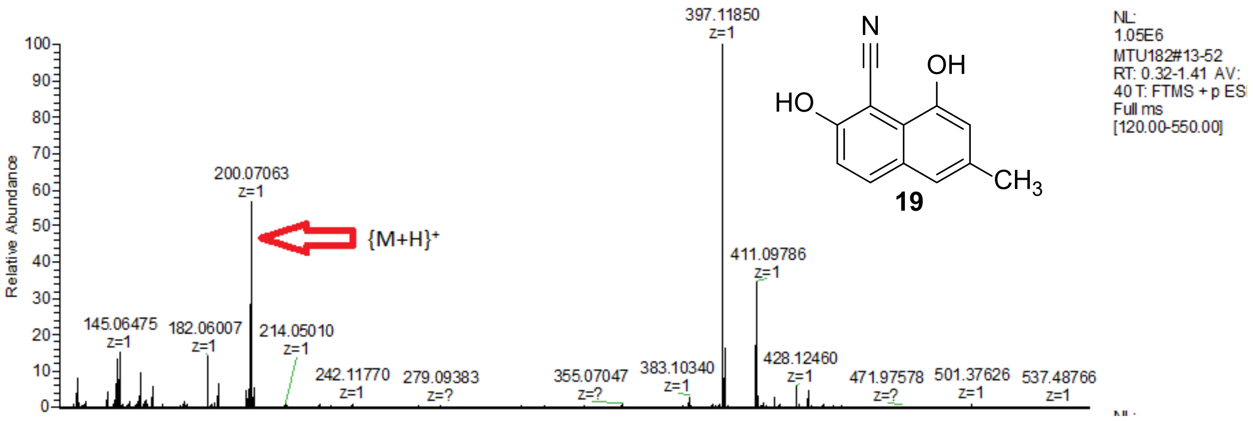
397.11850
z=1
200.07063
z=1
{M+H}+
411.09786
z=1
145.06475
z=1
182.06007
z=1
214.05010
z=1
242.11770
z=1
279.09383
z=?
355.07047
z=?
383.10340
z=1
428.12460
z=1
471.97578
z=?
501.37626
z=1
537.48766
z=1
Relative Abundance
N
OH
HO
CH3
19
NL:
1.05E6
MTU182#13-52
RT: 0.32-1.41 AV:
40 T: FTMS + p ESI
Full ms
[120.00-550.00]

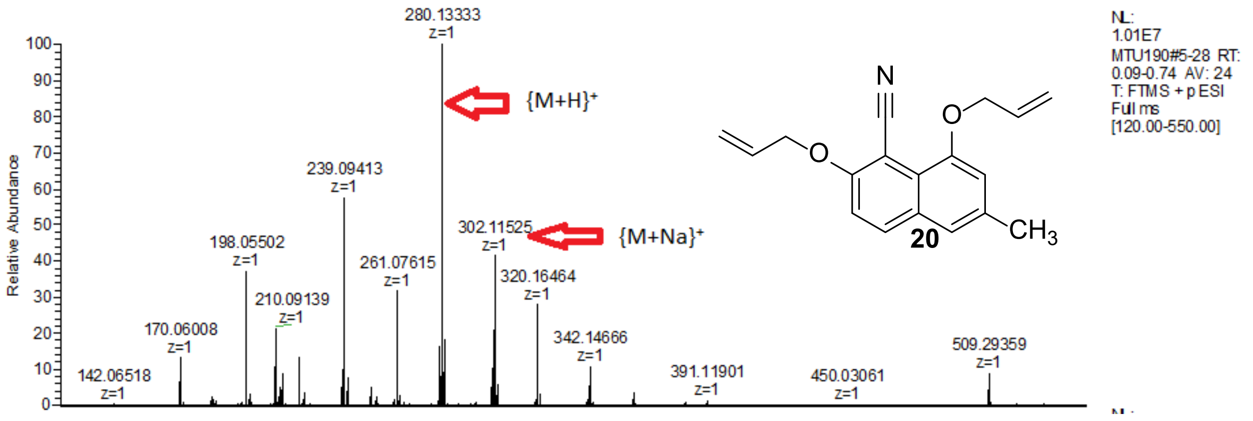
280.13333
z=1
{M+H}+
239.09413
z=1
302.11525
z=1
{M+Na}+
198.05502
z=1
261.07615
z=1
320.16464
z=1
210.09139
z=1
170.06008
z=1
342.14666
z=1
142.06518
z=1
391.11901
z=1
450.03061
z=1
509.29359
z=1
Relative Abundance
N
O
O
CH3
20
NL:
1.01E7
MTU190#5-28 RT:
0.09-0.74 AV: 24
T: FTMS + p ESI
Full ms
[120.00-550.00]

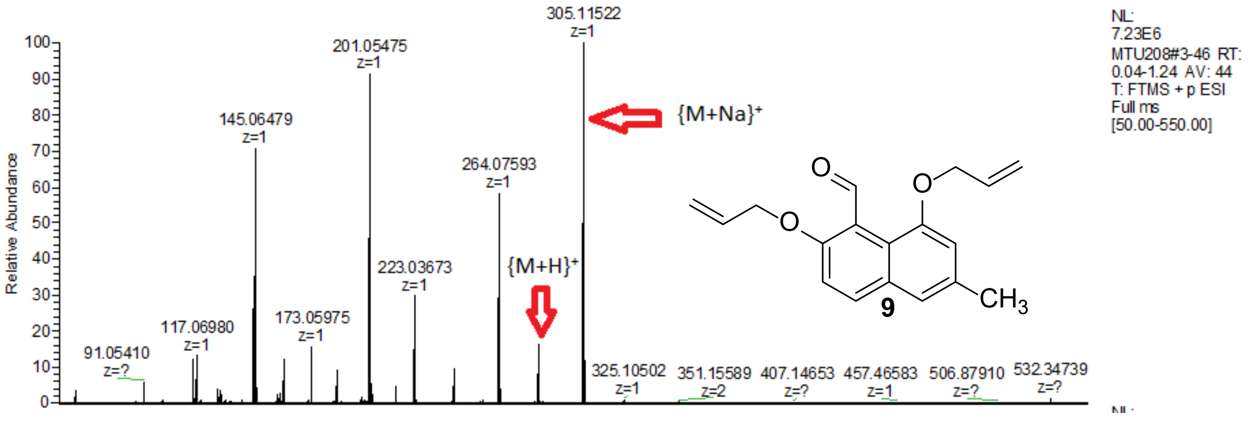
305.11522
z=1
201.05475
z=1
{M+Na}+
145.06479
z=1
264.07593
z=1
223.03673
z=1
{M+H}+
117.06980
z=1
173.05975
z=1
91.05410
z=?
325.10502
z=1
351.15589
z=2
407.14653
z=?
457.46583
z=1
506.87910
z=?
532.34739
z=?
Relative Abundance
O
O
O
CH3
9
NL:
7.23E6
MTU208#3-46 RT:
0.04-1.24 AV: 44
T: FTMS + p ESI
Full ms
[50.00-550.00]

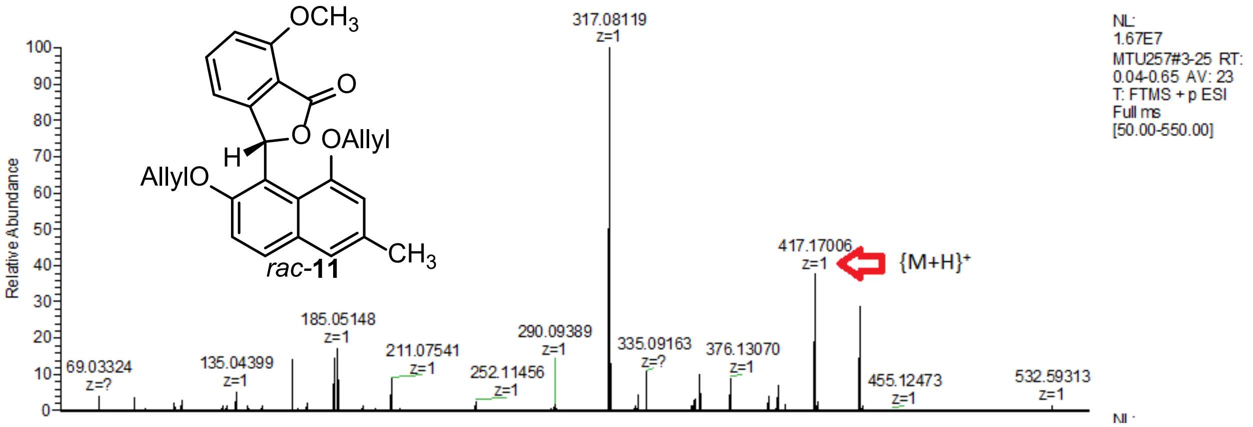
OCH3
O
H
O
OAllyl
AllylO
CH3
rac-11
Relative Abundance
317.08119
z=1
417.17006
z=1
{M+H}+
NL:
1.67E7
MTU257#3-25 RT:
0.04-0.65 AV: 23
T: FTMS + p ESI
Full ms
[50.00-550.00]
69.03324
z=?
135.04399
z=1
185.05148
z=1
211.07541
z=1
252.11456
z=1
290.09389
z=1
335.09163
z=?
376.13070
z=1
455.12473
z=1
532.59313
z=1

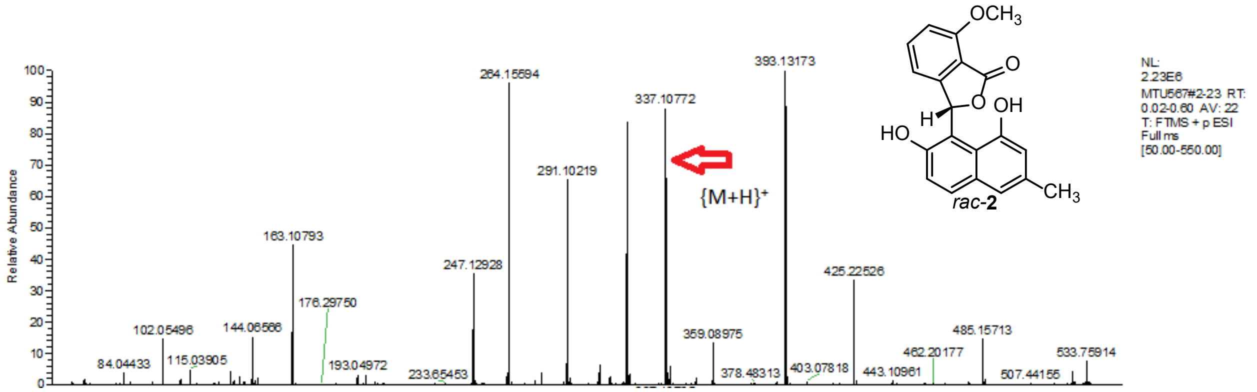
OCH3
O
H
O
OH
HO
CH3
rac-2
Relative Abundance
264.15594
337.10772
393.13173
{M+H}+
291.10219
163.10793
247.12928
425.22526
176.29750
102.05496
144.06566
359.08975
485.15713
84.04433
115.03905
193.04972
233.65463
378.48313
403.07818
443.10961
462.20177
507.44155
533.75914
NL:
2.23E6
MTU567#2-23 RT:
0.02-0.60 AV: 22
T: FTMS + p ESI
Full ms
[50.00-550.00]

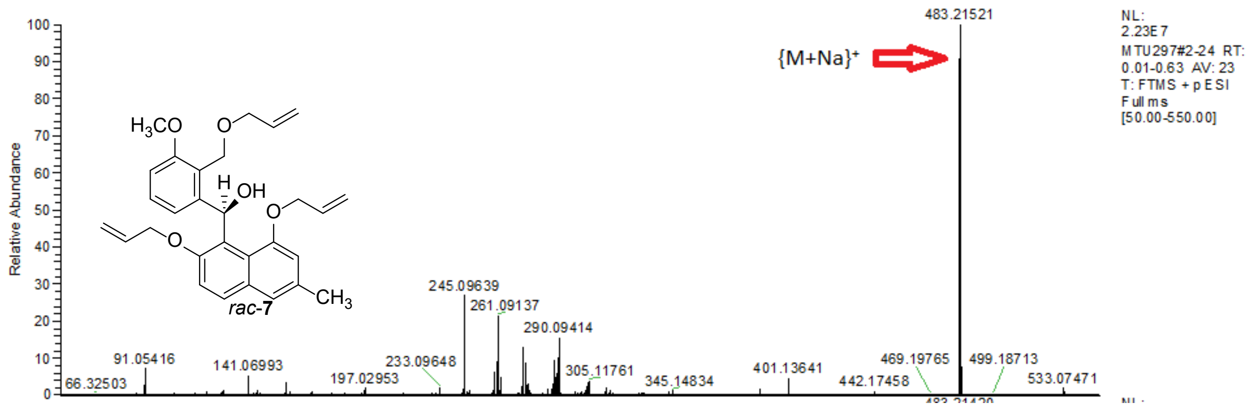
483.21521
{M+Na}+
NL:
2.23E7
MTU297#2-24 RT:
0.01-0.63 AV: 23
T: FTMS + p ESI
Full ms
[50.00-550.00]
H3CO
O
H
OH
O
O
CH3
rac-7
Relative Abundance
245.09639
261.09137
290.09414
91.05416
141.06993
233.09648
305.11761
345.14834
401.13641
469.19765
499.18713
66.32503
197.02953
442.17458
533.07471

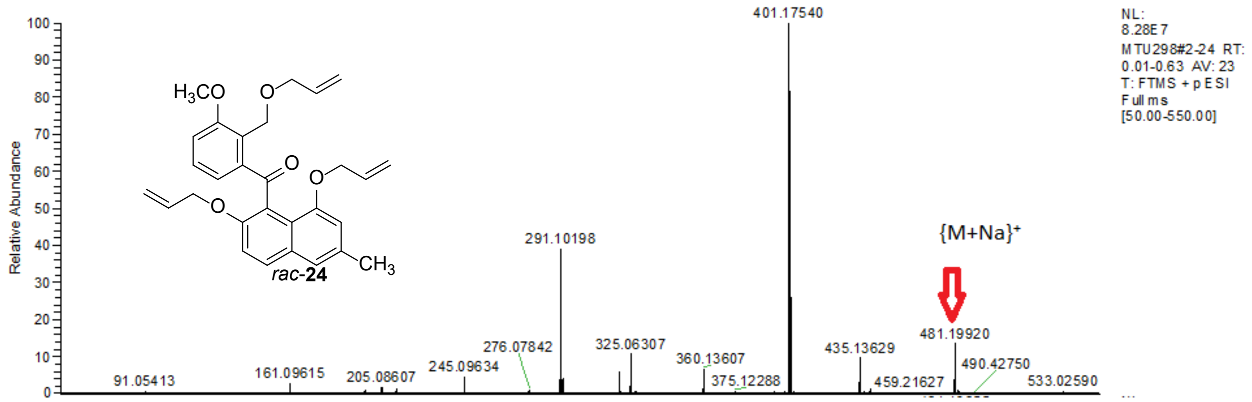
NL:
8.28E7
MTU298#2-24 RT:
0.01-0.63 AV: 23
T: FTMS + p ESI
Full ms
[50.00-550.00]
Relative Abundance
rac-24
{M+Na}+
401.17540
291.10198
481.19920
490.42750
533.02590
459.21627
435.13629
375.12288
360.13607
325.06307
276.07842
245.09634
205.08607
161.09615
91.05413

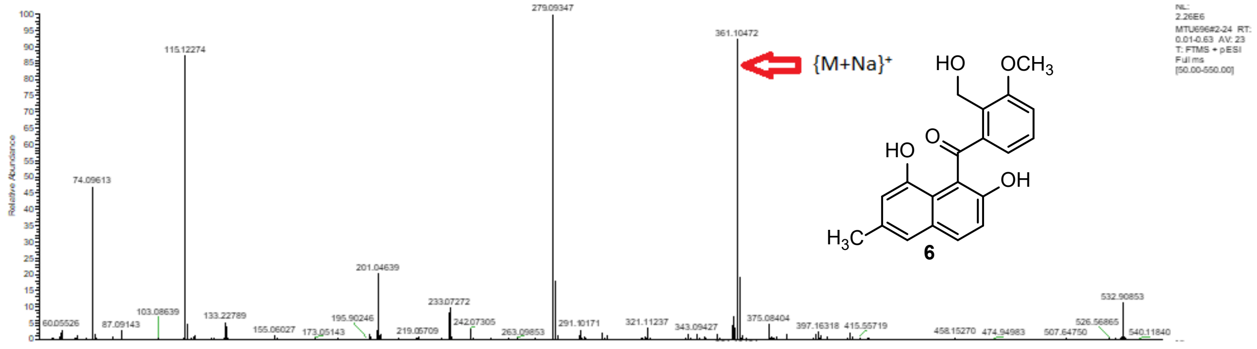
279.09347
361.10472
115.12274
74.09613
201.04639
233.07272
532.90853
{M+Na}+
6

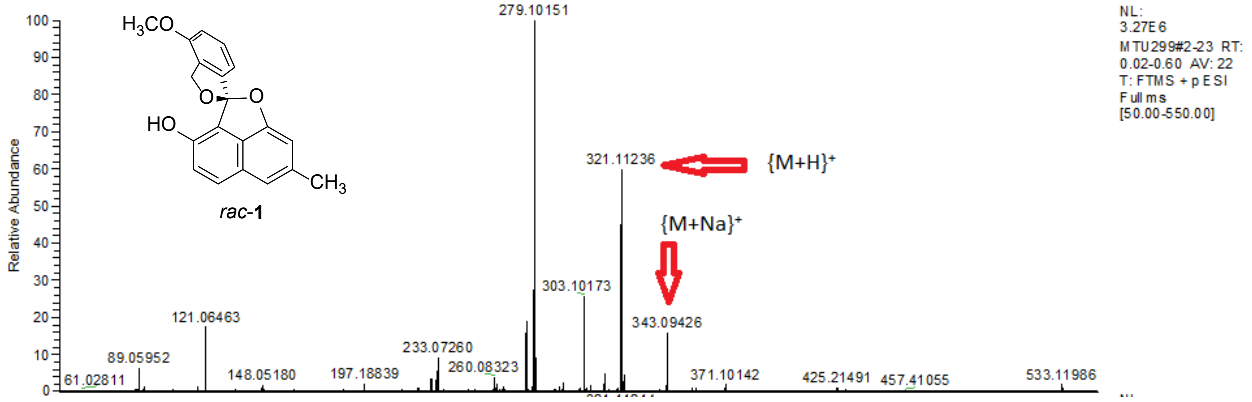
NL:
3.27E6
MTU299#2-23 RT:
0.02-0.60 AV: 22
T: FTMS + p ESI
Full ms
[50.00-550.00]
Relative Abundance
rac-1
279.10151
321.11236
{M+H}+
{M+Na}+
303.10173
343.09426
121.06463
233.07260
260.08323
89.05952
61.02811
148.05180
197.18839
371.10142
425.21491
457.41055
533.11986

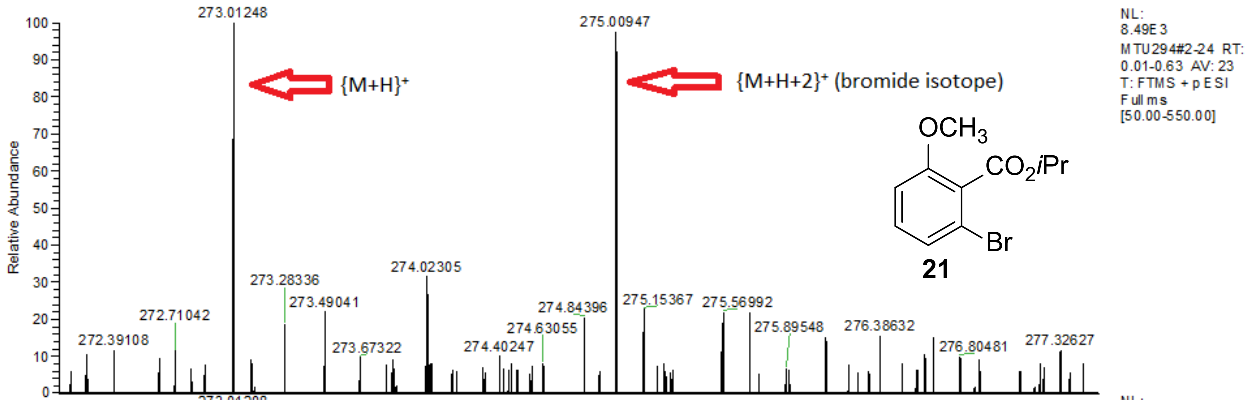
273.01248
275.00947
{M+H}+
{M+H+2}+ (bromide isotope)
OCH3
CO2iPr
Br
21
Relative Abundance
NL:
8.49E3
MTU294#2-24 RT:
0.01-0.63 AV: 23
T: FTMS + p ESI
Full ms
[50.00-550.00]

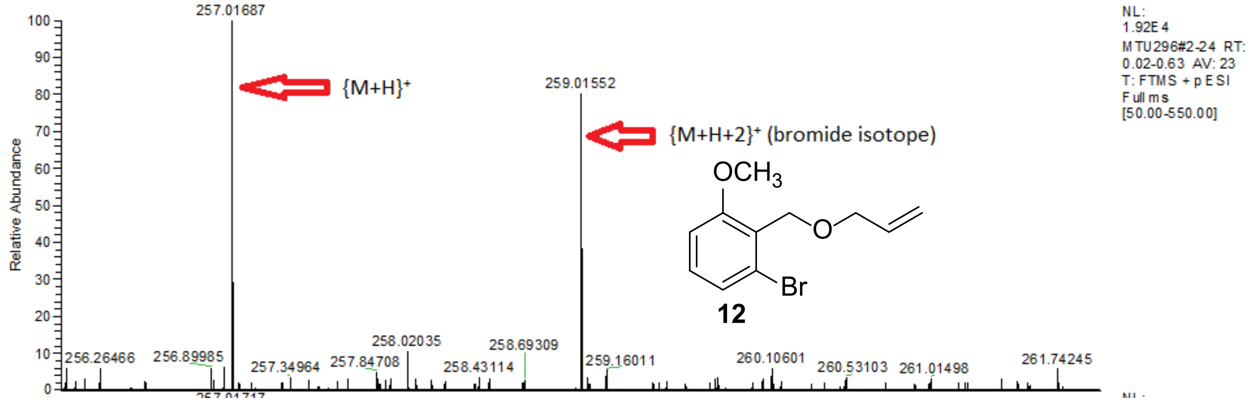
257.01687
259.01552
{M+H}+
{M+H+2}+ (bromide isotope)
OCH3
O
Br
12
Relative Abundance
NL:
1.92E4
MTU296#2-24 RT:
0.02-0.63 AV: 23
T: FTMS + p ESI
Full ms
[50.00-550.00]
